# Jets Downstream of Collisionless Shocks

**DOI:** 10.1007/s11214-018-0516-3

**Published:** 2018-06-21

**Authors:** Ferdinand Plaschke, Heli Hietala, Martin Archer, Xóchitl Blanco-Cano, Primož Kajdič, Tomas Karlsson, Sun Hee Lee, Nojan Omidi, Minna Palmroth, Vadim Roytershteyn, Daniel Schmid, Victor Sergeev, David Sibeck

**Affiliations:** 1 Space Research Institute, Austrian Academy of Sciences, Graz, Austria; 2 Present Address: Institute of Physics, University of Graz, Graz, Austria; 3 Department of Earth, Planetary, and Space Sciences, University of California Los Angeles, Los Angeles, CA USA; 4 School of Physics and Astronomy, Queen Mary University of London, London, UK; 5 Instituto de Geofísica, Universidad Nacional Autónoma de México, México City, Mexico; 6 Space and Plasma Physics, School of Electrical Engineering, KTH Royal Institute of Technology, Stockholm, Sweden; 7 Goddard Space Flight Center, National Aeronautics and Space Administration, Greenbelt, MD USA; 8 Solana Scientific Inc., Solana Beach, CA USA; 9 Department of Physics, University of Helsinki, Helsinki, Finland; 10 Earth Observation, Finnish Meteorological Institute, Helsinki, Finland; 11 Space Science Institute, Boulder, CO USA; 12 Harbin Institute of Technology, Shenzhen, China; 13 Sankt Petersburg State University, Sankt Petersburg, Russia

**Keywords:** Jets, Magnetosheath, Foreshock, Bow shock, Magnetopause

## Abstract

The magnetosheath flow may take the form of large amplitude, yet spatially localized, transient increases in dynamic pressure, known as “magnetosheath jets” or “plasmoids” among other denominations. Here, we describe the present state of knowledge with respect to such jets, which are a very common phenomenon downstream of the quasi-parallel bow shock. We discuss their properties as determined by satellite observations (based on both case and statistical studies), their occurrence, their relation to solar wind and foreshock conditions, and their interaction with and impact on the magnetosphere. As carriers of plasma and corresponding momentum, energy, and magnetic flux, jets bear some similarities to bursty bulk flows, which they are compared to. Based on our knowledge of jets in the near Earth environment, we discuss the expectations for jets occurring in other planetary and astrophysical environments. We conclude with an outlook, in which a number of open questions are posed and future challenges in jet research are discussed.

## Introduction

**Fig. 1:**
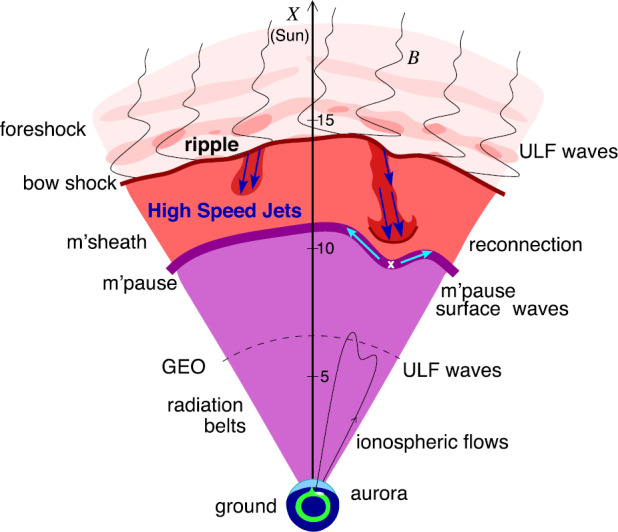
Sketch of the solar wind–magnetosphere–ionosphere system. Jets originating from the bow shock are illustrated and possible consequences of jets impacting the magnetopause are listed. After Fig. 1 in Plaschke et al. (2016)

The magnetosphere, the region dominated by the geomagnetic field and confined by the magnetopause boundary (in dark purple in Fig. 1), acts as an obstacle to the solar wind. In order to circumvent that obstacle, the solar wind has to be decelerated first, from super-magnetosonic to sub-magnetosonic speeds in the Earth’s frame of reference. This happens at the bow shock (in dark red in Fig. 1), located upstream of the magnetopause. The solar wind deflection occurs between the bow shock and the magnetopause, within the magnetosheath region.

Across the bow shock, the solar wind plasma is compressed and heated at the expense of part of the kinetic energy pertaining to its bulk velocity. The changes in magnetic field and plasma moments are, in principle, well-described by the Rankine-Hugoniot relations. Their relations yield boundary conditions to the downstream magnetosheath flow. At the magnetopause, that flow and frozen-in magnetic fields need to be tangential to the boundary. Therewith, it is possible to describe the magnetosheath flow analytically in the framework of hydrodynamic theory (Spreiter et al. 1966).

This description represents well the average magnetosheath flow. It does, however, not take into account the remarkable level of fluctuations of moments and fields that the magnetosheath plasma actually exhibits. The level of fluctuations is highly dependent on the angle $\theta _{Bn}$ between the interplanetary magnetic field (IMF) upstream of the bow shock and the local shock normal direction. Fluctuations are stronger downstream of the quasi-parallel shock ($\theta _{Bn} < 45^{\circ }$) than downstream of the quasi-perpendicular shock ($\theta _{Bn} > 45^{\circ }$).

The quasi-perpendicular shock is relatively thin and well-defined. The rather tangential magnetic fields stabilize the shock wave and prevent particles reflected from the shock from moving upstream in shock normal direction. Upstream of the quasi-parallel shock, instead, a foreshock region exists (see upper part of Fig. 1), penetrated by shock-reflected particles that travel away from the shock along the IMF. They interact with the incoming solar wind, generating waves and large amplitude magnetic structures that steepen and merge into the bow shock. Consequently, the quasi-parallel bow shock is not quiet and well-defined, but patchy and strongly fluctuating due to its continuous formation and reformation (e.g., Schwartz and Burgess 1991; Schwartz 1991; Blanco-Cano et al. 2006a,b).

Despite the downstream plasma being highly fluctuating as a consequence, significant transient enhancements in dynamic pressure or plasma flux, above the general fluctuation level, can regularly be identified. These enhancements can reach values in dynamic pressure that are on the order of or even noticeably above the upstream solar wind values, although, usually, the dynamic pressure should be about an order of magnitude lower in the subsolar magnetosheath than upstream of the bow shock. This review paper deals with these enhancements in dynamic pressure, marked with dark blue arrows in Fig. 1, which we shall henceforth simply call: “jets”.

The term “jets” is just one out of many that have been used in the past to name similar phenomena in the magnetosheath. As diverse as the names are the definitions of “jets” in literature (see Sect. 2 for details). Arguably, Němeček et al. (1998) were first in reporting jets in the magnetosheath, calling them “transient flux enhancements”. The year of this publication, 1998, shows that this phenomenon has been subject of research for only 20 years. Within these 20 years, the diversity in approaches to this subject by different groups, independent from each other, has created a puzzle of unconnected reports and findings, based on different definitions of jets and data sets. This review aims at bringing the pieces together to form a complete picture of our current knowledge and understanding of the phenomenon of magnetosheath jets.

Jets are a key element to the coupling of the solar wind to the magnetosphere–ionosphere system. Their relevance stems from both, occurrence and impact. Large scale jets alone, with cross-sectional diameters of over $2\,R_{\mathrm{E}}$ (Earth radii), impact the dayside magnetopause every 6 to 7 minutes on average under low IMF cone angle conditions, i.e., downstream of the quasi-parallel bow shock (Plaschke et al. 2016). Conditions favorable for the occurrence of jets and their rates/locations of occurrence are discussed in Sect. 3 in more detail. A review on the properties of jets, including their scales sizes, is presented in Sect. 4.

A relative majority of jets (though not all of them) are associated with the quasi-parallel shock. Hence, a review on possible generation mechanisms of jets necessarily has to be accompanied with an introduction to foreshock structures, which can be found in Sect. 5. The most promising explanation for the generation of a larger part of the magnetosheath jets is based on the undulation or rippling of the quasi-parallel bow shock. While passing the inclined surfaces of a shock ripple, the solar wind should be less decelerated and thermalized, though still be compressed, explaining the excess in dynamic pressure with respect to the ambient magnetosheath plasma (e. g., Hietala et al. 2009). In this picture, the jets’ scale sizes and occurrence should be related to those of the bow shock ripples (Hietala and Plaschke 2013). As jets can easily propagate through the entire magnetosheath and reach the magnetopause, they naturally transmit and couple foreshock and bow shock structures across the entire magnetosheath region to processes at the magnetopause and beyond (Savin et al. 2012).

Once jets impact the magnetopause, they may lead to a number of consequences, as illustrated in Fig. 1 and detailed in Sect. 6: They produce indentations of the magnetopause, may launch surface waves on it and/or trigger local magnetopause reconnection. Inside the magnetosphere, compressional waves may be observed on jet impact, possibly affecting the radiation belt electron populations. Even on and from the ground, effects of jet impacts may be observable in form of ionospheric flow enhancements, magnetic field fluctuations, or throat auroral features. Hence, jets may contribute in multiple ways to terrestrial space weather, defined as environmental conditions in space that may have repercussions on human activities. As contributors to space weather and carriers of plasma, associated mass, momentum, and energy, jets bear some similarities to bursty bulk flows in the Earth’s magnetotail, which they are compared to in Sect. 7.

The occurrence of jets should not be restricted to the terrestrial magnetosheath. Instead, the phenomenon should be universal downstream of collisionless shocks. In Sect. 8, jets downstream of other planetary and astrophysical shocks are discussed. We conclude this review paper with an outlook Sect. 9, in which a number of open questions are posed and future challenges in jet research are stated.

## Definitions

**Fig. 2:**
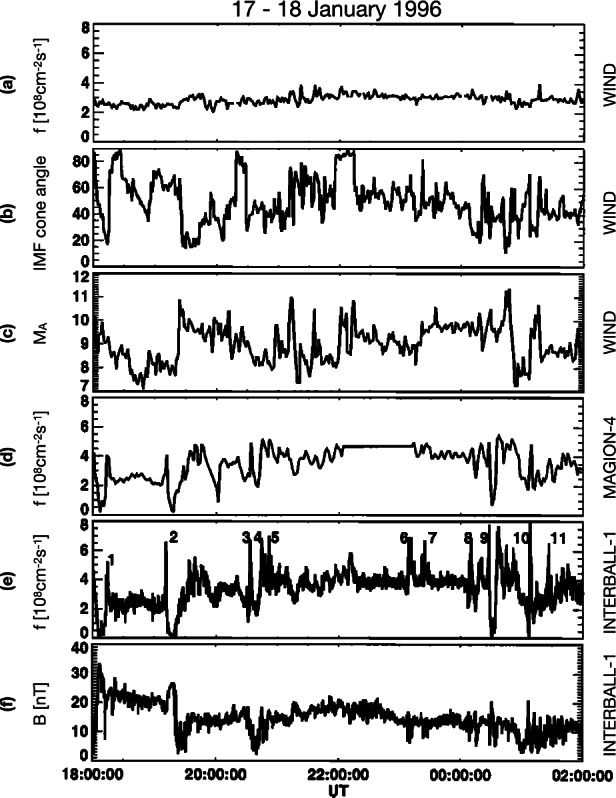
First observations of “transient flux enhancements” (TFE) by Interball-1, numbered in panel (e), along with upstream Wind observations. From top to bottom: solar wind ion flux, IMF cone angle, and Alfvén Mach number measured by Wind, magnetosheath ion flux measured by Magion 4 and Interball-1, and magnetic field strength measured by Interball-1. After Fig. 1 in Němeček et al. (1998)

**Fig. 3:**
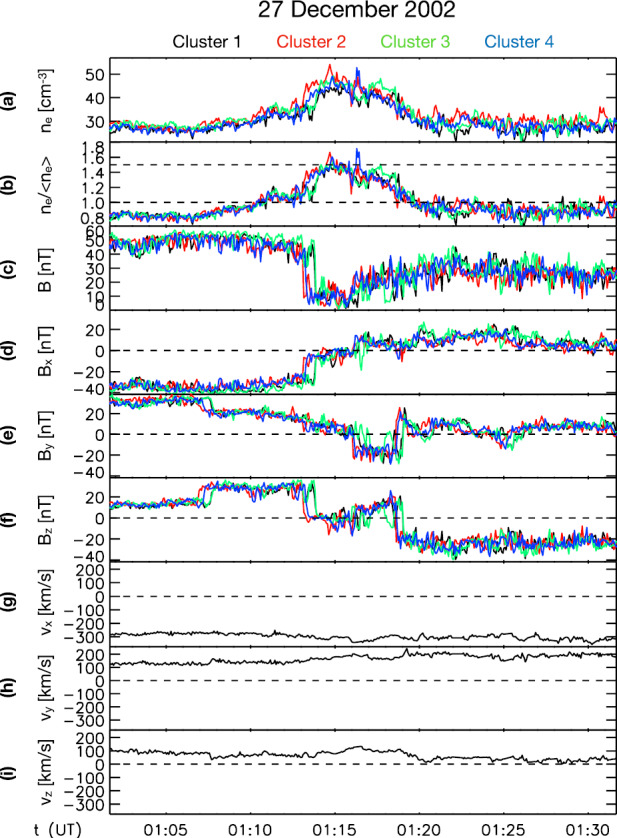
Example “plasmoid”, a structure with enhanced density, observed by the four Cluster spacecraft. From top to bottom: electron density, density ratio with respect to background, magnetic field and drift velocity in geocentric solar ecliptic (GSE) coordinates. After Fig. 3 in Karlsson et al. (2012)

**Fig. 4:**
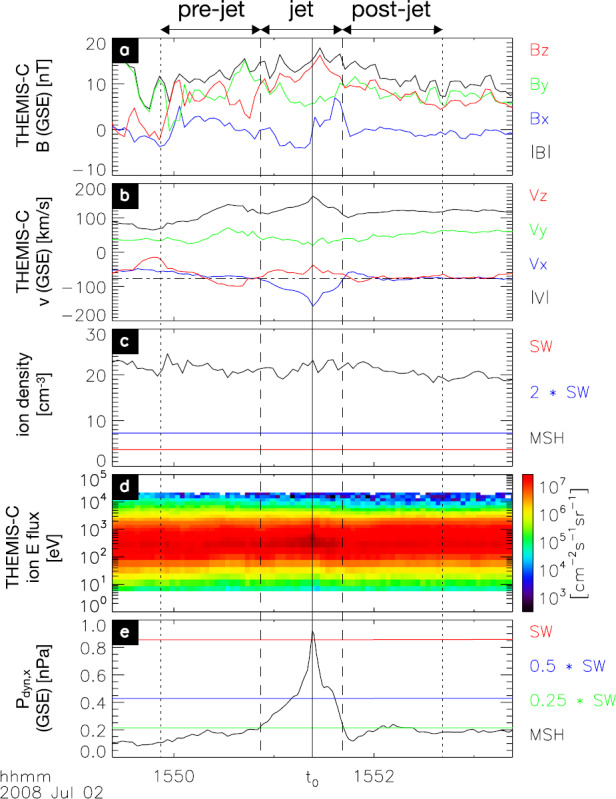
Example “high-speed jet”, an enhancement in the anti-sunward velocity and dynamic pressure based on the $x$ component of the ion velocity (in GSE). Magnetosheath (MSH) measurements by THEMIS-C, solar wind (SW) measurements from NASA’s OMNI data set. After Fig. 1 in Plaschke et al. (2013)

Since the first case study of Němeček et al. (1998), varying nomenclature has been used throughout the literature to describe transient enhancements in magnetosheath: ion flux $\rho _{\mathrm{msh}} v_{\mathrm{msh}}$ (see Fig. 2), mass density $\rho _{\mathrm{msh}}$ (see Fig. 3), bulk velocity $v_{\mathrm{msh}}$, full dynamic pressure $P_{\mathrm{dyn,msh}}=\rho _{\mathrm{msh}} v_{\mathrm{msh}}^{2}$, or dynamic pressure based on just one (e.g., $x$) component of the velocity $P_{\mathrm{dyn,msh,}x}=\rho _{\mathrm{msh}} v_{\mathrm{msh,}x}^{2}$ (see Fig. 4). Furthermore, such structures have been identified using various different criteria, including the physical quantity (or quantities) of interest and threshold value(s) chosen. The purpose of this section is thus to summarise the definitions which have been used to date and to ascertain whether they in fact concern different or indeed the same (or similar) phenomena. Table 1 displays key information about the definitions of these transients in the literature for studies concerning more than one transient where identification criteria were explicitly stated.

**Table 1:** Summary of definitions

Paper	Name of transient	Date(s)	Spacecraft	Location	Physical quantity	Threshold	Number	Notes
Němeček et al. (1998)	Transient Flux Enhancement	18:00–02:00 UT 17 Jan 1996	Interball-1 Magion-4	Dusk Flank	$\rho _{\mathrm{msh}} v_{\mathrm{msh}}$	$\frac{3}{2} \langle \rho _{\mathrm{msh}} v_{\mathrm{msh}} \rangle _{20\,\mathrm{min}}$	11	
Savin et al. (2008)	High Kinetic Energy Density Plasma Jet	09:00–11:00 UT 27 Mar 2002	Cluster	Southern Cusp	$\frac{1}{2}\rho _{\mathrm{msh}} v_{\mathrm{msh}}^{2}$	$\mu +\frac{3}{2}\sigma $	83	Normal distribution parameters fitted to main population ignoring tail
Amata et al. (2011)	High Kinetic Energy Jet	09:10–12:10 UT 17 Mar 2001	Cluster	Northern Cusp	$\frac{1}{2}\rho _{\mathrm{msh}} v_{\mathrm{msh}}^{2}$	$\frac{1}{2}\rho _{\mathrm{sw}} v_{\mathrm{sw}}^{2}$	8	
Karlsson et al. (2012, 2015b)	Plasmoid	13 Dec 2002–6 May 2003 1 Feb 2005–13 Apr 2005 14 Dec 2005–14 Jun 2006	Cluster	Dayside	$\rho _{\mathrm{msh}}$	$\frac{3}{2} \langle \rho _{\mathrm{msh}} \rangle _{500\,\mathrm{s}}$	56	$\begin{cases} v_{\mathrm{msh}} \geq 1.1 \langle v_{\mathrm{msh}} \rangle \\ \quad \text{Fast}\\ v_{\mathrm{msh}} <1.1 \langle v_{\mathrm{msh}} \rangle \\ \quad \text{Embedded} \end{cases}$
Hietala et al. (2012)	Supermagnetosonic Jets	17:00–20:30 UT 17 Mar 2007	Cluster	Dayside	$\begin{array}{c} v_{\mathrm{msh}}\\ \rho _{\mathrm{msh}} v_{\mathrm{msh}}^{2} \end{array}$	$\begin{array}{c} v_{\mathrm{ms},\mathrm{msh}}\\ \rho _{\mathrm{sw}} v_{\mathrm{sw}}^{2} \end{array}$		
Archer et al. (2012)	Dynamic Pressure Pulse	15:00–20:00 UT 30 Sep 2008	THEMIS	Dayside	$\rho _{\mathrm{msh}} v_{\mathrm{msh}}^{2}$	∼1.5 nPa	14	Background ∼0.5 nPa
Archer and Horbury (2013)	Dynamic Pressure Enhancement	Jun 2008–Sep 2008	THEMIS	Dayside	$\rho _{\mathrm{msh}} v_{\mathrm{msh}}^{2}$	$2 \langle \rho _{\mathrm{msh}} v_{\mathrm{msh}}^{2} \rangle _{20\,\mathrm{min}}$	2617	
Plaschke et al. (2013)	High Speed Jet	2008–2011	THEMIS	Subsolar ($\theta _{\mathrm{s}} <30^{\circ}$)	$\begin{array}{c} \rho _{\mathrm{msh}} v_{\mathrm{msh},x}^{2}\\ v_{\mathrm{msh},x} < 0 \end{array}$	$\begin{array}{c} \frac{1}{2}\rho _{\mathrm{sw}} v_{\mathrm{sw}}^{2}\\ 2 \langle v_{\mathrm{msh},x} \rangle \end{array}$	2859	
Savin et al. (2014)	Supermagnetosonic Plasma Stream	00:00–20:00 UT 27 Mar 2005	Cluster Doublestar	Dayside	$\begin{array}{c} \rho _{\mathrm{msh}} v_{\mathrm{msh}}^{2}\\ \rho _{\mathrm{msh}} v_{\mathrm{msh}}^{2} / \rho _{\mathrm{sw}} v_{\mathrm{sw}}^{2} \end{array}$	*μ* + 3*σ*		
Gunell et al. (2014)	Plasmoid	Mar 2007	Cluster	Northern Cusp	$v_{\mathrm{msh},x}<0$	$2 \langle v_{\mathrm{msh,}x} \rangle $	65	
Dmitriev and Suvorova (2015)	Large Scale Jet	2007–2009	THEMIS	Dayside	$\rho _{\mathrm{msh}} v_{\mathrm{msh}}^{2} + P_{\mathrm{th,msh}} + P_{B,\mathrm{msh}}$	$\rho _{\mathrm{sw}} v_{\mathrm{sw}}^{2} + P_{\mathrm{th,sw}} + P_{B,\mathrm{sw}}$	646	${>}30~\mathrm{s}$ duration directed to magnetopause
Gutynska et al. (2015)	Density Enhancements	Jun 2007–Dec 2008	THEMIS	Dayside & Flanks	$\rho _{\mathrm{msh}}$	$\frac{3}{2} \langle \rho _{\mathrm{msh}} \rangle _{500\,\mathrm{s}}$	1312	

### Physical Quantities and Nomenclature

A number of different physical quantities have been used to identify transient events. While originally Němeček et al. (1998) looked at ion flux $\rho _{\mathrm{msh}} v_{\mathrm{msh}}$ due to the limitations of Interball-1 and Magion 4’s plasma instrumentation, Table 1 demonstrates that most studies since have used the magnetosheath dynamic pressure (in Earth’s rest frame) $P_{\mathrm{dyn,msh}} = \rho _{\mathrm{msh}} v_{\mathrm{msh}}^{2}$. Other studies have also used quantities either proportional to this, such as the kinetic energy density $\frac{1}{2} \rho _{\mathrm{msh}} v_{\mathrm{msh}}^{2}$, or quantities which are typically dominated by the dynamic pressure in these structures, e.g., the sum of dynamic, thermal ($P_{\mathrm{th,msh}}$) and magnetic ($P_{B,\mathrm{msh}}$) pressures (see Sect. 4 for more on properties). The use of dynamic pressure is perhaps a natural choice when considering possible magnetospheric effects (discussed in Sect. 6), since the magnetopause’s location and motion are largely controlled by the balance of pressure on either side of the boundary. However, a number of studies have investigated enhancements in the density (Karlsson et al. 2012, 2015b; Gutynska et al. 2015) or in the (anti-sunward/negative-$x$ component of the) velocity (Hietala et al. 2009, 2012; Gunell et al. 2014).

The choice of physical quantity has often affected the nomenclature of these transients. A number of studies have simply labelled the transients as “enhancements” or “pulses” in said physical quantity, however the most common term to date has been as “jets” of some description. A similar term “plasma stream” was used by Savin et al. (2014). It should be noted that some “jets” may not necessarily have an enhancement in the magnetosheath flow velocity, though such cases are in the minority (e.g. Archer and Horbury 2013) as discussed more thoroughly in Sect. 4.2. However, some definitions of a fluid jet consider an increase in momentum, and not necessarily only velocity (e.g. Prokhorov 1974). Finally, the name “plasmoid” has also been applied citing its use in describing either a “plasma-magnetic entity” (Bostick 1956) or “a coherent mass of plasma” (Simpson and Weiner 1989). This has typically been ascribed to enhancements in magnetosheath density (Karlsson et al. 2012, 2015b), though curiously Gunell et al. (2014) used the term to describe velocity structures. Gunell et al. (2014) discussed how the nomenclature of the transient structures may implicitly evoke their spatial structure. They argued “pulses” should have large cross-sectional areas comparable to that of the magnetosphere, “jets” or “streams” imply an elongation along the flow across the entire magnetosheath depth but with small transverse scales, whereas “plasmoids” best describe structures with small dimensions in all directions compared to the magnetosheath system in which they occur. As discussed in Sects. 4.3 and 9.2, while some results on the morphology of these structures have been reported, this topic remains a current area of investigation.

In addition to the terms above, the naming of these transients has often included some qualifying statements. Hietala et al. (2009, 2012) and Savin et al. (2014) highlight in their respective nomenclature the supermagnetosonic (being faster than the local magnetosonic wave speed $v_{\mathrm{ms,msh}}$) nature of the flows, uncommon for shocked plasma in the dayside magnetosheath. However, the identification criterion of Savin et al. (2014) does not strictly depend on the magnetosonic Mach number so in this case the transients being labelled as supermagnetosonic is technically something of a misnomer. Dmitriev and Suvorova (2015) use the term “large-scale” when discussing their jets. It is not explicitly stated what this means, though perhaps it is related to their required minimum $30\,\mathrm{s}$ duration. Finally, Karlsson et al. (2012, 2015b) subdivide their plasmoids into “fast” and “embedded” categories based on the velocity within the density enhancements compared with the background value.

### Imposed Thresholds

The identification of a transient enhancement in a quantity requires imposing some threshold value. In terms of the transients in discussion, two general approaches have been used based on either the upstream conditions (e.g., Amata et al. 2011; Plaschke et al. 2013) in the pristine solar wind or the local background magnetosheath conditions (e.g., Archer and Horbury 2013; Karlsson et al. 2012, 2015b). We use angular brackets to denote local background values, which are often determined by a running average whereby the timescale is displayed in subscript.

Both methods have their advantages and disadvantages. As discussed by Plaschke et al. (2013), the use of a running average to determine the local background limits the timescales of the transients which one can identify—a limitation not present when comparing to solar wind conditions. The averaging timescales used have varied from ${\sim} 8$ to $20~\mbox{min}$, much larger than the duration of the majority of transients and also greater than their typical recurrence timescale (Archer and Horbury 2013; Plaschke et al. 2013). However, another important point is that running averages may not give representative background values close to moving boundaries such as the bow shock and magnetopause. Solar wind conditions, on the other hand, are typically measured far upstream of Earth’s bow shock at the L1 Lagrangian point. This means the data must be time-lagged to correlate with the corresponding magnetosheath observations. Various methods of performing this lagging procedure have been developed and while they generally provide good estimates of the conditions close to Earth, errors in the lags can be up to ${\sim} 30~\mbox{min}$ (Mailyan et al. 2008; Case and Wild 2012) and sometimes features observed by solar wind monitors are different or simply not present near Earth and vice versa (e.g., Šafránková et al. 2009). Furthermore, since both the average density and velocity relative to those in the solar wind vary with position in the magnetosheath (e.g., Dimmock and Nykyri 2013), thresholds using upstream conditions can only be applied to certain regions of the magnetosheath. For example, the criteria of Plaschke et al. (2013) that $\rho _{\mathrm{msh}} v_{\mathrm{msh},x}^{2} \geq \frac{1}{2} \rho _{\mathrm{sw}} v_{\mathrm{sw}}^{2}$ may only be applied to the subsolar region, since in the flanks the magnetosheath velocity with respect to the solar wind increases such that this inequality would be satisfied almost all the time.

Whether upstream or local conditions are used, the minimum size of the increase must be suitably large to prevent false detections. For example, the downstream signatures of step changes in the solar wind dynamic pressure could erroneously be selected using these criteria if less than a 50% increase were required based on a local running-average method or, in the case of an upstream monitor, if there was an error in the time-lag and the change was a significant fraction of the solar wind dynamic pressure. From Table 1 it appears that the thresholds used have been sufficiently large to mitigate such false detections. Unlike most of the thresholds used to date which have been somewhat arbitrarily set, Savin et al. (2008) chose their minimum kinetic energy density based on the distribution of the magnetosheath data. They noted that this distribution resembled a Gaussian with a (possibly exponential) tail and therefore set their threshold for the jets as the approximate location of the break between Gaussian and non-Gaussian behaviour. By performing a fit to the main population, they showed that this break corresponded to about one-and-a-half standard deviations $(\sigma )$ above the mean $(\mu )$.

### Comparison

**Fig. 5:**
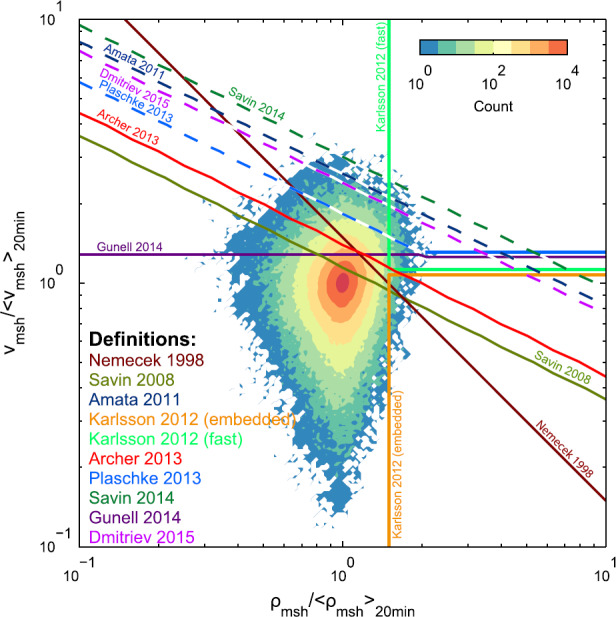
The joint distribution of magnetosheath density and velocity normalised by their respective local background values (given by a running average). The various thresholds used to define transients are indicated by the coloured lines (see Table 1). Dashed lines mean that the criterion used was based on upstream conditions, thus the depicted threshold is only approximate

While Table 1 lists the various identification criteria that have been used, it is helpful to visualise all of the quantities and associated thresholds used and thereby make some assessment of their relative occurrences and the amount of overlap between them. Figure 5 displays the joint probability distribution of the magnetosheath density (horizontal axis) and velocity (vertical axis) divided by their respective $20\,\mathrm{min}$ running averages from the Archer and Horbury (2013) Time History of Events and Macroscale Interactions during Substorms (THEMIS) magnetosheath survey limited to solar zenith angles $\theta _{\mathrm{s}} < 30^{\circ }$. On the logarithmic scales used, enhancements in density thus correspond to the right hand side of the figure and increases in velocity occupy the upper half. The contours show that the spread in magnetosheath velocities is greater than the spread in densities with no overall correlation between the two quantities. The various coloured lines depict the thresholds listed in Table 1. Since the datapoints in Fig. 5 are normalised by the nominal background values, only criteria which use local conditions to identify transients are strictly valid in this parameter space and are depicted by the solid lines. In the case of criteria which use upstream conditions, we have approximated the thresholds within the parameter space depicted by using the average ratio of local background to upstream conditions over the survey and, where applicable, assuming that enhanced velocities are directed along the Sun–Earth line (c.f., Archer and Horbury 2013). These are shown as the dashed lines.

The vertical green lines correspond to the Karlsson et al. (2012, 2015b) “plasmoid” criterion, where we have separated the “fast” and “embedded” events. Gutynska et al. (2015) later used the same criterion but without this latter distinction. The velocity criterion of Gunell et al. (2014) can be seen as the purple horizontal line, indicating that fast plasma flows with either enhanced, similar or reduced density are all identified. The original ion-flux criterion of Němeček et al. (1998) can be seen as the single steep diagonal line. The series of shallower parallel diagonal lines correspond to the criteria which use magnetosheath dynamic pressure. Each line constitutes a certain proportional change in this quantity. It is clear that this threshold is lowest for Savin et al. (2008), which again was based on the approximate start position of the non-Gaussian tail, while the one of Savin et al. (2014) was the highest. For the two criteria which have identified the most transients using dynamic pressure, Archer and Horbury (2013) and Plaschke et al. (2013), the former has the lower threshold and hence should identify more events. Note that the additional velocity criterion of Plaschke et al. (2013) (the blue horizontal line) does not appear to omit many data points than if it were ignored (i.e. continuing the blue diagonal). Thus according to Fig. 5, Plaschke et al. (2013) identified “high-speed jets” should be a subset of the Archer and Horbury (2013) “dynamic pressure enhancements”. However, due to the previously mentioned assumptions used to incorporate criteria based on upstream conditions, such an assessment is not strictly true.

**Fig. 6:**
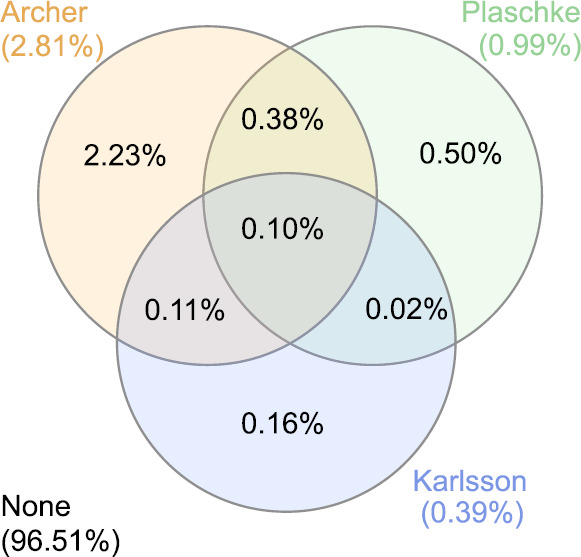
Venn diagram showing the percentages of the time in the subsolar ($\theta _{\mathrm{s}} < 30^{\circ }$) magnetosheath from the Archer and Horbury (2013) survey that the three most used definitions of transients occur and the amount of overlap

To address this issue, we investigate the *actual* overlap in the three definitions which have identified the most events: Karlsson et al. (2012, 2015b) later used by Gutynska et al. (2015), Archer and Horbury (2013) and Plaschke et al. (2013). The definitions were applied to the Archer and Horbury (2013) survey for $\theta _{\mathrm{s}} < 30^{\circ }$ with no further assumptions this time and a Venn diagram was constructed (Fig. 6) where the percentages correspond to the amount of time throughout the entire survey. For the vast majority of the time in the magnetosheath (96.51%) none of the three critera are satisfied, which is expected since jets constitute extreme events. Just under 3% of all identified data points are common to all three definitions, corresponding to 0.1% of the entire magnetosheath survey. In agreement with Fig. 5, Archer and Horbury (2013) identifies the most points and Karlsson et al. (2012) the least. About 60% of all observations identified using the Karlsson et al. (2012) criterion also satisfy one of the other two definitions, though there are barely any of these which do not satisfy Archer and Horbury (2013). Those data points identified by only Karlsson et al. (2012) likely correspond to “embedded plasmoids” with between 50 and 100% increases in density or “fast plasmoids” with less than 65% density increases, since neither of these would be picked up by Archer and Horbury (2013). Somewhat surprisingly, about half of the Plaschke et al. (2013) samples are not also satisfied by the other two definitions. Further investigation is required to understand this. One possible explanation may be due to the use of thresholds using upstream compared to local conditions and how both methods vary with position, even when limiting the survey to the subsolar magnetosheath.

### Conclusions

By collectively looking at the definitions of transient density and/or velocity increases within Earth’s magnetosheath, a number of commonalities have been identified amongst the various previous studies. They are mostly called “jets”, which is the terminology we use throughout this review, and are typically identified using dynamic pressure in Earth’s rest frame. Either local background or upstream conditions are used to set the threshold value and the precise values used have varied from study to study, therefore some criteria identify more events than others. In this paper, we would like to refrain from recommending the use of a specific set of jet identification criteria for future studies, as the particular science questions to be addressed and the availability or absence of certain measurements may require the use of different criteria.

Interestingly, although the overlap between the various definitions used is relatively small (as of Fig. 6), there is a large amount of agreement in the structures’ occurrence (see Sect. 3) and properties (see Sect. 4). Consequently, it is highly likely that the different criteria concern very similar, if not exactly the same, phenomena.

## Occurrence

The purpose of this chapter is to address three major questions with respect to the occurrence of magnetosheath jets:

1. Where in the magnetosheath are jets observed?
2. Under which solar wind conditions do jets occur?
3. How often do they occur?

The answers to these three questions are dependent on the particular definition of jets used and on the generation/source mechanisms from which they originate. We will first briefly summarize the findings of case (and multi-case) studies in the next Sect. 3.1. These studies served as the motivation for larger statistical investigations reviewed in the following Sect. 3.2.

### Case Studies on Jet Occurrence

Early observations of transient flux enhancements reported by Němeček et al. (1998) occurred on the flank magnetosheath during intervals of steady solar wind with relatively high Alfvénic Mach number $(M_{\mathrm{A}} > 7)$. The structures were seen on stream lines linked to the quasi-parallel bow shock. These observations are consistent with later results by Němeček et al. (2000) who analyzed magnetosheath fluctuations in ion fluxes with respect to the solar wind. Transient flux enhancements obviously constitute a subset of this overall variability. They found that low IMF cone angles ($15^{\circ }$ to $30^{\circ }$ between the IMF and the Sun–Earth line) had much greater fluctuation levels with respect to those expected simply by transmission of solar wind variations (see also, Němeček et al. 2002; Zastenker et al. 2002; Shevyrev et al. 2003; Karimabadi et al. 2014). The quasi-radial IMF configuration is not rare. Suvorova et al. (2010) reported that cone angles below $30^{\circ }$ are seen about $16\%$ of the time. Radial IMF is particularly common at the trailing edges of magnetic clouds resulting from coronal mass ejections: Neugebauer et al. (1997) reported that approximately $1/5$ of these events feature long periods of steady radial IMF conditions.

Jet observations taking place downstream of the quasi-parallel bow shock under relatively quiet solar wind conditions have indeed been reported quite often. Savin et al. (2008), for instance, reported such observations by Interball-1 near the northern cusp, close to the magnetopause, and by Cluster near the southern cusp, closer to the bow shock. Hietala et al. (2009) and Hietala et al. (2012) also reported Cluster observations of jets downstream of the quasi-parallel shock. They proposed that a bow shock, rippled due to foreshock effects, would produce plasma jets of less thermalized, less decelerated plasma within the magnetosheath (see Sect. 5.2.2). When the magnetic field and flow velocity in the solar wind are aligned (low IMF cone angles) and $M_{\mathrm{A}}$ is high, then the downstream effects of bow shock ripples should be more pronounced, implicitly restricting the most intense effects to the day side, subsolar magnetosheath. Note that at the flanks of the magnetosheath the plasma stream is already super-magnetosonic and so bow shock ripples at the flanks may not necessarily create discernible jets (depending on the definition used to identify jets, see Sect. 2), but rather contribute to the overall downstream variability.

That jets can penetrate the magnetosheath all the way to the magnetopause is evidenced, e.g., by results from Shue et al. (2009). Magnetospheric consequences of jets are described in detail in Sect. 6. Numerical simulation results by Hao et al. (2016a) show that jets associated with the high solar wind Alfvén Mach numbers ($M_{\mathrm{A}}$) are able to penetrate further into the magnetosheath and impact the magnetopause more easily than under lower $M_{\mathrm{A}}$ conditions. However, jets may form also under these low $M_{\mathrm{A}}$ conditions.

Quite a range of jet recurrence times have been reported. Archer et al. (2012) investigated jets that were related to discontinuities in the IMF. For the specific interval investigated, they saw the time scales of jet recurrence to be between 3 to $5~\mbox{min}$, though there were also large time periods without any pulses. Savin et al. (2011) reported an interval of jets with recurrence times of 6 to $7~\mbox{min}$. The authors associated these jets with hot flow anomalies (see Sect. 5.3), which interestingly have been found to occur at much lower rates of a few per day (Schwartz et al. 2000; Turner et al. 2013; Chu et al. 2017).

### Statistical Studies on Jet Occurrence

A few years ago, two large and comprehensive statistical studies on jets in the magnetosheath were published (Archer and Horbury 2013; Plaschke et al. 2013) that shed more light on the favorable conditions for the occurrence of jets. In agreement therewith are findings by Gutynska et al. (2015), who performed an automated search for transient density enhancements in THEMIS magnetosheath data.

Archer and Horbury (2013) looked for dynamic pressure enhancements in the magnetosheath over a $20~\mbox{min}$ running average, taking into account all the components of the ion velocity (see Table 1 in Sect. 2). Jets were observed all over the equatorial, dayside magnetosheath, up to (and slightly beyond) the dawn and dusk terminators. No strong dawn-dusk asymmetry was apparent. Archer and Horbury (2013) assigned each location of jet observation a fractional/relative distance $F$ between the magnetopause ($F=0$) and bow shock ($F=1$), as well as an aberrated solar zenith angle $\theta _{\mathrm{s}}$, that is negative/positive for the dawn/dusk magnetosheath. Furthermore, they used a stream line model to link each of those locations to a point on the bow shock, so that they could obtain a corresponding angle between the upstream IMF and the local bow shock normal ($\theta _{Bn}$) to ascertain if a particular jet was associated with the quasi-perpendicular or quasi-parallel shock.

The main selection criterion for jets in Plaschke et al. (2013) was, instead, based on the dynamic pressure in the (negative) geocentric solar ecliptic (GSE) $x$-direction only ($\rho _{\mathrm{msh}} v_{\mathrm{msh,}x}^{2}$) with a threshold of half the upstream solar wind value (see Table 1 in Sect. 2). Hence, jets were identified with respect to the solar wind conditions and not in comparison with the background ambient conditions in the magnetosheath.

**Fig. 7:**
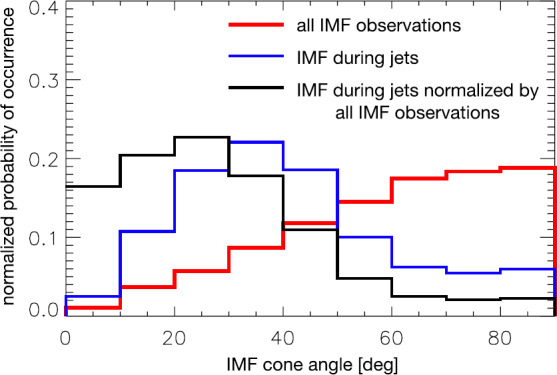
Jet occurrence in the subsolar magnetosheath versus IMF cone angle (blue), normalized distribution (black) against overall occurrence of IMF cone angles (red). After Fig. 8 in Plaschke et al. (2013)

Both statistical studies reached the conclusion that jets are predominantly seen downstream of the quasi-parallel shock, consistent with a number of case studies. Generally, the IMF is steadier than usual, when jets are observed. Hence, the majority of jets are not associated with IMF discontinuities but with stable foreshock structures or processes. Correspondingly, Archer and Horbury (2013) found $\theta _{Bn}$ to be the main parameter controlling occurrence (low $\theta _{Bn}$ favorable for jet occurrence). Plaschke et al. (2013) likewise found the IMF cone angle (similar to $\theta _{Bn}$ with respect to the subsolar shock and magnetosheath) to be the only occurrence controlling parameter (see Fig. 7). Apart from that, jet occurrence is found to be only weakly dependent on other averaged upstream conditions, if at all: There is no (clear) dependence on IMF clock angle, IMF strength, solar wind plasma beta or Mach number. However, solar wind dynamic pressures and velocities tend to be slightly enhanced during jet occurrence times, while solar wind densities tend to be slightly lower (see left panels of Fig. 13).

**Fig. 8:**
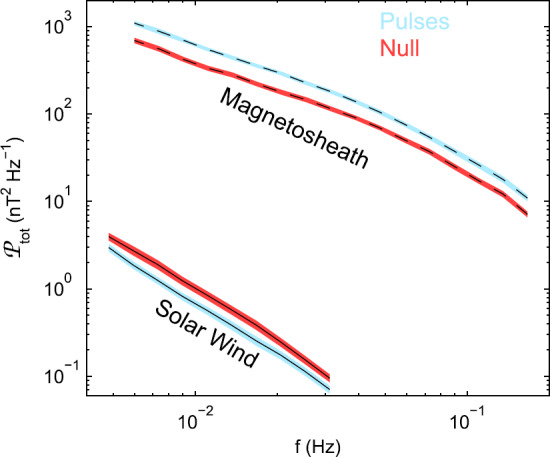
Mean background superposed wavelet power spectra in the magnetosheath (dashed) and solar wind (solid) for THEMIS dynamic pressure enhancements (blue) and null (red) events. After Fig. 7 in Archer and Horbury (2013)

The variability in IMF direction, IMF strength, solar wind density and velocity does not seem to have a large influence on jet occurrence, either: Plaschke et al. (2013) calculated the maximum deviations between any two entries for a number of solar wind quantities in the NASA OMNI solar wind data (King and Papitashvili 2005) within the $5~\mbox{min}$ intervals preceding jets. Distributions of these deviations were not different from general solar wind intervals. Archer and Horbury (2013) used super-posed epoch analysis of the wavelet power in the solar wind magnetic field corresponding to magnetosheath dynamic pressure pulses. Thereof they determined that the majority of the jets did not show increased wavelet power in the IMF, and that the solar wind mean background total wavelet power was typically smaller during jet events than during null events (see Fig. 8): the IMF was in fact steadier than usual. Hence both studies point to the importance of a stable foreshock region. These results do not contradict the fact that changes in shock character, in particular from quasi-perpendicular to quasi-parallel, can be associated with (large amplitude) jets (e.g., Archer et al. 2012). These cases, however, only seem to constitute a smaller subset of all jets observed in the magnetosheath.

**Fig. 9:**
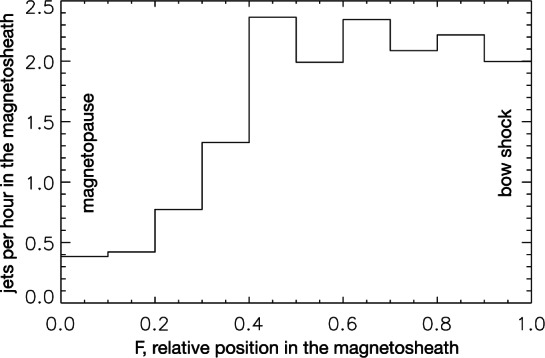
Distribution of jet observations between the magnetopause (relative position $F=0$) and the bow shock (relative position $F=1$) normalized by single-spacecraft dwell times of the observing THEMIS spacecraft in the subsolar magnetosheath. After Fig. 3 in Plaschke et al. (2013)

**Fig. 10:**
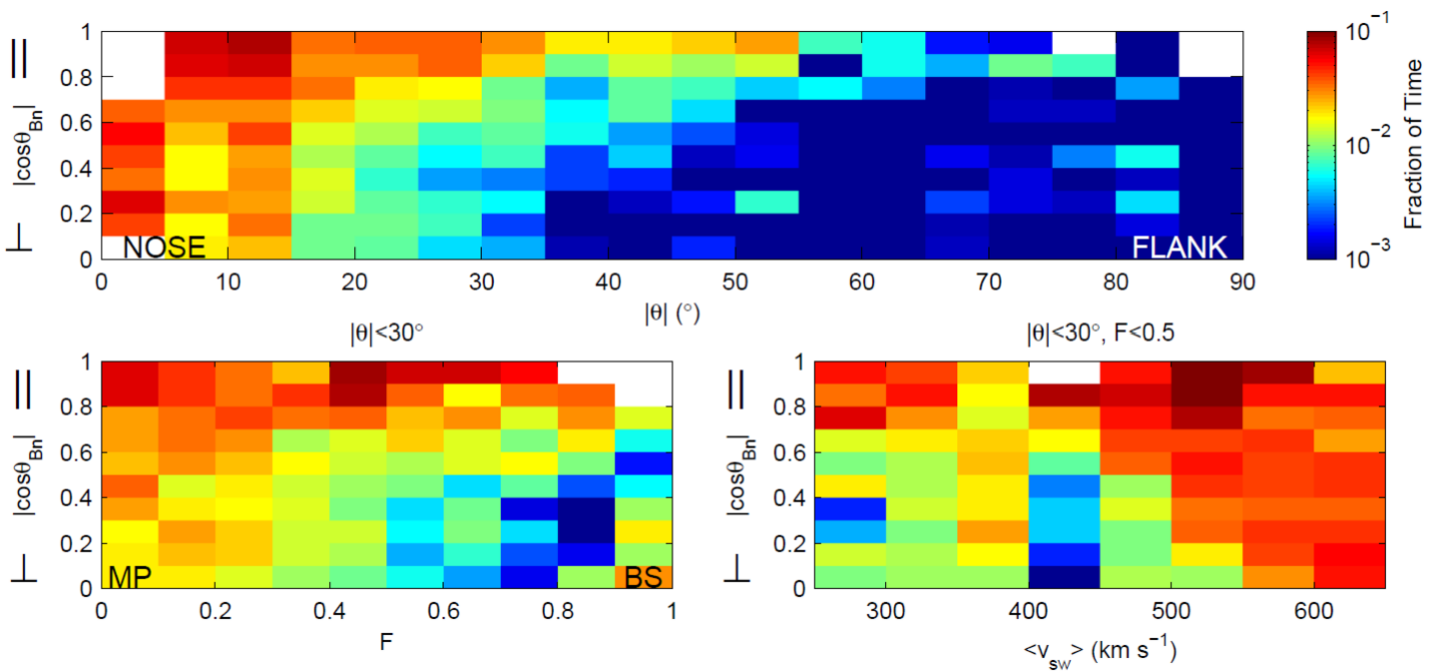
Normalized distributions of jet observations as a function of shock character ($\theta _{Bn}$) versus solar zenith angle $\theta _{\mathrm{s}}$, $F$, and solar wind velocity. Figure 2 in Archer and Horbury (2013)

Interestingly, Plaschke et al. (2013) and Archer and Horbury (2013) differ with respect to the occurrence as a function of location in the magnetosheath, likely due to their different definitions (see Sect. 2). The former find that jets are much more frequent closer to the bow shock than to the magnetopause, when normalizing with the amount of magnetosheath observations as a function of $F$ (see Fig. 9). Archer and Horbury (2013) report that there is no obvious trend in the occurrence with $F$ of jets downstream of the quasi-parallel shock, as can be seen in Fig. 10, which also applies to the flanks (regions not studied by Plaschke et al. 2013). Downstream of the quasi-perpendicular shock, occurrence even increases toward the magnetopause ($F < 0.5$), in particular in the subsolar sector. This may mean that these particular jets are rather associated with the magnetopause (e.g., via reconnection or the presence of a plasma depletion layer). Toward the flanks, jets (as defined by Archer and Horbury 2013) become less common, but that may also be due to the generally higher flow velocity of the background magnetosheath plasma. In contrast, Karlsson et al. (2015b) observe density enhancements throughout the dayside flanks.

Plaschke et al. (2013) find typical recurrence times of a few minutes between two jet observations while a single spacecraft stays within the magnetosheath. However, the distribution of recurrence times is very broad and spans over three orders of magnitude, with inter-jet times ranging from 6 to $8765~\mbox{s}$ (median: $140~\mbox{s}$). Hence, distinct times of recurrence are not apparent, which is also supported by simulation results by Hao et al. (2016a). They state that the jet “occurrence time along the shock front is mainly random without any apparent link with local self-reformation time”. Hietala and Plaschke (2013), however, find that the distribution of dynamic pressures in the magnetosheath (close to the bow shock under low IMF cone angle conditions) can match the expected flow coming from a rippled bow shock. Ripples would then need to feature an average amplitude to wave length ratio of about $9\%$ and be present about $12\%$ of the time at any point of the shock. Jets, therefore, comprise a significant fraction of the tail of the dynamic pressure distribution.

Archer and Horbury (2013) report that jets constitute $2\%$ of their single spacecraft magnetosheath observations data set. With lower $\theta _{Bn}$, their observational occurrence increases to $3\%$ (see Fig. 6) behind the quasi-parallel bow shock (and $10\%$ when limiting to the subsolar region, $\theta _{\mathrm{s}} < 30^{\circ }$), and decreases to $0.5\%$ downstream of the highly quasi-perpendicular shock.

In the Plaschke et al. (2013) dataset, the observation rate is $Q_{\mathrm{obs}}=0.89$ jets per hour of magnetosheath observations near the magnetopause, and three times higher ($Q_{\mathrm{obs},<30^{\circ }}=2.90/\mathrm{h}$) under low IMF cone angle conditions (${<}30^{\circ }$) in the subsolar magnetosheath (see Plaschke et al. 2016). These observation rates are not easily comparable to the results of Archer and Horbury (2013) mentioned above, because they give answers to different questions: which fraction of magnetosheath observation time belongs to jets (Archer and Horbury 2013) versus how many jets are observed per observation time (Plaschke et al. 2013, 2016).

It should also be noted that these rates heavily depend on the thresholds (e.g., on dynamic pressure) used for the identification of jets. Furthermore, even under favorable conditions, a spacecraft may not see any jets over longer magnetosheath intervals, while another spacecraft simultaneously present in the magnetosheath may do so (Plaschke et al. 2013). The reason is the limited spatial extent of (many of) the jets, that needs to be taken into account when computing a true occurrence rate of jets in contrast to a single spacecraft jet observation rate.

**Fig. 11:**
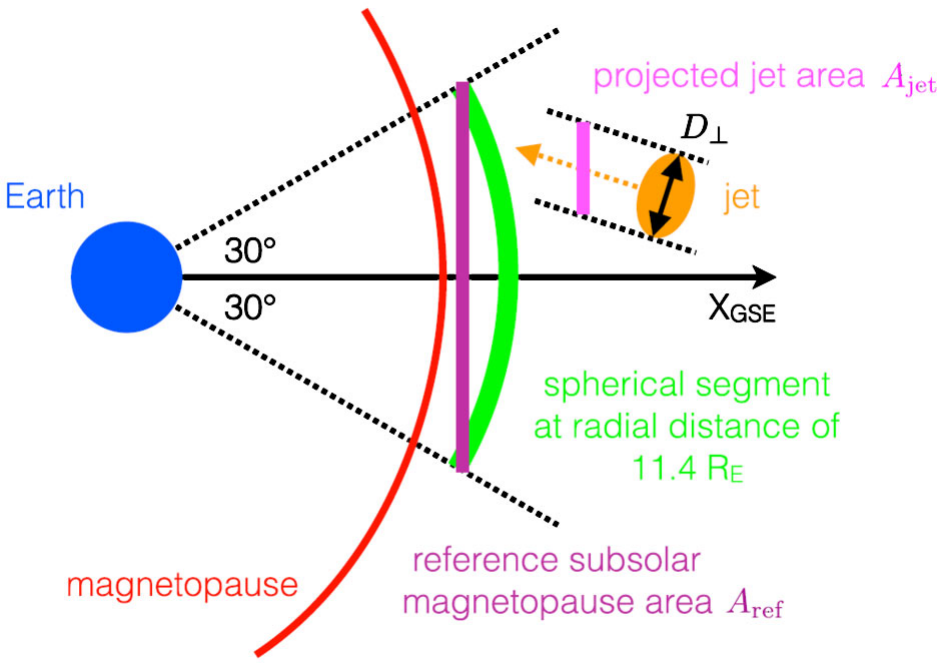
Illustration of the reference area and the projected jet cross-sectional area. Figure 6 in Plaschke et al. (2016)

This fact has been considered in detail by Plaschke et al. (2016) who calculated the impact rate of jets of a certain minimum size (cross-sectional diameter larger than $D_{\perp ,\mathrm{min}}$) on a reference area $A_{\mathrm{ref}} \sim 100\,R_{\mathrm{E}}^{2}$ covering the dayside subsolar magnetopause, as illustrated in Fig. 11. The impact rate is given by

1 $$ Q_{\mathrm{imp}} = \int _{D_{\perp ,\mathrm{min}}}^{\infty }C \, P_{\perp }\, Q_{\mathrm{obs}} \, \mathrm{d}D_{\perp } $$

where $P_{\perp }$ is the probability of occurrence of jets with respect to their cross-sectional diameter $D_{\perp }$, $Q_{\mathrm{obs}}$ is the single spacecraft observation rate of jets near the magnetopause, stated above, and $C=A_{\mathrm{ref}} / A_{\mathrm{jet}}$ is the ratio of the reference area and the cross-sectional area of the jets projected onto the reference area. The jet area is given by $A_{\mathrm{jet}} = \pi D_{\perp }^{2} / (4 \cos \theta )$, where $\theta =25^{\circ }$ is the average deviation of the jet propagation direction from the GSE $x$-direction (see Fig. 11), based on jet observations near the subsolar magnetopause in the Plaschke et al. (2013) data set.

In order to obtain $P_{\perp }$, Plaschke et al. (2016) considered how often jets were observed simultaneously by two spacecraft in a plane perpendicular to the jet propagation direction as a function of the inter-spacecraft distance. The fractions of simultaneous observations by two spacecraft could be successfully modeled by assuming $P_{\perp }$ to be an exponential distribution:

2 $$ P_{\perp }= \exp (-D_{\perp }/ D_{\perp 0}) / D_{\perp 0} $$

with $D_{\perp 0}=1.34\,R_{\mathrm{E}}$. Indeed, scale sizes of $1\,R_{\mathrm{E}}$ seem to be typical for jets, also found in other studies (e.g., Karlsson et al. 2012). Using $Q_{\mathrm{obs}}$ and $Q_{\mathrm{obs},<30^{\circ }}$, Plaschke et al. (2016) obtain impact rates of jets with cross-sectional diameter larger than $D_{\perp ,\mathrm{min}} = 2\,R_{\mathrm{E}}$ on the subsolar magnetopause of $Q_{\mathrm{imp}} = 2.9/\mathrm{h}$ in general and $Q_{\mathrm{imp},<30^{\circ }} = 9.4/\mathrm{h}$ under low IMF cone angle conditions. That is about once every $20~\mbox{min}$ and once every $6~\mbox{min}$ under favorable conditions. Note that jets with diameters over $D_{\perp ,\mathrm{min}} = 2\,R_{\mathrm{E}}$ are denoted as geoeffective in Plaschke et al. (2016).

The impact rates can be compared to occurrence rates of other transients. According to THEMIS statistics, hot flow anomalies (HFAs) occur about once every 2 hours and foreshock bubbles (FBs) only about once per day under favorable, high solar wind speed conditions (Turner et al. 2013; Chu et al. 2017). Kajdič et al. (2013) found foreshock cavitons to be detected at a rate of ${\sim} 2$ per day. If spontaneous HFAs are due to cavitons, this is their expected rate. The well-known occurrence rate of substorms is once every 2 to 3 hours under favorable, southward IMF conditions (e.g., Jackman et al. 2014). Hence, in comparison, large scale jet impacts on the dayside magnetopause occur much more often than these other transients.

## Properties

Similarly to the previous section, we begin by reviewing some findings of the properties of magnetosheath jets from early case studies. We then move on to a more comprehensive summary of some important jet properties, based on statistical studies, with a view to establish a solid base for comparison with models and simulations.

### Early Results and Case Studies

The first report on jets by Němeček et al. (1998) comprised 11 events observed by Interball-1 and Magion-4 during a high-latitude magnetosheath pass (see Sect. 2 for how these and other events in this section were defined). Figure 2 shows the time interval from which they investigated some typical jet properties. It was established that the jets had durations of tens of seconds up to a minute, which is consistent with a flow-parallel dimension of 0.5 to $2.8\,R_{\mathrm{E}}$, assuming a magnetosheath flow velocity of $300~\mbox{km}/\mbox{s}$. They also reported that the jets were associated with an increase of the ion flux up to a factor of 5 compared to the background level. This corresponds to increases in the dynamic pressure of a factor between 5 (assuming that all the flux increase is due to increased density) and 25 (assuming that all increase is due to increased velocity). Němeček et al. (1998) reported that the events were associated with very small changes in velocity. However, this conclusion seems to be based on rather uncertain computations of the ion bulk velocity. They also claim that there is no correlation between the ion flux and the magnetic field strength for the jets. Looking at Fig. 2, this may be true for some of the events but not for all of them.

Other case studies have reported similar flow-parallel scale sizes based on single spacecraft measurements of event duration. Savin et al. (2008) gave an average duration of $28~\mbox{s}$, based on investigation of one Cluster traversal of the magnetosheath. Using a flow speed of $300~\mbox{km}/\mbox{s}$, this corresponds to $1.3\,R_{\mathrm{E}}$. Archer et al. (2012) used two of the THEMIS spacecraft to estimate the scale size perpendicular to the flow of the jets. They gave a value of 0.2 to $0.5\,R_{\mathrm{E}}$, based on large differences in amplitudes between the two spacecraft, while reporting a flow-parallel scale size of $1\,R_{\mathrm{E}}$. Dmitriev and Suvorova (2012) give a flow-parallel scale size of $4.7\,R_{\mathrm{E}}$, based on a life time of $140~\mbox{s}$ for a single event, while Hietala et al. (2009, 2012) reported on perpendicular scale sizes of at least $1\,R_{\mathrm{E}}$. Finally Gunell et al. (2012) estimated the dimension of a small number of plasmoids—using the definition of Karlsson et al. (2012)—perpendicular to both their velocity and the magnetic field to be $0.2\,R_{\mathrm{E}}$.

Most of the early case studies after Němeček et al. (1998) give direct values of the dynamic pressure of the magnetosheath jets, and report increases over the background magnetosheath by factors of 1.5 to 10 (Savin et al. 2008; Hietala et al. 2009; Archer et al. 2012) or factors of 1 to 7 with respect to the upstream solar wind (Amata et al. 2011; Hietala et al. 2012). On the question of whether the density or the velocity contributes most to the dynamic pressure, the reports vary. We will return to this question below.

The magnetic field variations within the jets also remained unclear in these early studies. Němeček et al. (1998), as described above, reported that there is no correlation between the jet dynamic pressure and magnetic field, so did Savin et al. (2008). On the other hand, Hietala et al. (2009, 2012) reported an increase of the magnetic field, collocated with the jet, Dmitriev and Suvorova (2012) reported a magnetic field decrease, and Archer et al. (2012) a magnetic field rotation.

Better agreement is found regarding the temperature behaviour of magnetosheath jets, with observations of a decrease of the perpendicular ion temperature inside the jets, resulting in a virtually isotropic ion temperature (Hietala et al. 2009; Archer et al. 2012; Dmitriev and Suvorova 2012).

Some of these results are summarized in Table 2, which also contains results described later in this section.

**Table 2:** Summary of some magnetosheath jet properties; “msh” and “sw” mean magnetosheath and solar wind, respectively

Paper	Scale size (parallel)	Scale size (perpendicular)	Increase in dynamic pressure (factor)	Change in $T_{i\perp }$^1^	Magnetic field change	Spacecraft	Comment
Němeček et al. (1998)	0.5 to $2.8\,R_{\mathrm{E}}$^2^		2 to 25 (wrt msh)		None	INTERBALL-1, MAGION-4	
Savin et al. (2008)	$28~\mbox{s}\:\rightarrow \: 1.3\,R_{\mathrm{E}}$ ^3^		1.5 to 4 (wrt msh)		None	Cluster	
Hietala et al. (2009)		$1.2\,R_{\mathrm{E}}$	6 (wrt msh)	Decrease	Increase	Cluster	Substantial change in velocity direction
Amata et al. (2011)			≥1 (wrt sw)			Cluster	
Archer et al. (2012)	1 $R_{\mathrm{E}}$	0.1 to $0.5\,R_{\mathrm{E}}$	3 to 10 (wrt msh)	Decrease	Rotation for some events	THEMIS D and E	No significant change in velocity direction
Dmitriev and Suvorova (2012)	$150~\mbox{s}\:\rightarrow \: 4.7\,R_{\mathrm{E}}$ ^4^		4 to 5 (wrt msh)	Decrease	Decrease	THEMIS E	
Hietala et al. (2012)		1 to $6\,R_{\mathrm{E}}$	2 to 7 (wrt sw)		Increase	Cluster, GOES	
Gunell et al. (2012)		$0.2\,R_{\mathrm{E}}$				Cluster	Plasmoids
Karlsson et al. (2012)	0.3 to $3\,R_{\mathrm{E}}$^5^	0.3 to $10\,R_{\mathrm{E}}$^6^	>1.5 (wrt msh)			Cluster	Fast plasmoids
Archer and Horbury (2013)			2 to 15 (wrt msh)	Decrease $(\delta \rho _{\mathrm{msh}} / \langle \rho _{\mathrm{msh}} \rangle > 0)$	Decrease $(\delta \rho _{\mathrm{msh}} / \langle \rho _{\mathrm{msh}} \rangle < 0.4)$ Increase $(\delta \rho _{\mathrm{msh}} / \langle \rho _{\mathrm{msh}} \rangle > 0.4)$	THEMIS	
Plaschke et al. (2013)			3 to 25 (wrt msh)		Increase (61% of events)	THEMIS	
Gunell et al. (2014)	$4.9\,R_{\mathrm{E}}$ ^7^					Cluster	
Karimabadi et al. (2014)	$2.4\,R_{\mathrm{E}}$	$0.3\,R_{\mathrm{E}}$	6 (wrt msh)	Decrease	Increase		Simulation
Gutynska et al. (2015)	${<} 0.8\,R_{\mathrm{E}}$ ^8^	$0.8\,R_{\mathrm{E}}$ ^9^				THEMIS	Plasmoids
Karlsson et al. (2015b)	$1.2\,R_{\mathrm{E}}$ ^10^ $1.4\,R_{\mathrm{E}}$ ^11^			Decrease	Increase	Cluster	Paramagnetic plasmoids
Plaschke et al. (2016)	$0.71\,R_{\mathrm{E}}$	$1.34\,R_{\mathrm{E}}$				THEMIS	
Hao et al. (2016a)	$1\,R_{\mathrm{E}}$	$0.2\,R_{\mathrm{E}}$	∼4 (wrt msh)	Decrease	Increase		Simulation

^1^With respect to the surrounding magnetosheath

^2–3^Scale size calculated assuming $v_{\mathrm{msh}} = 300~\mbox{km}/\mbox{s}$

^4^Scale size calculated assuming $v_{\mathrm{msh}} = 200~\mbox{km}/\mbox{s}$

^5–6^Parallel and perpendicular refers to the minimum variance direction

^7^Median

^8–9^Parallel and perpendicular refers to the magnetic field direction

^10^Fast plasmoids

^11^Embedded plasmoids

### Plasma Moments and Magnetic Field

Recently a number of statistical studies have been performed, which together with the above case studies, give a more comprehensive picture of the magnetosheath jet properties. We first describe some properties related to the plasma moments such as bulk velocity, density and temperature, as well as the relation to the magnetic field.

#### Dynamic Pressure

As discussed in Sect. 2, the question arises if the increase in dynamic pressure is mainly due to a velocity or a density increase. The early reports from the previous section are disparate, with Archer et al. (2012) quoting a dominating contribution from a velocity increase, while others report comparable contributions from both density and velocity increase (Hietala et al. 2009), or varying relative contributions (Savin et al. 2008; Amata et al. 2011).

**Fig. 12:**
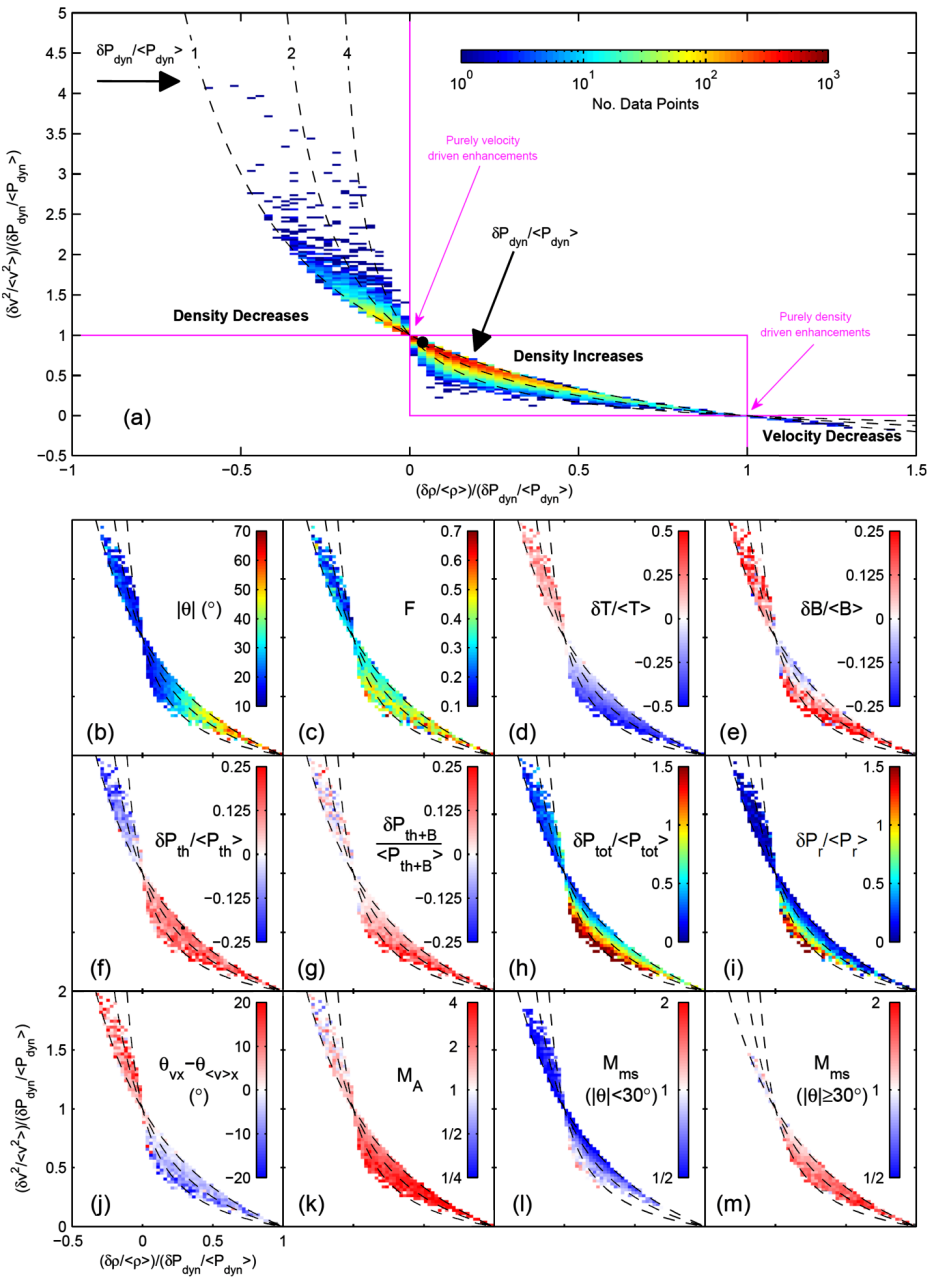
(**a**) Distribution of number of data points of magnetosheath jets in the parameter space of Eq. (3). (**b**)–(**m**) Distributions of indicated parameters in the same space. Figure 3 in Archer and Horbury (2013)

This situation was clarified in the statistical study by Archer and Horbury (2013), based on THEMIS data from the dayside magnetosheath. They showed that the relative change in dynamic pressure to good approximation can be written as

3 $$\begin{aligned} \frac{\delta P_{\mathrm{dyn,msh}}}{\langle P_{\mathrm{dyn,msh}}\rangle } = \frac{\delta \rho _{\mathrm{msh}}}{\langle \rho _{\mathrm{msh}} \rangle } + \frac{\delta (v^{2}_{\mathrm{msh}})}{\langle v^{2}_{\mathrm{msh}} \rangle } + \frac{\delta \rho _{\mathrm{msh}}}{\langle \rho _{\mathrm{msh}}\rangle } \frac{\delta (v^{2}_{\mathrm{msh}})}{\langle v^{2}_{\mathrm{msh}} \rangle }, \end{aligned}$$

where the first two terms on the right hand side are the relative contributions to the dynamic pressure change due to changes in density and velocity (squared), and the last term is a correlation term. The angular brackets denote here a $20~\mbox{min}$ average, as described in Sect. 2.2. The relative density and velocity changes represent a convenient parameter space to represent the jet-related dynamic pressure changes. Figure 12a shows the distribution of dynamic pressure increases in this parameter space, with the number of data points from the jet events showed by the colour scale (Archer and Horbury 2013). Particular values of $\delta P_{\mathrm{dyn,msh}} / \langle P_{\mathrm{dyn,msh}}\rangle $ define curves in this parameter space, shown as black dashed lines.

It can be seen that a large majority of the data points fall between these curves meaning that most relative dynamic pressure changes fall in this range (between 1 and 4), although increases up to a factor of 15 were observed. Furthermore most of the points are found in a region where the relative contribution of the velocity increase is greater than the density increase, although there is a continuum of different relative contributions. This continuum is consistent with the fact that the isolated case studies discussed above can give quite contrasting results.

Although most of the jets are associated with a combination of velocity and density increases, there are actually jets associated with density decreases. Archer and Horbury (2013) argue that these are associated with flux transfer events (FTEs, Russell and Elphic 1978), based on their proximity to the magnetopause and their association with enhancements in magnetic field and temperature, and a velocity close to the local Alfvén velocity.

At the other end of the continuum, there are dynamic pressure increases associated with a dominating or exclusive contribution from density enhancements (marked as ‘Purely density driven enhancements’ in Fig. 12a). These are consistent with the embedded plasmoids reported by Karlsson et al. (2012) and Gutynska et al. (2015). We will return briefly to the relation between plasmoids and velocity increase dominated jets below.

**Fig. 13:**
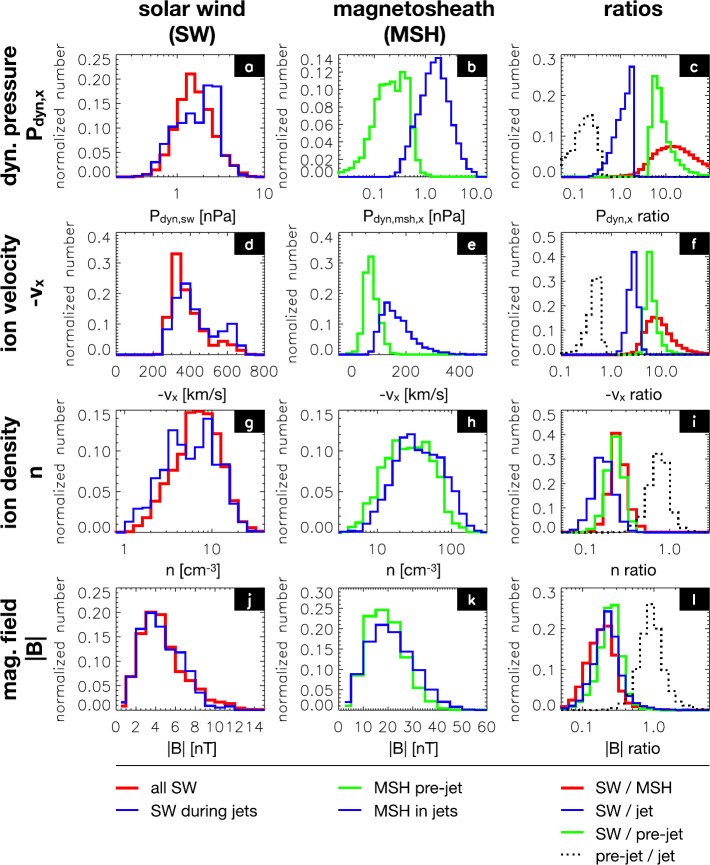
Distributions of solar wind and magnetosheath data for all sheath intervals, jets, and the region surrounding the jets (pre-jet), as well as ratio distributions. After Fig. 5 in Plaschke et al. (2013)

The large statistical study of Plaschke et al. (2013) shows results consistent with the above, with a distribution of ratios of maximum dynamic pressure increases in jets to the dynamic pressure of the surrounding magnetosheath plasma between approximately 3 and 25 (Fig. 13c, dotted curve), and with corresponding approximate ratios of velocity (1 to 3) and density changes (0.7 to 2) (Figs. 13f and 13i). Density increases were observed in 89% of the jet events.

#### Magnetic Field

**Fig. 14:**
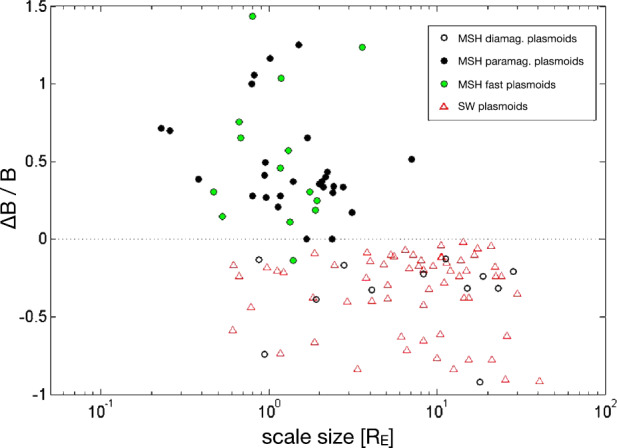
Relative magnetic field change as a function of scale size for solar wind and magnetosheath plasmoids. After Fig. 3 in Karlsson et al. (2015b)

From Fig. 13l, it can be seen that the results of Plaschke et al. (2013) show a distribution of changes in the absolute value of the magnetic field associated with the jets, both corresponding to increases and decreases, but with a maximum corresponding to a slight increase, similar to the distribution of densities (Fig. 13i). This is very similar to the behaviour of the paramagnetic plasmoids reported by Karlsson et al. (2015b) (see Fig. 14), where positive magnetic field changes are associated with the density increase that is the defining property of the plasmoids. Karlsson et al. (2015b) argue that the paramagnetic plasmoids are a subset of (or closely related to) magnetosheath jets. They base their argument on the fact that the paramagnetic plasmoids are not present in the solar wind (as opposed to the diamagnetic ones) similar to the jets, as well as on other similarities such as the magnetic field signatures discussed here, morphology (see Sect. 4.3) and temperature behaviour (see below).

The result of Archer and Horbury (2013) (Fig. 12e), also shows that jets can have both increased and decreased magnetic field magnitudes, but that the jets with a density enhancement greater than 40% almost exclusively are associated with an increase in the magnetic field strength.

#### Temperature

As discussed in Sect. 4.1, Archer et al. (2012) and Dmitriev and Suvorova (2012) noted that the jets are associated with a lower temperature than the surrounding plasma (although only Archer et al. (2012) reported that this decrease was mainly in the perpendicular ion temperature). This behaviour is confirmed by the statistics of Plaschke et al. (2013), which show that both the parallel and perpendicular temperatures are decreased in the jets, with the decrease in the perpendicular temperature considerably larger, leading to a more isotropic temperature. Also Archer and Horbury (2013) showed that jets associated with density increases have a decrease in ion temperature. For jets with a density decrease, on the other hand, they reported a temperature increase, again consistent with the interpretation that these jets were on FTE field lines, which contain hot, magnetospheric plasma. Karlsson et al. (2015b) showed that paramagnetic plasmoids were also associated with a similar decrease of perpendicular ion temperature, again pointing to the similar properties of these structures and jets associated with larger velocity increases.

#### Velocity

Magnetosheath jets often have a velocity considerably greater than the local Alfvén velocity (Archer and Horbury 2013; Plaschke et al. 2013), although jets with density decreases have velocities close to the Alfvén velocity, as expected for FTEs (Archer and Horbury 2013). Some jets are even supermagnetosonic (in Earth’s frame of reference) (Savin et al. 2008, 2012; Hietala et al. 2009, 2012; Archer and Horbury 2013; Plaschke et al. 2013), in which case they may be associated with a local shock at the front of the jet (Hietala et al. 2009, 2012). Archer and Horbury (2013) report that, logically, the majority of jets in the flanks of the magnetosheath, where the ambient flow is generally faster than in the subsolar region, are super-magnetosonic. In contrast, in the subsolar region only about 14% of the jets are super-magnetosonic (Plaschke et al. 2013).

The velocity of magnetosheath jets is not only higher than that of the surrounding magnetosheath plasma, it also often differs in direction, being generally oriented more along the Sun–Earth line (Gunell et al. 2012; Hietala et al. 2012; Hietala and Plaschke 2013; Archer and Horbury 2013; Plaschke et al. 2013). Hietala and Plaschke (2013) reported that the increasing deviation from the surrounding magnetosheath flow with increasing dynamic pressure ratio, and the depth in the sheath, is consistent with a tendency for the jets to continue ‘straight’ along the Sun–Earth line as compared to the background magnetosheath flow. Plaschke et al. (2013) give a number of $28.6^{\circ }$ for the median deflection of the jets versus the background magnetosheath flow, while Hietala and Plaschke (2013) cited a number of $20^{\circ }$ to $34^{\circ }$. On the other hand, Archer and Horbury (2013) claimed that the deflections are considerably smaller than this, typically only a few degrees.

Granting that paramagnetic plasmoids are basically the same phenomenon as magnetosheath jets, also the results of Karlsson et al. (2012) are consistent with the above results, showing that fast plasmoids typically have a velocity more aligned with the Sun–Earth line. They, however, give no number of the typical deflections. Here more research may be needed.

### Morphology

The early results have established that magnetosheath jets have scale sizes of the order of $1\,R_{\mathrm{E}}$, but have given somewhat inconsistent results of the more detailed morphology. For example, Archer et al. (2012) report on a longer scale size parallel to the jet flow than perpendicular to it, while Hietala et al. (2009) and Hietala et al. (2012) give upper limits on the perpendicular dimensions which are larger than the parallel ones of Archer et al. (2012). A more systematic investigation of the scale sizes parallel and perpendicular to the jet velocity was performed by Plaschke et al. (2016). They report that based on a statistical distribution of multi-spacecraft correlations (assuming a cylindrical geometry of the jets), both the flow-parallel and perpendicular diameters fit well to exponential distributions (Eq. (2) in Sect. 3.2) with a characteristic values of $0.71\,R_{\mathrm{E}}$ for the parallel dimension and $1.34\,R_{\mathrm{E}}$ for the perpendicular diameter. Even though the parallel and perpendicular diameters were estimated independently, Plaschke et al. (2016) interpreted the results as the jets having a pancake-like geometry.

The parallel scale size of the above investigation is consistent with the scale size along the GSE $x$ direction of $0.6\,R_{\mathrm{E}}$ reported by Plaschke et al. (2013), since the background magnetosheath velocity is close to this direction near the subsolar point, where the data in this investigation were taken. The perpendicular scale size was investigated by a similar method by Gunell et al. (2014) using a smaller data set from the Cluster mission. They give a perpendicular diameter of 4.2 to $7.2\,R_{\mathrm{E}}$, which obviously is considerably larger than the above result. It should be pointed out that they deliberately overestimated the scale sizes. Furthermore, Gunell et al. (2014) give a median upper limit of the flow-parallel scale size of $4.9\,R_{\mathrm{E}}$.

**Fig. 15:**
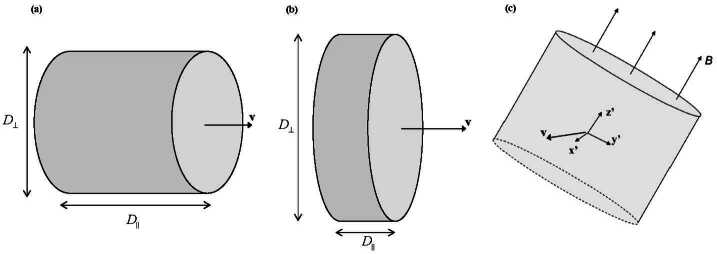
Conceptual figures of the morphology interpretations of the results of (**a**) Archer et al. (2012), (**b**) Plaschke et al. (2016), and (**c**) Karlsson et al. (2012)

Karlsson et al. (2012) considered the relation of the morphology to the magnetic field, performing a minimum variance analysis on 36 plasmoid events, and investigating the scale size by Cluster multipoint techniques. They report that the shortest scale size was associated with the minimum variance direction, which was close to perpendicular to the magnetic field for almost all events. The scale size along the magnetic field and in the remaining direction were typically 3 to 10 times larger than that of the minimum variance direction. Karlsson et al. (2012) interpreted this in terms of flattened, severed flux tubes. On the other hand, Gutynska et al. (2015) reported that the scale size perpendicular to the magnetic field direction was longer than in the direction of the magnetic field; based on a two-spacecraft correlation study, using THEMIS data, they gave a dimension perpendicular to the magnetic field of $0.8\,R_{\mathrm{E}}$, but no specific number for the dimension along the magnetic field. Figure 15 shows the geometries that were concluded by Archer et al. (2012), Plaschke et al. (2016) and Karlsson et al. (2012), respectively.

Karlsson et al. (2012) also reported that the minimum variance direction was systematic, in that it pointed close to parallel to the bow shock and magnetopause normals, i.e. the structures were lined up in the general direction of the bow shock/magnetopause. A consistent result was reported by Gutynska et al. (2015), although the observations were concentrated to the subsolar region. Karlsson et al. (2015b) noted that the minimum variance direction of the plasmoids did not necessarily coincide with the flow velocity, i.e. the structures could be ‘tilted’ with respect to the flow direction (see Fig. 15c). This means that a simple estimate of the flow-parallel scale size by calculating $\int _{t_{\mathrm{start}}}^{t_{\mathrm{stop}}}v\,\mathrm{d}t$ over the jet event may overestimate the shortest dimension of the jet. By comparing this method with the full multi-spacecraft method of Karlsson et al. (2012), for a subset of the events Karlsson et al. (2015b) showed that the integral method typically yields a result that is more than a factor of 2 greater than that of the multi-spacecraft method. Using the velocity-integral method, in order to obtain flow-parallel scale-sizes comparable to other studies, they found median scale sizes for embedded and fast paramagnetic plasmoids to be $1.4\,R_{\mathrm{E}}$ and $1.2\,R_{\mathrm{E}}$, respectively, yielding further support for identifying the paramagnetic plasmoids as a subset of the magnetosheath jets.

The morphology of the magnetosheath jets and plasmoids is an important property to compare with predictions of theories and simulations, which means that this is an area where more research needs to be done. It is for example interesting to investigate whether jets with different properties (e.g. different magnetic signatures or relative importance of velocity or density increases) have different morphologies. This may be a way to address the somewhat inconsistent results to date (see also outlook Sect. 9.2).

### Other Properties

Bursty bulk flows (BBFs) have recently been shown to emit various types of plasma waves, which may contribute to the dissipation of the flow velocity (e.g., Ergun et al. 2015; Breuillard et al. 2016). One might therefore expect that magnetosheath jets, due to their similarities with BBFs in some aspects (see Sect. 7) would also emit waves. So far this question has only been addressed by Gunell et al. (2014), who studied the wave activity inside a number of jets (or ‘plasmoids’ in the nomenclature of Gunell et al. (2014)). They observed both lower hybrid and whistler mode emissions, at power densities considerably larger than in the background magnetosheath plasma. These kind of waves may be important for possible impulsive penetration of jets into the magnetosphere, since they may increase the rate of diffusion of the large magnetospheric field into the jets (e.g., Hurtig et al. 2005) (see also Sect. 6).

Another question that has not been much studied is the downstream evolution of magnetosheath jets. As discussed in Sect. 3, Plaschke et al. (2013) report that jets are more common close to the bow shock than close to the magnetopause, for a data set limited to the subsolar region with its relatively narrow extent of the magnetosheath. This is consistent with the observations by Dmitriev and Suvorova (2015), who reported on THEMIS observations of a jet, which showed a decrease of velocity as it moved towards the magnetopause. Archer and Horbury (2013), covering the whole dayside (which is still a relatively limited region), reported that there is no clear change in observation probability with distance from the bow shock. They do point out that density driven jets are more likely to occur at the flanks, which is maybe consistent with the finding of Karlsson et al. (2015b) that fast plasmoids are only found for $x > 2\,R_{\mathrm{E}}$ (in GSE), while embedded plasmoids are found further downstream for $x > -5\,R_{\mathrm{E}}$. This might either indicate that the jets are braked as they propagate further downtail, or that their velocity increase becomes insignificant as the whole magnetosheath flow speeds up.

### Simulations

Recently the small-scale, transient variation of the magnetosheath flow associated with magnetosheath jets and plasmoids has attracted the attention of the simulation community. In particular the possibility of global hybrid and fully kinetic simulations potentially represents a great step forward in studying jets and plasmoids. In Sect. 5 we will discuss simulations from the viewpoint of the jet generation mechanisms. In this subsection we focus on some jet properties that can be extracted from the simulations and compared to observations.

**Fig. 16:**
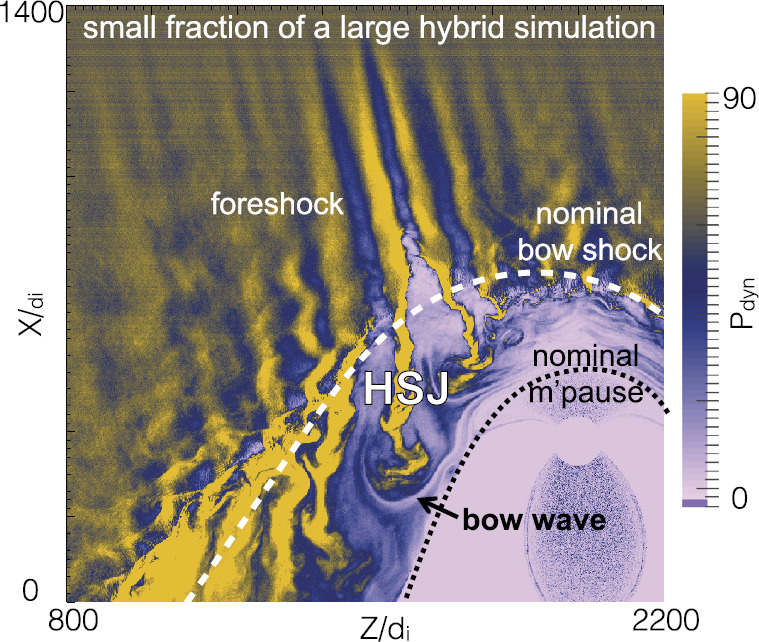
Dynamic pressure from one of the simulation runs of the global hybrid simulation of Karimabadi et al. (2014). For this quasi-parallel bow shock, one can make out foreshock structuring, a corrugated bow shock, and jet-like increases of dynamic pressure in the magnetosheath, extending almost to the magnetopause

In a recent global hybrid simulation by Karimabadi et al. (2014), jet-like structures were observed to penetrate from the foreshock of the quasi-parallel bow shock into the magnetosheath (see Fig. 16). These structures are associated with an increase of magnetic field strength and density, and have a velocity comparable to that of the upstream solar wind. From the figure we can estimate that the dynamic pressure is increased by a factor of around 6 with respect to the surrounding magnetosheath plasma. These properties are consistent with the observations described above. Regarding the morphology, the structures are clearly elongated in the direction of the flow (see Fig. 5 in Karimabadi et al. 2014), and we estimate the perpendicular and parallel scale sizes to be ${\sim} 40\,d_{i} \approx 0.3\,R_{\mathrm{E}}$, and ${\sim}300\,d_{i} \approx 2.4\,R_{\mathrm{E}}$, respectively, where $d_{i}$ is the ion inertial length. To calculate it, we used a typical magnetosheath plasma density of $20~\mbox{cm}^{-3}$ (Phan et al. 1994).

Hao et al. (2016a) have also investigated the formation of jets downstream of a quasi-parallel shock with a local 2D hybrid simulation. They report on structures downstream of the shock, which have many properties in common with the observed magnetosheath jets. The jets of the simulation have an increased velocity as compared to the background magnetosheath plasma. That velocity increase is directed along the magnetic field and is associated with increases in density and magnetic field strength, as well as a lower temperature, compared to the background. The scale size is similar to that of Karimabadi et al. (2014), with a flow-parallel dimension of around $1\,R_{\mathrm{E}}$, and a perpendicular diameter of $0.2\,R_{\mathrm{E}}$. One feature of the simulated jets that is not consistent with observations is that the plasma flow direction of the jets is almost tangential to the bow shock. This could be partly due to the relatively low Mach number (5.5) used in the simulation.

Omidi et al. (2014a) report on small-scale structures downstream of the quasi-parallel bow shock, which they call ‘magnetosheath filamentary structures’ (MFS). Some of the MFS properties are consistent with the (embedded) plasmoids in Karlsson et al. (2012, 2015b) and Gutynska et al. (2015): they are not associated with an increased flow velocity, but with an increase in plasma density of around 50%, and a decrease in temperature. Their scale-size along the nominal magnetosheath flow was around $0.2\,R_{\mathrm{E}}$, with the perpendicular scale around $1.6\,R_{\mathrm{E}}$. However, the density increases are not correlated with any magnetic field variation, their orientation is not aligned with the bow shock in the way reported by Karlsson et al. (2012), and Gutynska et al. (2015), and the MFS have a quasi-periodic structure. Recently, Omidi et al. (2016) also reported on jet formation associated with spontaneous hot flow anomalies (SHFAs). These simulations will be discussed in Sect. 5.2.4.

It is clear that the types of simulations described above show that small-scale, transient, coherent magnetosheath structures can appear behind the quasi-parallel bow shock. At the moment there is no consensus on simulation results of the properties of such structures, the details of their formation, and how they compare to the observed properties of magnetosheath jets and plasmoids. However, continued simulation efforts, including evaluation of the effects of solar wind Mach number, 2D vs 3D, and electron kinetic physics, will be an important tool in understanding the generation and effects of magnetosheath jets and plasmoids.

## Generation Mechanisms

In this section we review and discuss the mechanisms that have been proposed to explain the origin of magnetosheath jets. The origin of jets is controversial and has been attributed to different mechanisms: rippling of the bow shock (Hietala et al. 2012, 2009; Plaschke et al. 2013; Hao et al. 2016a; Karimabadi et al. 2014), solar wind discontinuities interacting with the bow shock (Archer et al. 2012), hot flow anomalies (HFAs) at the bow shock (Savin et al. 2012), foreshock magnetosonic waves interacting with the bow shock (Omidi et al. 2016), foreshock short large amplitude magnetic structures (SLAMS) interacting with shock ripples (Karlsson et al. 2015b), and magnetic reconnection inside the magnetosheath (Retinò et al. 2007; Phan et al. 2007). High speed plasmas in the magnetosheath have also been explained in terms of a so called slingshot effect by Chen et al. (1993) and Lavraud et al. (2007).

As already stated, we know that jets occur preferentially during radial IMF and/or downstream of the quasi-parallel shock, which suggests that their origin is often related to the quasi-parallel bow shock and phenomena occurring in the foreshock region (see Sect. 3). In a recent study of subsolar magnetosheath observations, Hietala and Plaschke (2013) concluded that 97% of the observed jets could be consistent with origin at the bow shock rippled structure. To understand shock rippling and how upstream magnetic structures can influence the bow shock and magnetosheath, it is necessary to discuss first the properties of the bow shock and foreshock regions. We start by doing this below and continue describing how foreshock phenomena and shock rippling can be associated to jet origin, using both observations and simulations.

### Introduction to Foreshock Phenomena

**Fig. 17:**
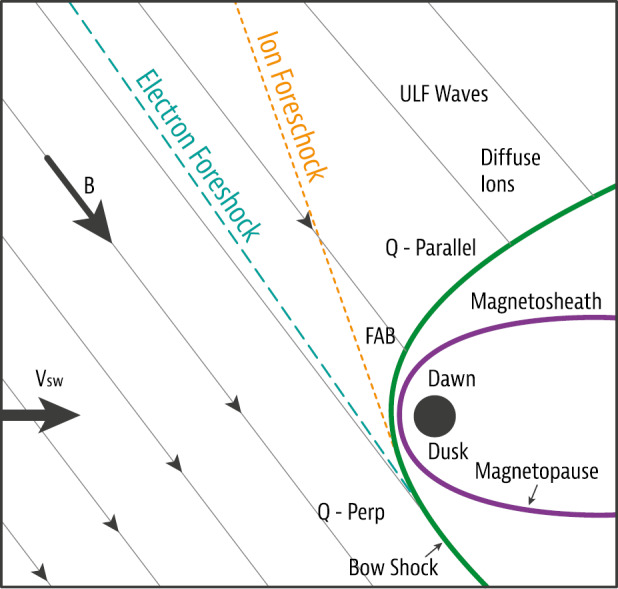
Schematic view of the Earth’s bow shock, and foreshock showing the regions of quasi-parallel and quasi-perpendicular shock on the ecliptic plane. Reprinted from Blanco-Cano (2010), with the permission of AIP Publishing

The interaction of the supermagnetosonic solar wind with Earth’s magnetic field leads to the formation of a bow shock in front of our planet (Balogh et al. 2005). Figure 17 shows a schematic view of the region of solar wind interaction with the magnetosphere. In that illustration, the solar wind flows in from the left and the interplanetary magnetic field orientation is $45^{\circ }$ with respect to the Sun–Earth (radial) direction. The angle between the upstream magnetic field and the Sun–Earth line is on average $45^{\circ }$, but varies widely. As already mentioned in the introduction, the solar wind is decelerated, deviated, heated, and compressed when passing through the shock. The region downstream of the shock, where jets are observed, is called the magnetosheath. It contains shocked solar wind, which interacts with Earth’s magnetosphere. As mentioned above, upstream phenomena, both in the solar wind, and at the foreshock-bow shock region, can modulate the characteristics of the magnetosheath plasma. Typical Mach numbers of the Earth’s dayside shock are usually high with Alfvénic and magnetosonic Mach numbers ($M_{\mathrm{A}}$ and $M_{\mathrm{ms}}$, respectively) ranging between $6 \leq M_{\mathrm{A}} \leq 7$ and $5 \leq M_{\mathrm{ms}} \leq 6$ (Winterhalter and Kivelson 1988). Such high Mach numbers imply that the bow shock is supercritical, so that it dissipates the incoming solar wind kinetic energy by reflecting a portion of the incoming solar wind particles back upstream (see, Woods 1969; Paschmann et al. 1979; Gosling and Thomsen 1985).

Due to the bow shock’s curvature and the orientation of the upstream IMF, the angle between the bow shock normal and the IMF $\theta _{Bn} $ changes across it’s surface and can be divided into quasi-parallel ($\theta _{Bn} \leq 45^{\circ }$) and quasi-perpendicular ($\theta _{Bn} > 45^{\circ }$) regions. At the quasi-parallel section of the bow shock, the reflected particles can escape upstream producing a complex and extended shock structure, and an ion foreshock region ahead of the shock where various suprathermal ion distributions and a variety of waves exist (Eastwood et al. 2005). As shown in Fig. 17, the foreshock region is magnetically connected to the shock and permeated by a variety of ultra low frequency (ULF) waves with frequencies much less than the ion cyclotron frequency $f_{\mathrm{ci}}$. The waves are generated by various kinetic instabilities (Gary 1993; Blanco-Cano and Schwartz 1997a,b) due to the backstreaming ion interaction with the incoming solar wind. The ULF waves include “30 second waves”, “10 second waves”, “3 second waves”, and “1 Hertz waves” (see, for example, Burgess 1997; Eastwood et al. 2005; Wilson 2016).

**Fig. 18:**
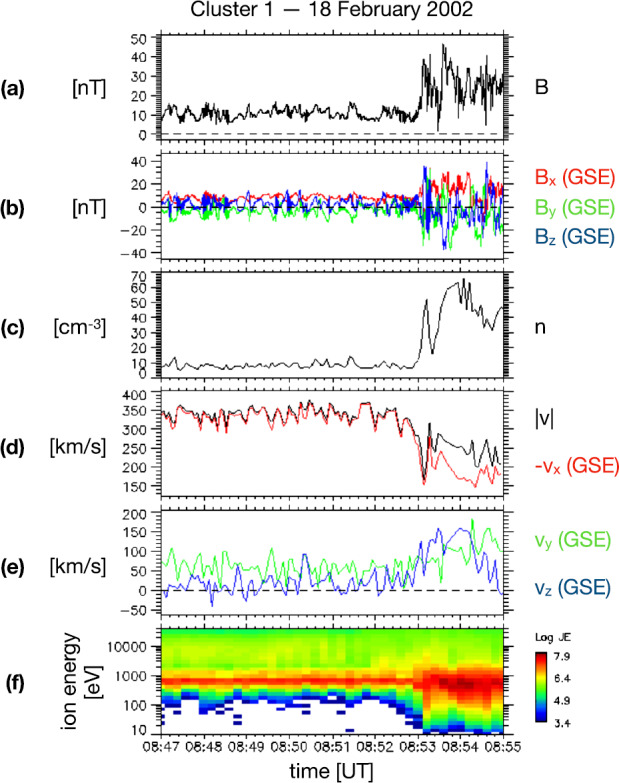
Cluster observations of the Earth’s foreshock, bow shock and magnetosheath. Panels show the magnitude of the magnetic field, its components, plasma density, velocity (magnitude and components, and proton energy spectrum

Figure 18 shows an example of a quasi-parallel shock crossing observed by Cluster 1 on 18 February 2002. Panels a and b show that the upstream region is filled with compressive magnetic field and plasma fluctuations. Furthermore, the shock transition is not sharp as for quasi-perpendicular shocks (see, for example, Fig. 3 in Blanco-Cano 2010), but is composed by various layers of very large amplitude fluctuations. The downstream magnetic field is highly perturbed with compressive large amplitude fluctuations. Panels d and e show that the solar wind is decelerated and deviated at the shock. The ion energy spectrum (panel f) shows suprathermal reflected ions upstream of the shock as green and yellow regions with energies $1 \times 10^{3}~\mbox{eV} < E < 2 \times 10^{4}~\mbox{eV}$. It is also possible to see a heated solar wind beam as a wide red trace downstream of the shock.

The most studied low frequency foreshock waves are the “30 second waves”, which as their name suggests, have periods of ${\sim} 30~\mbox{s}$. While some of these waves are quasi-monochromatic and sinusoidal, propagating almost parallel to the magnetic field, others can propagate at oblique angles (${\sim} 20^{\circ }$ to $40^{\circ }$ with respect to the ambient magnetic field). They are very compressive (Hoppe et al. 1981), featuring large amplitudes $\delta B\sim 5~\mbox{nT}$ (peak-to-peak), sometimes reaching $\delta B / B \sim 1$. The fluctuations have wavelengths ${\sim} 1$ to $3\,R_{\mathrm{E}}$ (Archer et al. 2005), with correlation length perpendicular to the wave vector of 8 to $18\,R_{\mathrm{E}}$. ULF waves propagate sunwards with phase speeds of the order of the Alfvén speed, i.e., much smaller than the solar wind speed. As a consequence, the waves are carried back by the flow towards the shock. ULF foreshock 30 second waves are responsible for many of the phenomena at the quasi-parallel shock, such as the variability in the density of reflected ions, variability in the shock ion heating, the cyclic shock reformation, and shock rippling (see for example Burgess 1989; Mazelle et al. 2003; Meziane et al. 2001).

A detailed description of Earth’s foreshock wave phenomena can be found in Eastwood et al. (2005) and Wilson (2016). The evolution of ULF waves, the interaction between them, and their interaction with ion distributions and ion density gradients can lead to the generation of a variety of foreshock transients like shocklets, SLAMS, cavitons, and spontaneous hot flow anomalies (SHFAs) (see Sects. 5.2.1 and 5.2.3 and references therein). In turn, these transients can also contribute to shock structure, reformation and rippling and participate indirectly/directly in the formation of magnetosheath jets. Below we give a brief description of such transients and discuss how they can be related to jet origin. Thereafter, we will discuss the roles of other large scale solar wind structures/discontinuities with respect to jet origin.

### Origin of Jets at the Quasi-parallel Bow Shock

#### Shocklets and SLAMS

**Fig. 19:**
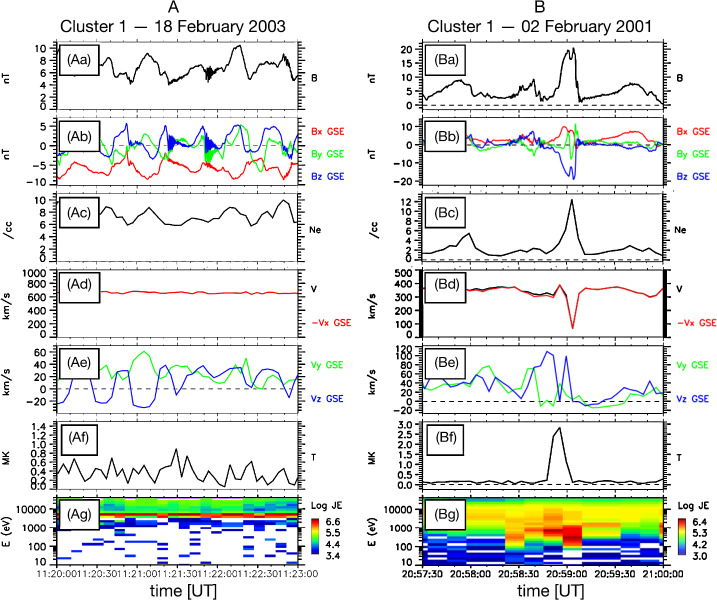
(**A**) shows an example of a region permeated by shocklets observed by Cluster 1 on February 18 2003. (**B**) shows an example of a SLAMS on February 2 2001. Panels (a) to (g) in the figures have the same format as Fig. 18

ULF waves are convected towards the shock by the solar wind. Some of them can steepen forming large amplitude ($\delta B \sim 5~\mbox{nT}$ peak to peak) compressive structures known as shocklets (Hoppe et al. 1981). Figure 19A shows an example of a region permeated by shocklets. These structures are magnetosonic, and appear associated with diffuse ions. Some of them have a whistler packet attached. Shocklets have $\delta B / B_{0} < 2$ with scale sizes comparable to the “30 second waves”, i.e., up to a few $R_{\mathrm{E}}$ (Hoppe et al. 1981; Le and Russell 1994; Lucek et al. 2002). The “discrete wave packets” associated with shocklets have wavelengths of 30 to $2100~\mbox{km}$ and propagation angles with respect to the magnetic field of approximately $20^{\circ }$ to $30^{\circ }$ (Russell et al. 1971; Hoppe et al. 1981). These are whistler mode waves radiated at the steepened edge of the shocklet due to dispersion. As in the case of ULF waves, shocklets propagate sunwards with phase speeds much smaller than the solar wind, so they are convected back towards the shock by the flow.

SLAMS (Schwartz and Burgess 1991) are large amplitude magnetic pulsations upstream of the quasi-parallel shock (Thomsen et al. 1990). As shown in Fig. 19B, the magnetic field inside them shows enhancements by a factor of 3 to 5 with respect to the ambient value, and their typical durations are on the order of ${\sim} 10~\mbox{s}$. As in the case of shocklets, SLAMS are magnetosonic with the density inside them in phase with the magnetic field magnitude. SLAMS also propagate sunward in the plasma frame of reference but are carried earthward by the solar wind, as their phase speed is much lower than the solar wind speed. Several studies have focused on explaining SLAMS origin. One of the possible explanations is that they grow due to the nonlinear interaction of compressive ULF waves with gradients in the diffuse ion densities (see, for example, Scholer et al. 2003; Tsubouchi and Lembège 2004). SLAMS have smaller scale sizes than shocklets and ULF waves (Lucek et al. 2002). According to Lucek et al. (2004b, 2008), their scale sizes are ${\gtrsim} 1000~\mbox{km}$ and ${\sim} 1300~\mbox{km}$ parallel to the shock normal and tangential to the shock surface, respectively.

ULF waves, shocklets and SLAMS can merge into the shock, contribute to the quasi-parallel shock reformation process, and form an extended shock transition region that changes in space and time. As stated in Schwartz and Burgess (1991), the finite extent of SLAMS gives rise to inter-SLAMS regions of unshocked solar wind plasma that contain a mixture of different ion populations (diffuse, field aligned) and become entrained into the downstream flow. The quasi-parallel bow shock then consists of a patchwork of these structures rather than being a well defined single surface (see Fig. 1 in Schwartz and Burgess 1991).

**Fig. 20:**
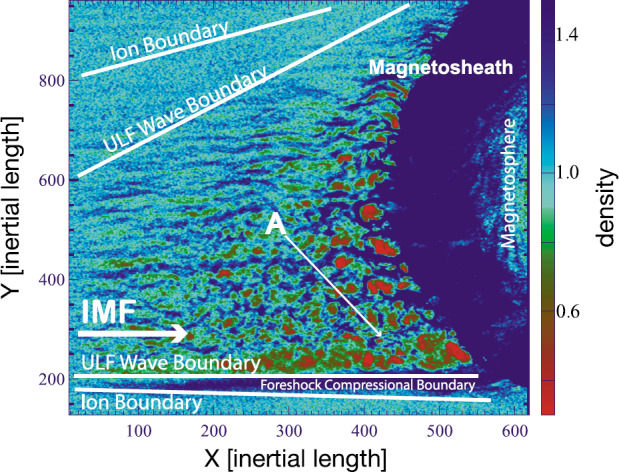
Global hybrid simulation results of the solar wind interaction with a magnetosphere. Colors represent the plasma density normalized to the unperturbed upstream solar wind value. The foreshock region with compressive waves, foreshock cavitons (red colored regions, see Sect. 5.2.3) as well as the bow shock rippled structure can easily be identified. The solar wind is arriving from the left and the IMF is radial along the X axis. The foreshock compressional boundary is also indicated. Adapted from Blanco-Cano et al. (2011)

The interaction of ULF waves, shocklets and SLAMS with the shock can also lead to large changes in the magnetic field direction at the bow shock surface, producing a highly corrugated/rippled shock surface (see, for example, Schwartz and Burgess 1991; Lucek et al. 2008; Omidi et al. 2005; Blanco-Cano et al. 2009). Figure 20 shows the local curvature variations that the shock suffers due to rippling. The fact that the shock is not homogeneous results in the solar wind being processed in a non-uniform way by the shock as the flow crosses into the downstream region.

#### Jet Relation to Waves, Shocklets and SLAMS

Since the first report of magnetosheath jets by Němeček et al. (1998), their origin has been linked to foreshock phenomena and to the quasi-parallel shock. Němeček et al. (1998) reported that jets in the magnetosheath were found in plasma downstream of the quasi-parallel shock. They suggested that jet origin was related to magnetic field discontinuities originating in the foreshock and interacting with the bow shock.

**Fig. 21:**
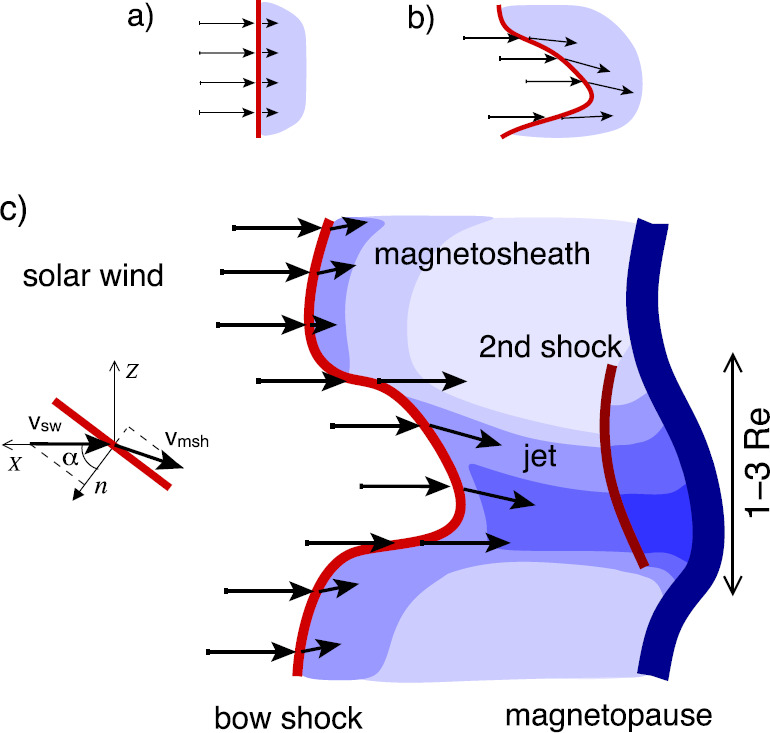
Sketch of bow shock rippling and of the mechanism leading to magnetosheath jets as the plasma crosses the shock. Arrows indicate the direction and magnitude of the flow velocity. After Fig. 1 in Hietala et al. (2012)

In more recent years, the origin of magnetosheath jets has been proposed in terms of shock rippling (local curvature effects) by Hietala et al. (2009, 2012), and Hietala and Plaschke (2013). As explained by these authors, magnetosheath jets may be formed when the solar wind plasma crosses through locally inclined parts of the shock rippled surface (see Fig. 21b). The flow there suffers less deceleration and heating than when the shock normal is parallel to the incoming flow velocity (see Fig. 21a). From the Rankine-Hugoniot jump conditions we know that a high Mach number shock mainly decelerates the component of the upstream speed $v_{\mathrm{sw}}$ that is normal to the shock front, i.e., $v_{\mathrm{msh,}n} = v_{\mathrm{sw,}n} /r$, while the tangential component stays approximately the same: $v_{\mathrm{msh,}t} \sim v_{\mathrm{sw,}t}$. Here $r$ is the shock compression ratio, the index $n$ denominates the velocity component parallel to the shock normal and the index $t$ the velocity perpendicular to it. The indices sw and msh refer to the upstream and downstream regions, respectively. For a high $M_{\mathrm{A}}$ shock, the compression rate is typically 4. As sketched in Fig. 21a, the shock is very efficient in decelerating the plasma when the shock normal $\mathbf {n}$ and $\mathbf {v}_{\mathrm{sw}}$, are almost parallel, so that $v_{\mathrm{msh}} = (1/r) v_{\mathrm{sw}}$, and $\rho _{\mathrm{msh}} = r \rho _{\mathrm{sw}}$. In this case the dynamic pressure is smaller on the downstream side than in the upstream region, with

4 $$ P_{\mathrm{dyn,msh}} = \rho _{\mathrm{msh}} v_{\mathrm{msh}}^{2} = \frac{1}{r}\rho _{\mathrm{sw}} v_{\mathrm{sw}}^{2} = \frac{1}{r} P_{\mathrm{dyn,sw}}. $$

In contrast, when the upstream velocity is at a large angle with respect to the shock normal as at the inclined locations due to rippling, the shock can deviate the flow but the downstream speed remains close to its upstream value (see Fig. 21b), i.e., $v_{\mathrm{msh}} \sim v_{\mathrm{sw}}$ near the edges of the ripple. Assuming the plasma is still compressed ($\rho _{\mathrm{msh}} = r \rho _{\mathrm{sw}}$), the dynamic pressure can in fact be larger on the downstream side, than in the upstream region:

5 $$ P_{\mathrm{dyn,msh}} \sim r \rho _{\mathrm{sw}} v_{\mathrm{sw}}^{2} = r P_{\mathrm{dyn,sw}}. $$

A more generalized calculation in Hietala and Plaschke (2013) gives for the dynamic pressure ratio in the GSE $x$ direction:

6 $$ \frac{P_{\mathrm{dyn,msh,}x}}{P_{\mathrm{dyn,sw,}x}} = \frac{(\cos ^{2}\alpha +\frac{M_{\mathrm{A}n}^{2} - 1}{M_{\mathrm{A}n}^{2} - r(\alpha , M_{\mathrm{A}n}, \beta )} r(\alpha , M_{\mathrm{A}n},\beta )\sin ^{2} \alpha )^{2}}{r(\alpha , M_{\mathrm{A}n},\beta )}. $$

Here we have highlighted that the shock compression ratio $r$ is a function of the different shock parameters: the shock tilt angle $\alpha $ (between the upstream solar wind velocity $\mathbf {v}_{\mathrm{sw}}$ and the local shock normal $\mathbf {n}$, Fig. 21c), the plasma $\beta $, and the normal Alfvén Mach number $M_{\mathrm{A}n} = v_{\mathrm{sw,}n} \sqrt{\mu _{0} \rho _{\mathrm{sw}}}/B_{\mathrm{sw,}n}$ which corresponds with $v_{\mathrm{sw}} \sqrt{\mu _{0} \rho _{\mathrm{sw}}}/B_{\mathrm{sw}}$ for $\mathbf {B}_{\mathrm{sw}} \parallel \mathbf {v}_{\mathrm{sw}}$.

In the case of a rippled quasi-parallel shock, as the one sketched in Fig. 21c, there would be regions downstream of the shock with slow magnetosheath plasma and regions where jets formation can occur, with fast and compressed plasma. Due to their high dynamic pressure, the jets may propagate all the way to the magnetopause and perturb it. In some cases where the flow is super-magnetosonic, a weak shock may appear ahead of them, as depicted in Fig. 21c as well.

As already discussed in Sect. 4, Plaschke et al. (2016) used THEMIS jet observations to determine the scale sizes of jets along their direction of propagation and perpendicular to it. They found characteristic scales of $1.34\,R_{\mathrm{E}}$ (perpendicular) and $0.71\,R_{\mathrm{E}}$ (parallel direction), respectively. Although not much is known about the scales of shock ripples at the quasi-parallel bow shock, it is expected that they should have sizes close to the wavelengths of ULF waves (Archer et al. 2005; Le and Russell 1994) and scale sizes of shocklets and SLAMS (${\sim } 0.2$ to $3\,R_{\mathrm {E}}$, i.e., Lucek et al. 2002). Hietala and Plaschke (2013) modeled shock ripples as sinusoidal surface perturbations, such that the jet would be created by the perturbation’s steep sector with a width of ${\sim} 0.5$ times the wavelength of the perturbation. The authors found that the THEMIS magnetosheath dynamic pressure ratio distribution near the quasi-parallel bow shock was well fitted with a flow arising from ripples with an amplitude (along the average local shock normal) to wavelength ratio of ${\sim} 9\%$. If we scale the modeling result with the observational estimate of jet transverse size of about 0.5 to $1\,R_{\mathrm{E}}$, this would correspond to ripples with an amplitude of 500 to $1000~\mbox{km}$. This is quite similar to the scale size of SLAMS, but more studies are clearly needed to confirm these inferred ripple scales and their relation to foreshock structures.

Numerical simulations have also been very useful in studying jet origin. In a recent study, Omidi et al. (2016) performed global hybrid simulations, and showed that jets in the magnetosheath (identified basically by their speed) may be linked to foreshock fast magnetosonic waves convecting into the bow shock. It is expected that these waves contribute to shock rippling, supporting the mechanism proposed by Hietala et al. (2009, 2012). The scale sizes of ripples caused by these waves need to be further investigated. The simulations presented by Omidi et al. (2016) also show changes in the scale of the jets due to shock Mach number. At lower Mach number jets can have sizes transverse to the flow of ${\sim} 50$ ion inertial lengths (at $1\,\mathrm{AU}$ one ion inertial length is ${\sim} 100~\mbox{km}$ therefore jets transverse size is a bit less than $1\,R_{\mathrm{E}}$), while at higher Mach numbers the jets are thinner, as small as ${\sim} 10$ ion inertial lengths (i.e., ${\sim} 1000~\mbox{km}$). As discussed in Omidi et al. (2016), the change of jet size is linked to the amount of turbulence in the magnetosheath which increases with shock Mach number, with jets becoming less coherent. In other words, as suggested by Hietala et al. (2012) and shown in Fig. 21, jet size is related to the scale sizes of ripples in the shock, but it can change and be modulated by local magnetosheath properties.

Enhancements in dynamic pressure identified as jets have also been found in the simulations of Karimabadi et al. (2014) (see Fig. 16). These downstream structures have speeds similar to the solar wind flow; they can form a bow wave in the magnetosheath. The simulations of Karimabadi et al. (2014) show that jet origin is consistent with shock rippling: The simulated bow shock surface was highly rippled due to foreshock turbulence.

Hao et al. (2016a) and Hao et al. (2016b) studied the formation of magnetosheath jets using local 2D hybrid simulations. These authors identify jets by their speed and their simulation results also support the idea of jet formation by shock rippling, i.e., local curvature effects. As stated in Hao et al. (2016b), jets can be formed because upstream ions passing through different parts of a shock ripple, obtain different velocities after their interaction with the shock. They have also showed that ripples observed along the shock surface can have spatial scales of the order of 1.5 times the wavelength of upstream ULF waves. The jets in their simulations have sizes of 0.3 times the wavelength of upstream waves, i.e., smaller than in observations.

In parallel to the above mentioned works, Karlsson et al. (2015b) have studied paramagnetic plasmoids, which may be considered a subset of magnetosheath jets. The authors link the origin of these plasmoids to SLAMS. According to Schwartz et al. (1992) and Behlke et al. (2004), small amplitude SLAMS have smaller plasma frame propagation velocity than larger amplitude SLAMS, so that they can have a net anti-sunward velocity in the planetary frame. Karlsson et al. (2015b) suggested that sometimes the low amplitude SLAMS may cross the bow shock and enter the magnetosheath. If their velocity is normal to the bow shock, SLAMS can be decelerated as predicted by the Rankine-Hugoniot relations, and become an “embedded plasmoid”, moving with the same speed as the surrounding magnetosheath plasma. If SLAMS encounter local corrugations due to shock rippling, and cross the bow shock almost tangentially, then SLAMS may suffer almost no deceleration and result in fast paramagnetic plasmoids in the terminology of Karlsson et al. (2012, 2015b), moving faster than the surrounding sheath plasma.

Another type of structures studied by Karlsson et al. (2015b) are the diamagnetic plasmoids. As the name suggests the B magnitude and the plasma density $n$ inside them are anticorrelated with $n$ increasing inside them. Diamagnetic plasmoids are embedded structures, propagating at the local solar wind velocity. Karlsson et al. (2015b) observed that these structures already exist in the upstream solar wind, magnetic holes in the solar wind (Turner et al. 1977; Tsurutani et al. 2011) being a plausible candidate, and are convected across the bow-shock into the magnetosheath. Since their densities are larger than those of the surrounding plasma, diamagnetic plasmoids are also regions of increased $P_{\mathrm{dyn,msh}}$.

#### Cavitons and SHFAs

**Fig. 22:**
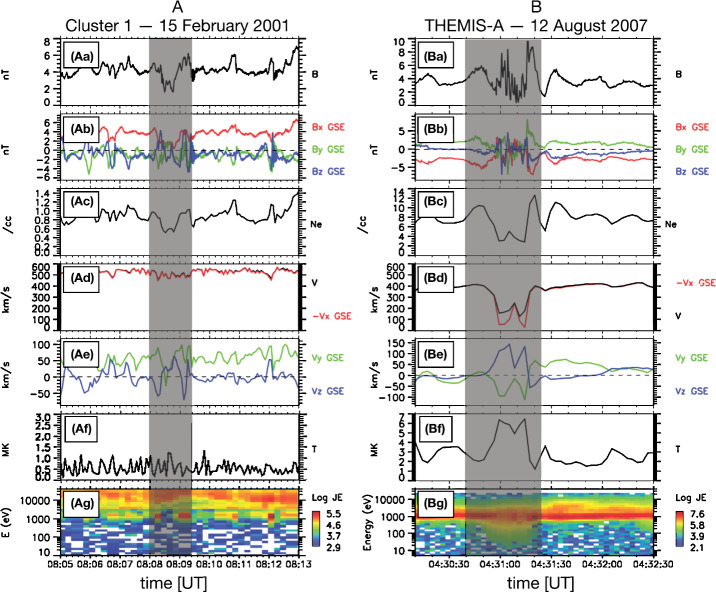
(**A**) An example of a foreshock caviton (grey shadow) observed by Cluster 1. (**B**) SHFA observed by THEMIS-A. Panels (a) to (g) in the figures have the same format as Fig. 18

In addition to waves, shocklets, and SLAMS, more recent studies have shown the existence of foreshock cavitons (Omidi 2007; Blanco-Cano et al. 2009, 2011; Kajdic et al. 2010; Kajdič et al. 2013) and SHFAs (e.g., Omidi et al. 2013; Zhang et al. 2013) in the foreshock. As shown in Fig. 22A, cavitons are crater-like structures with depleted magnetic field magnitude ($B$) and density ($n$) values in their cores, surrounded by rims where $B$ and $n$ are enhanced. On average both parameters decrease by about 50% of the ambient values (Kajdič et al. 2013). The temperature inside the cavitons is similar to the value in the surrounding plasma. Cavitons form deep inside the foreshock in regions populated by compressive waves and hot diffuse ions. Their formation mechanism includes nonlinear interaction of two types of waves, namely the transverse parallel propagating and fast compressive obliquely propagating waves (Omidi 2007). Cavitons are typically a few $R_{\mathrm{E}}$ in size (Kajdič et al. 2011; Omidi et al. 2013), have irregular shapes and are convected by the solar wind towards the bow shock. Solar wind speed and temperature do not change inside them. Their cores are permeated by diffuse ions, similar to their surroundings.

SHFAs were first observed in THEMIS data (Zhang et al. 2013) and were found in hybrid simulations (Omidi et al. 2013, 2014b). Their signatures in the spacecraft data (see Fig. 22B) are similar to those of ordinary HFAs (discussed in Sect. 5.3), namely central regions in which $B$ and $n$ are diminished when compared to ambient values and rims in which the two quantities are enhanced (Schwartz et al. 1985). In contrasts to cavitons, the temperature in the SHFA central regions is increased by several orders of magnitude and the solar wind flow inside them is strongly decelerated and deflected. SHFAs and HFAs are different because the former are not associated with solar wind current sheet interaction with the bow shock, while HFAs are. The proposed formation mechanism for SHFAs includes multiple ion reflection between foreshock cavitons and the bow shock (Omidi et al. 2013) as cavitons approach the shock and ion trapping by the cavitons. It was shown by Kajdič et al. (2017) that SHFAs are several $R_{\mathrm{E}}$ in size, supporting the fact that they grow from cavitons.

#### Jet Relation to Cavitons and SHFAs

Two mechanisms have been proposed that could relate foreshock cavitons to magnetosheath jets. The first one includes bow shock rippling. As shown in the simulations of Omidi et al. (2016), SHFAs can cause irregularities (rippling) in the bow shock which, as discussed above, provide good conditions for jet formation.

**Fig. 23:**
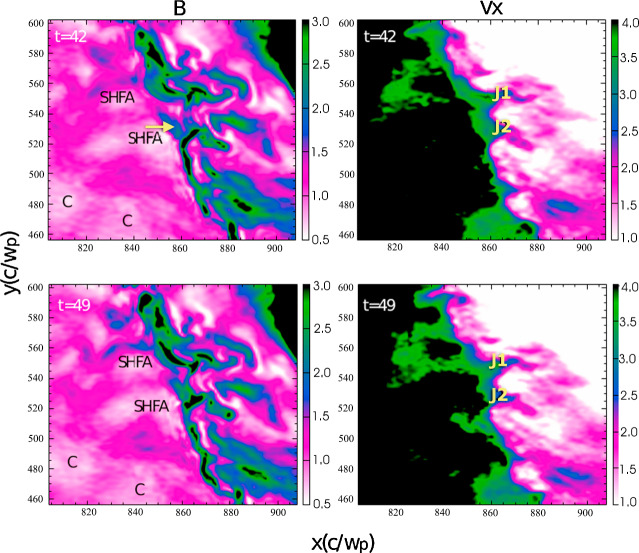
Global hybrid simulation results of the solar wind interaction with the magnetosphere. Colors indicate magnetic field magnitude (left panels) and $v_{x}$ (right panels) at two simulation times. The solar wind arrives from the left and the IMF is radial along $x$. The shock rippled structure is apparent in the left panels. The regions that appear in white are foreshock cavitons (labeled ‘C’) and SHFAs. The right $v_{x}$ panels show that there are upstream regions where the solar wind suffers deceleration. It is also possible to see two magnetosheath jets (J1 and J2). Details of the simulation run are given in Blanco-Cano et al. (2009) and Omidi et al. (2009). The unit $c/\omega _{\mathrm{p}}$ is the proton inertial length which near Earth has values ${\sim} 100~\mbox{km}$

The second mechanism has to do with the possibility that caviton and SHFA interactions with the bow shock cause decreases in the shock magnetic field strength, due to the diminished $B$ inside them. Figure 23 shows $B$ magnitude and $v_{x}$ from a global hybrid simulation at two different times. Results related to foreshock phenomena for this run have been described in Omidi et al. (2009) and Blanco-Cano et al. (2009). The panels on the left show the magnitude of the magnetic field. It is possible to see the rippled shock structure. The magnetic field strength is highly variable along the shock front and it also changes with time due to the shock reformation and due to the arrival of foreshock structures. Values of $v_{x}$ at the same times are shown in the right panels, showing that magnetosheath jets appear at two locations: $y \sim 550\, c/ \omega _{\mathrm{p}}$, and $y \sim 525 \, c/ \omega _{\mathrm{p}}$. Here $c / \omega _{\mathrm{p}}$ is the inertial length, with $\omega _{\mathrm{p}}$ equal to the plasma frequency.

The white regions just upstream of the shock observed in the $B$ panels correspond to SHFAs which have evolved from cavitons (denoted by C). The white structures closer to the shock are identified as SHFA and not cavitons because they have larger temperatures than their surroundings (not shown) in agreement with SHFAs. It is possible to see that the SHFA at $y \sim 520$ to $530\,c/\omega _{\mathrm{p}}$ at $t=42\,\omega _{\mathrm{p}}^{-1}$, merges into the shock disrupting it and resulting in a weaker field, with $B=2$ instead of $B=3$ as in adjacent shock regions (see arrow in top left panel of Fig. 23).

**Fig. 24:**
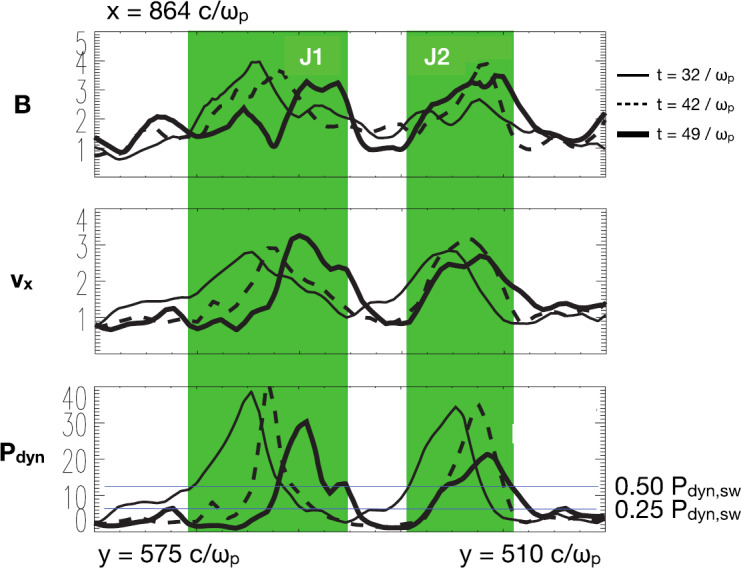
Magnetic field magnitude, $v_{x}$ and dynamic pressure $P_{\mathrm{dyn}}$ for the cut along $x=864\,c/\omega _{\mathrm{p}}$ in Fig. 23, for three times ($t=32/\omega _{\mathrm{p}}$, $42/\omega _{\mathrm{p}}$, $49/\omega _{\mathrm{p}}$) of the simulation run. Jets J1, J2 satisfying the criteria of Plaschke et al. (2013), $P_{\mathrm{dyn,msh}} > 0.50 P_{\mathrm{dyn,sw}}$, are marked with green

A cut through the simulation box along $x=864\,c/\omega _{\mathrm{p}}$, with corresponding values for $v_{x}$ and $P_{\mathrm{dyn}}$, is plotted in Fig. 24. Two jets are marked in that figure as J1, and J2. The shock weakening caused by the SHFA at around $y=530\,c/\omega _{\mathrm{p}}$ (J2) results in a less decelerated flow at $t=42\,\omega _{\mathrm{p}}^{-1}$ compared to an earlier time. At $t=49\,\omega _{\mathrm{p}}^{-1}$ the jet J2 is again more decelerated. This means that SHFAs may play a role in modulating magnetosheath jets evolution. Figure 24 also shows $P_{\mathrm{dyn}}$ values for three times. It is possible to see how the jets’ width can vary in time. Furthermore, jet signatures observed in $P_{\mathrm{dyn}}$ and $v_{x}$ can have different extensions. More work is needed in order to better understand the effect of cavitons and SHFAs on the shock structure and on jet production and evolution.

### Jet Origin Due to Solar Wind Discontinuities

As mentioned earlier, most jets have been observed downstream of the quasi-parallel shock under steady IMF, and hence likely have an origin linked to the foreshock. However, there are also jets observed downstream of the bow shock related to changes in the IMF orientation, i.e., solar wind discontinuities. A number of mechanisms of magnetosheath jet generation involving discontinuities have been suggested from case study investigations, namely rotational discontinuities interacting with the bow shock, HFAs, and foreshock bubbles (FBs).

**Fig. 25:**
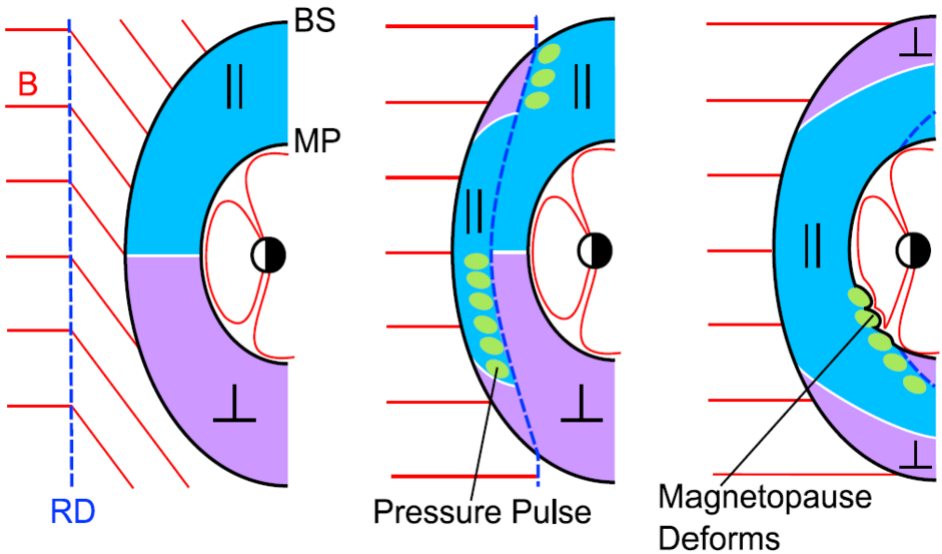
Sketch of pressure pulses occurring at a rotational discontinuity (RD) in the magnetosheath, where the geometry changes between quasi-parallel (blue) and quasi-perpendicular (purple). Figure 6 in Archer et al. (2012)

According to the fluid theory of shock-discontinuity interactions, the transmitted signature of a rotational discontinuity will evolve to form a new set of magnetohydrodynamic (MHD) discontinuities. Lin et al. (1996a,b) performed 1D MHD and 1D/2D hybrid simulations of this, showing these result in pressure pulses in the magnetosheath. Tsubouchi and Matsumoto (2005) showed similar results in one-dimensional hybrid simulations, arguing that particle kinetics dominate the generation and subsequent propagation of the pressure pulse. The simulations predict the largest amplitude pulses when the local geometry of the shock changes from quasi-perpendicular to quasi-parallel or vice versa (Lin et al. 1996b). Using simultaneous observations of the solar wind, foreshock and magnetosheath, Archer et al. (2012) showed numerous jets consistent with these simulations, illustrated in Fig. 25. A similar case study of a single jet was reported by Dmitriev and Suvorova (2012), though the authors interpreted the jet as a transient between the two equilibrium states, i.e., the quasi-parallel and quasi-perpendicular magnetosheaths.

**Fig. 26:**
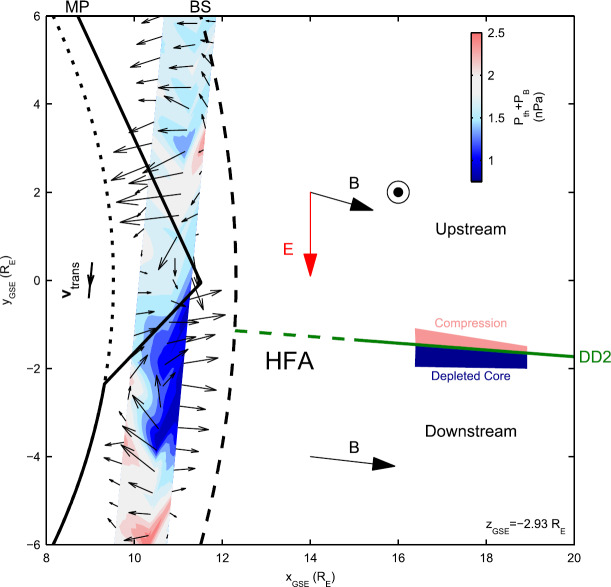
Traveling magnetopause deformation and magnetosheath flow pattern due to a hot flow anomaly upstream of the bow shock. Contours of the pressure (thermal + magnetic) which accelerated the flows are shown in colour. Figure 4 from Archer et al. (2014)

Savin et al. (2012) suggested that jets could be triggered by HFAs, which act as obstacles to the solar wind flow. HFAs occur when the solar wind motional electric field channels reflected ions specularly along solar wind discontinuities intersecting the bow shock (Burgess 1989; Thomas et al. 1991). This results in a hot ion population which expands forming a core region of depleted density and magnetic field and laterally drives pileup regions and shock waves on either side (Fuselier et al. 1987; Lucek et al. 2004a). Savin et al. (2012) propose that magnetosheath jets might occur downstream of HFAs as a means for achieving a local flow balance. Archer et al. (2014) presented multipoint observations of accelerated magnetosheath flows (both sunward and anti-sunward) downstream of an HFA, where the acceleration of magnetosheath plasma was directly driven by the pressure gradients present in the magnetosheath, as shown in Fig. 26.

FBs, first predicted by 2D kinetic hybrid simulations (Omidi et al. 2010) are transient phenomena caused by the interaction of suprathermal backstreaming ions with a discontinuity. Under certain IMF conditions this can lead to a concentration and heating of these ions on the upstream side of the discontinuity (Turner et al. 2013; Archer et al. 2015; Liu et al. 2015). The thermal plasma then expands against the solar wind, forming a hot core region of depleted density and magnetic field with significant flow deflections immediately upstream of the discontinuity, followed by a compressed region and possibly a shock. Upon arrival at the bow shock, the dynamic pressure variations of the FB accelerate magnetosheath plasma, firstly towards the intersection of the discontinuity with the bow shock and then antisunwards in response to the compressions and shock (Archer et al. 2015).

While the statistics show that the vast majority of magnetosheath jets are not associated with changes in the upstream IMF, Archer and Horbury (2013) showed that a minority of events do appear to be associated with solar wind discontinuities which cannot be explained by chance. Furthermore, Hietala and Plaschke (2013) found that some of the largest amplitude jets could not be explained by bow shock ripples and most of these were indeed associated with solar wind discontinuities. Therefore, while discontinuities do not constitute the dominant mechanism in magnetosheath jet generation, they cannot be entirely neglected.

### Other Possible Jet Origins

Evidence of reconnection within the turbulent magnetosheath plasma was provided, e.g., by Retinò et al. (2007). Using Cluster data these authors showed that thin current sheets with typical scales of a few ion inertial lengths (${\sim }100~\mbox{km}$) are found downstream of the quasi-parallel shock, and that reconnection can occur at them. Furthermore, observations reported by Phan et al. (2007) and simulations by Pang et al. (2010) have given evidence that reconnection jets can occur in the magnetosheath due to the compression of solar wind current sheets at the bow shock. More work is needed, however, to determine if reconnection at these sheets can be related to the magnetosheath jets discussed herein. If there is any relation between the small scale current sheets and the jets, it might just be for the smallest/shortest jets.

Another possible link between reconnection associated phenomena and magnetosheath jets was pointed out by Archer and Horbury (2013), who found that 18% of jets show a decrease in density but increase in velocity. These jets appear to be generally associated with the subsolar magnetopause. Thus, it is possible that they are related to FTEs which are thought to result from spatially and temporally limited reconnection events at the dayside magnetopause (Russell and Elphic 1978). FTE signatures include a decrease in density, increase in temperature, increase in magnetic field strength, and sometimes an enhancement in flow speed. A subset of the magnetosheath jets studied by these authors exhibits all of these properties.

Two other mechanisms that lead to high speed plasmas in the magnetosheath of Earth have been discussed by Chen et al. (1993), Lavraud et al. (2007), and Shue et al. (2009). Chen et al. (1993) and Lavraud et al. (2007) describe the so called slingshot effect that takes place in the flanks of the magnetosheath during low Alfvén Mach number solar wind conditions and may lead to plasma velocities of more than $1000~\mbox{km}/\mbox{s}$ in the antisunward direction. Lavraud et al. (2007) showed that this acceleration occurs due to enhanced magnetic pressure gradient and tension forces exerted on the plasma in a low-$\beta $ magnetosheath that results from the low Alfvén Mach number solar wind. MHD simulations and observations show that this acceleration is not symmetric, since higher velocities appear along the dawn and dusk flanks, while lower velocities are observed over the poles. The circumstances and the locations of this accelerated magnetosheath plasma do not coincide with those of magnetosheath jets so the magnetic slingshot effect may be discarded as a source of the magnetosheath jets discussed herein.

Shue et al. (2009) reported observations of anomalous flows in the subsolar magnetosheath during times of radial IMF. The THEMIS spacecraft first observed a small-scale (${\sim} 1\,R_{\mathrm{E}}$), fast ($-280~\mbox{km}/\mbox{s}$) antisunward flow—a magnetosheath jet—which was followed by a sunward flow. The authors concluded that the fast antisunward flow locally deformed the magnetopause which then rebounded, leading to plasma acceleration in the sunward direction. Shue et al. (2009) explain the fast antisunward flow in terms of concave bow shock shape that has been reported by Lin (1997), de Sterck et al. (1998) and Cable et al. (2007) to occur during radial IMF. However the authors did not have observations at the bow shock so they could not reach any final conclusions.

### Concluding Remarks

Due to the variety of jet characteristics, such as size, strength (in terms of $P_{\mathrm{dyn}}$), and region of occurrence, and due to the variety of favorable solar wind conditions (e.g., stable radial IMF versus IMF discontinuities), it is possible that only a combination of different sources might explain the entirety of observed jets. More work is needed to relate the large numbers of observed jets to the various proposed mechanisms and to achieve a better understanding of their generation mechanisms.

## Consequences for the Magnetosphere and the Ionosphere

This section addresses the response of the solar wind–magnetosphere–ionosphere system to transient and localized enhancements in the magnetosheath dynamic pressure. It begins with a discussion of the signatures expected when a jet or a plasmoid propagates through the magnetosheath towards the magnetopause. It then summarizes results from past case studies that directly associate such jets and plasmoids with magnetospheric and ionospheric phenomena. A discussion of previously reported phenomena that may also be related to the dynamic pressure enhancements follows. This section concludes by considering the significance of the enhancements to the overall solar wind–magnetosphere–ionosphere interaction.

### Theory

Basic plasma physics (e.g., Baumjohann and Treumann 1996) suggests the following scenario of jets propagating through the magnetosheath, impacting the magnetopause, and affecting the inner magnetosphere.

To reach the magnetopause, magnetosheath jets must plow through slower moving regions of the magnetosheath. Consequently shocks must precede fast-moving jets whilst fast mode waves precede slow-moving jets. The shocks or fast mode waves accelerate, compress, and enhance thermal and magnetic pressures within the ambient magnetosheath plasma. Upon reaching the magnetopause, the same shocks or fast mode waves and trailing jets launch fast and intermediate (or Alfvén) mode waves into a restricted region of the magnetosphere due to the limited extent of the jets transverse to the Sun–Earth line. The transmitted fast mode waves should enhance magnetic field strengths deeper within the magnetosphere. Theory predicts that the amplitudes of perturbations with short wavelengths decay much faster with distance from the magnetopause than those with longer wavelengths (Southwood 1968; Pu and Kivelson 1983).

Conservation of the first adiabatic invariant requires the fast mode waves to enhance particle anisotropies within the outer magnetosphere, possibly rendering pitch angle distributions susceptible to kinetic instabilities that can cause pitch angle diffusion, entry into the loss cone, and precipitation. The intermediate mode waves propagate along outer magnetospheric magnetic field lines to the ionosphere, where associated electric fields can excite convection. Both precipitating particles and field-aligned currents associated with the intermediate mode waves can cause density irregularities in the ionosphere, making possible radar observations of the convective flows.

Finally, the pressure variations associated with the jets must ultimately pass around the magnetosphere. Consequently, the enhanced thermal and magnetic pressures associated with the jets drive antisunward-moving ripples on the magnetopause. Once a jet passes, the local magnetopause is free to move back outward, and the local magnetosphere returns to its previous state.

### Observations

We consider now phenomena reported in conjunction with magnetosheath jets.

#### Magnetopause

**Fig. 27:**
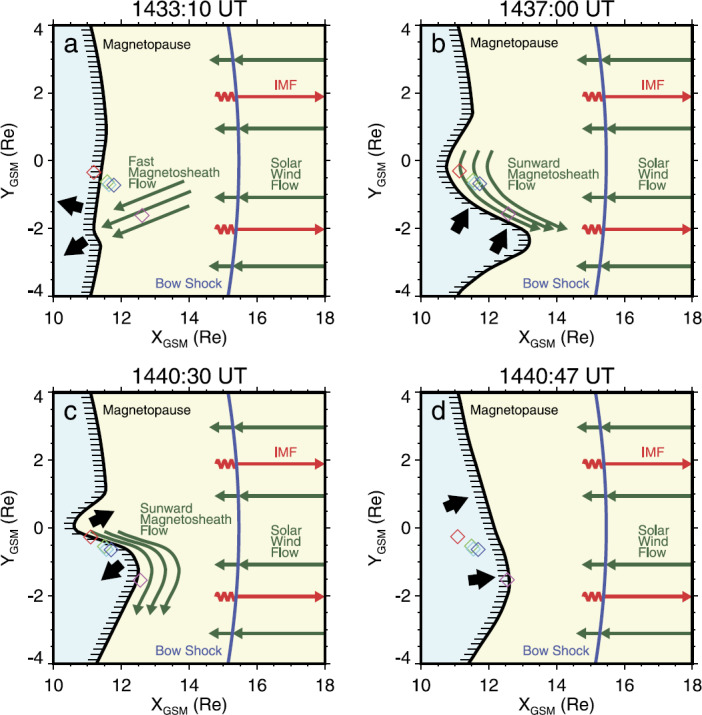
The response of the magnetopause to a magnetosheath jet. Diamonds represent the five THEMIS probes on a string-of-pearls orbit. The spacecraft are arrayed from THEMIS-B (nearest Earth) through THEMIS-C, THEMIS-D, and THEMIS-E to THEMIS-A furthest away. Large black arrows show the direction of magnetopause motion. Green arrows show plasma flows, red arrows the nearly radial IMF orientation. In panel (**a**), THEMIS-A observes a fast antisunward magnetosheath flow. In panels (**b**) and (**c**), a large amplitude boundary wave greatly distorts the magnetopause. In panel (**d**) the distortion diminishes. Figure 4 in Shue et al. (2009)

The jets are frequently associated with inward magnetopause motion (Amata et al. 2011; Hietala et al. 2009, 2012; Archer et al. 2012). Sometimes the large antisunward magnetopause and magnetosheath velocities are followed by equally large sunward velocities and a subsequent rebound in magnetopause location (Sibeck 1995), or even more complicated sequences (Dmitriev and Suvorova 2012). Figure 27 shows an example reported by Shue et al. (2009). Observed and model magnetopause normals often differ greatly, suggesting large amplitude ripples on the magnetopause surface (Shue et al. 2009; Amata et al. 2011; Dmitriev and Suvorova 2012).

Multipoint observations confirm the presence of such ripples, or surface waves, which propagate along the magnetopause (Archer et al. 2014). Plaschke et al. (2009) showed that magnetopause boundary oscillations occur more often around noon in local time during low solar wind cone angle conditions, when magnetosheath jets predominantly occur (see Sect. 3.2). They also proposed that jets may excite standing surface waves of the subsolar magnetopause, sometimes referred to as Kruskal–Schwarzschild modes (Plaschke and Glassmeier 2011).

**Fig. 28:**
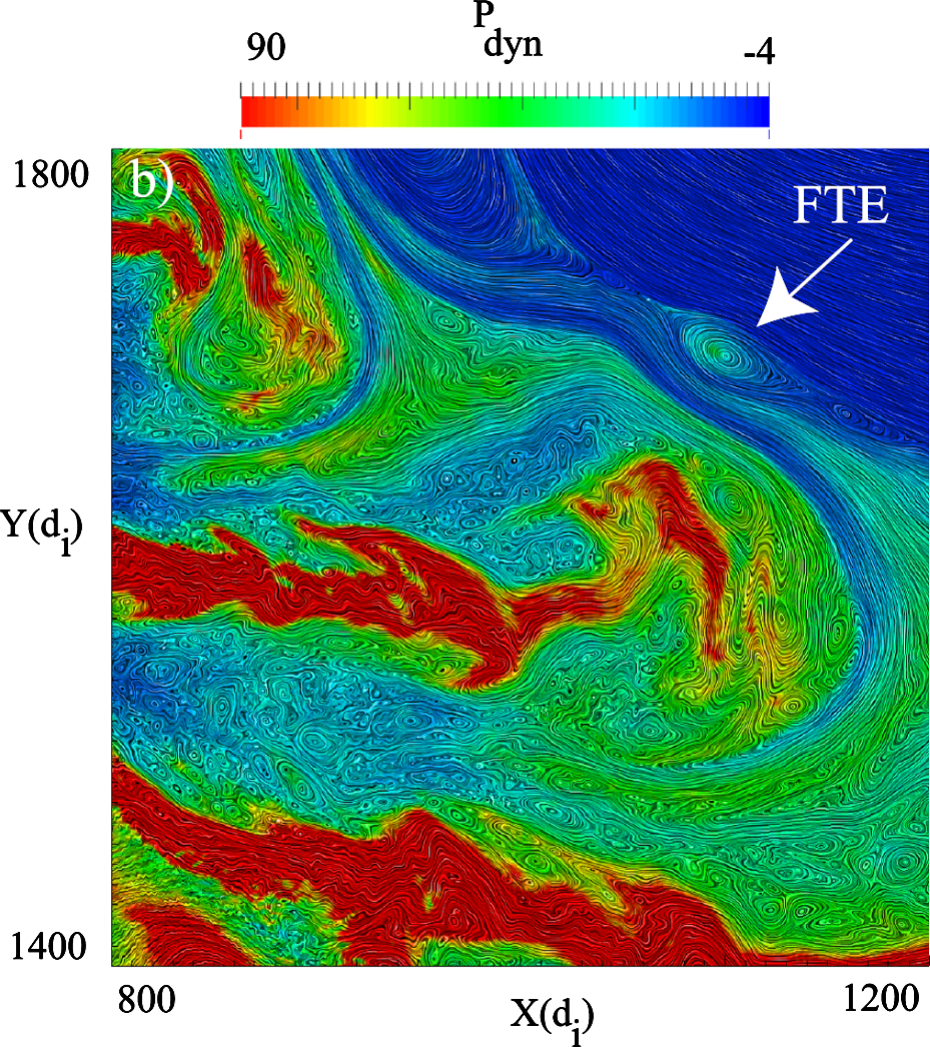
Simulation snapshot showing a magnetosheath jet, dynamic pressure is color coded, and a flux transfer event (FTE) at the magnetopause in response. Reprinted from Karimabadi et al. (2014), with the permission of AIP Publishing

Upon impact with the magnetopause, depending on the orientation of the magnetic field that is carried and pushed by the jet, magnetic reconnection may be triggered or ongoing reconnection may be suppressed. While the 2D hybrid simulations of Karimabadi et al. (2014) purport to show an FTE caused by reconnection in response to a magnetosheath jet, shown in Fig. 28, it should be noted that such phenomena cannot be accurately captured in 2D models, requiring 3D analysis.

#### Penetration of Magnetosheath Plasma

Observations from radially-aligned spacecraft have been used to show that the jets can be associated with density enhancements that propagate Earthward through the magnetopause boundary layers (Gunell et al. 2012; Karlsson et al. 2015b). On occasion, spacecraft closer to the Earth observe densities greater than those further away, suggesting either very large boundary distortions or perhaps ‘impulsive penetration’ of magnetosheath plasma (e.g., Lemaire 1977; Gunell et al. 2012), a laboratory-derived concept which is discussed in more detail in Sect. 8.2.

The enhanced flow velocities that are associated with the boundary waves can briefly energize and enable pre-existing cold plasma populations to be observed deep within the magnetosphere (e.g., Dmitriev and Suvorova 2015). Some magnetospheric compressions associated with jets leave spacecraft within the low-latitude boundary layer (LLBL), a region of enhanced plasma densities. Entries into the LLBL are more likely for jets with greater velocities and greater ratios of dynamic to magnetic pressure (Dmitriev and Suvorova 2015).

#### Magnetospheric Ultralow Frequency Waves

**Fig. 29:**
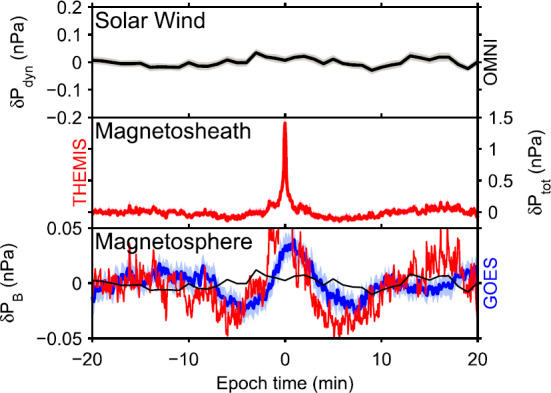
Superposed epoch analysis of solar wind dynamic pressure (top), magnetosheath total pressure (middle), and magnetospheric magnetic pressure at geostationary orbit (bottom) for magnetosheath jet events. The black and red lines in the bottom panel depict the quasi-static response to the respective upstream pressure variations based on the T96 model. Created from dataset introduced in Archer et al. (2013)

The jets cause transient and localized compressions of the magnetic field in the outer magnetosphere (Dmitriev and Suvorova 2012). Multiple sharp and impulsive peaks in magnetosheath flow and/or pressure can combine to elicit lower frequency, longer wavelength, weaker and smoother compressional and poloidal responses both near the magnetopause and deeper within the magnetosphere at geosynchronous orbit (Archer et al. 2013) (see, e.g., Fig. 29). The magnitude of this response can diminish as frequencies increase through those associated with local field-line resonances. However, Hietala et al. (2012) reported an absence of one-to-one correspondences between geosynchronous compressions and magnetosheath jets, neither in time nor in strength. They suggested that the smaller transverse size of the jets infer the weaker magnetic field pulsations observed in the magnetosphere.

Magnetosheath jets can also excite discrete frequencies of ULF waves within the magnetosphere in the absence of direct driving at such discrete ULF oscillations in either the solar wind or magnetosheath. These include local toroidal field-line resonances as well as compressional and poloidal Pc5 (2 to $7~\mbox{mHz}$) waves (Archer et al. 2013), the latter of which might be associated with Kruskal–Schwarzschild modes.

#### Energetic Particles

Lee et al. (2016) presented MMS observations of inversely energy-dispersed energetic ions of magnetospheric origin along with the anti-sunward jets in the magnetosheath. They suggested that a transient localized solar wind dynamic pressure pulse caused rapid inward motion of the magnetopause such that the energetic ions were energized via betatron acceleration, before escaping into the magnetosheath via reconnection at the boundary.

Magnetosheath jets may also influence particles in the radiation belts. For example it is known that the impact of solar wind dynamic pressure pulses can cause the loss of outer radiation belt electrons (tens of keV to several MeV) via magnetopause shadowing, due to the inward motion of the boundary, and outward radial transport, due to ULF waves (e.g., Turner et al. 2012; Xiang et al. 2016; Ni et al. 2016). Magnetospheric ULF waves can additionally transport and even accelerate outer radiation belts electrons (Elkington 2006). However, these processes have yet to be observed directly in connection with magnetosheath jets.

#### Ionosphere

Turning to the ionosphere, Hietala et al. (2012) reported a case study in which transient flow channels occurred in the high latitude ionosphere during an interval in which magnetosheath flow/pressure jets were present. Since the IMF was radial and not southward, they associated flow channel observations with intervals of enhanced particle precipitation enhancing the ionospheric density irregularities needed for radars to observe convection, rather than with the bursts of reconnection often seen during intervals of southward IMF. Dmitriev and Suvorova (2012) reported a case study in which a magnetosheath jet was associated with a perturbation in ground magnetometers that spread from the southern dusk to northern dawn stations as a corresponding solar wind feature swept across the magnetosphere.

**Fig. 30:**
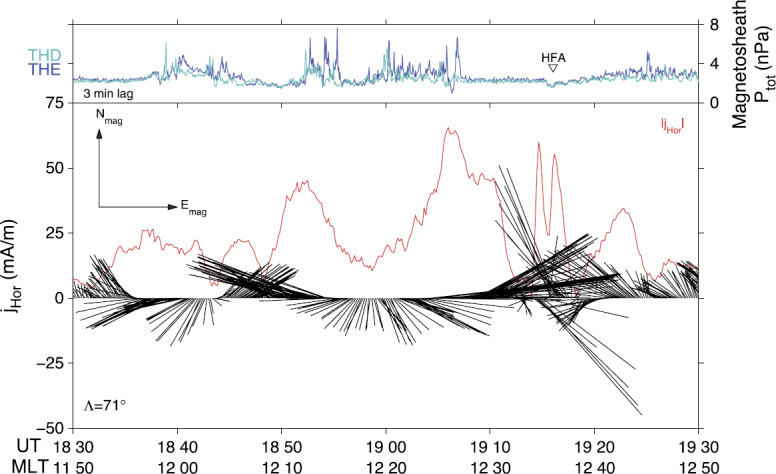
Pressure pulses in the magnetosheath elicit traveling convection vortices in the high-latitude ionosphere. The top panel shows the total pressure in the magnetosheath on 30 September 2008, as observed by THEMIS-D (turquoise) and THEMIS-E (blue). Black line segments in the bottom panel indicate the magnitudes and directions of the equivalent ionospheric currents derived from ground magnetograms as a function of time. Here geomagnetic north is upward and geomagnetic east to the right. The red curve depicts the horizontal current magnitude. Figure 8 in Archer et al. (2013)

As illustrated in Fig. 30, Archer et al. (2013) reported that magnetosheath dynamic pressure variations elicited ground magnetometer signatures whose equivalent ionospheric currents can be interpreted in terms of westward (antisunward) moving traveling convection vortices at pre-noon local times. Ground radar observations provided evidence for corresponding ionospheric flows. It should be noted that according to statistical studies (e.g., Sibeck and Korotova 1996; Kataoka et al. 2003), the occurrences of traveling convection vortices and the related magnetic impulse events have a preference for radial IMF orientation, similar to the magnetosheath jets.

### Discussion

Many of the magnetospheric and ionospheric phenomena described above had already been associated with the structures present in the solar wind and/or transient events generated by kinetic phenomena within the foreshock, including rippled magnetopause motion, transient magnetic field strength enhancements deeper within the magnetosphere, precipitation of magnetospheric particles, and transients seen by both high latitude ground magnetometers and radars (Sibeck et al. 1989a,b; Fairfield et al. 1990; Murr and Hughes 2003). Perhaps the magnetosheath jets represent the means by which information concerning these upstream phenomena are transmitted to the magnetosphere and ionosphere. Indeed, localized pressure gradients in the magnetosheath set up by transient foreshock phenomena can accelerate the magnetosheath plasma forming jets which in turn impact upon the space weather disturbances in the terrestrial magnetosphere-ionosphere system (Archer et al. 2014, 2015).

As noted by Plaschke et al. (2016), there are a number of ways in which transient magnetosheath dynamic pressure increases/jets can be geoeffective. They could initiate local magnetopause reconnection, enable the entry of magnetosheath plasma into the magnetosphere via impulsive penetration, transfer magnetosheath momentum to the magnetosphere via antisunward-moving boundary waves, enhance magnetopause shadowing by compressing the magnetopause, trigger particle scattering and precipitation into the Earth’s atmosphere by enhancing anisotropies in particle pitch angle distributions, enhance radial diffusion, particle energization, and loss at the magnetopause by initiating or enhancing ULF wave activity (Hartinger et al. 2013), and stir ionospheric convection by transmitting field-aligned currents and corresponding electric fields to the high-latitude ionosphere. Nevertheless, it should be noted that jets cannot represent the dominant mode of solar wind-magnetosphere interaction, except perhaps during the quietest times, because they exhibit no tendency to occur during intervals of southward IMF orientation when magnetospheric and ionospheric activity is greatest.

## Bursty Bulk Flows as a Possible Analogy to Jets

Magnetosheath jets consistently appear to be plasma entities that flow through an ambient plasma of different characteristics. They share this fundamental property with magnetotail bursty bulk flows (BBFs, Baumjohann 1993; Angelopoulos et al. 1994), to which they have not been compared to date. Jets and BBFs both appear to interact with ambient plasma by compressing it and pushing it away on their passage. However, while the BBFs have been studied observationally and by simulations (see, e.g., Sharma et al. 2008; Kepko et al. 2015; Wolf et al. 2009) for a couple of decades, the studies of magnetosheath jets are relatively recent. BBFs play an important role in transporting mass, energy and magnetic flux in the tail plasma sheet, as well as in accelerating the plasma, in populating the ring current and in providing the seed population for the radiation belt.

Comparative studies of magnetosheath jets and BBFs may unravel the fundamental physics governing plasma jet propagation, evolution, and impacts in different parameter ranges. In this section, we briefly summarize our knowledge of BBFs, keeping in mind their possible analogy to magnetosheath jets. We are particularly interested in: (1) the way the jets interact with the ambient plasma, (2) what controls their lifetime and penetration distance, (3) which analysis methods/techniques may be of interest in addressing the magnetosheath jet phenomenon.

### General Overview of BBFs

BBFs are fast plasma flows in the tail plasma sheet first observed by Baumjohann et al. (1989). They are observed as high-speed flows (at hundreds of km/s), lasting about $1~\mbox{min}$, grouped into roughly 10 to $20~\mbox{min}$ long sequences. Although BBFs are only detected during less than 5% of observation time, they are responsible for as much as 70% to 80% of magnetic flux, mass and energy transport in the tail plasma sheet during both quiet and disturbed conditions (e.g., Angelopoulos et al. 1992). The BBFs can formally be identified in different ways. Their definition was initially based on a flow criterion ($v > 400~\mbox{km}/\mbox{s}$ peaks embedded in ${>} 100~\mbox{km}/\mbox{s}$ flow region). A more physically-consistent definition is based on the magnetic flux transfer criterion: $E_{y} > 2~\mbox{mV}/\mbox{m}$, equivalent to the motion of a $B_{z} = 5~\mbox{nT}$ flux tube at $v = 400~\mbox{km}/\mbox{s}$ (Schödel et al. 2001). In the case of slowing-down inward-moving plasma tubes, the BBFs cannot be identified with the $v$-based definition for their entire lifetime, instead the $E_{y}$-based definition must be used.

Different methods agreed in that cross-tail spatial scales of the tail-aligned flow burst lie in the range of 1 to $5\,R_{\mathrm{E}}$ (Sergeev et al. 1996, 2004; Angelopoulos et al. 1997; Nakamura et al. 2001, 2004), so during most of its lifetime the individual flow burst represents a fast flow channel. Being on closed magnetic field lines and mapping to the ionosphere via precipitated (field-aligned accelerated) electrons, the BBFs auroral footprints (auroral streamers) provide a great help in BBF studies, allowing us to follow the BBF evolution over most of its active lifetime (until reaching the flow braking point), which is of the order of ten minutes.

### BBF Structure

The typical parameter behavior in and around the BBFs in the central plasma sheet has been studied in a dozen of superposed epoch type studies. They consistently describe a systematic organization and well-defined structure of the BBFs interacting with the ambient plasma (for a more comprehensive investigation see Ohtani et al. 2004; Liu et al. 2013).

**Fig. 31:**
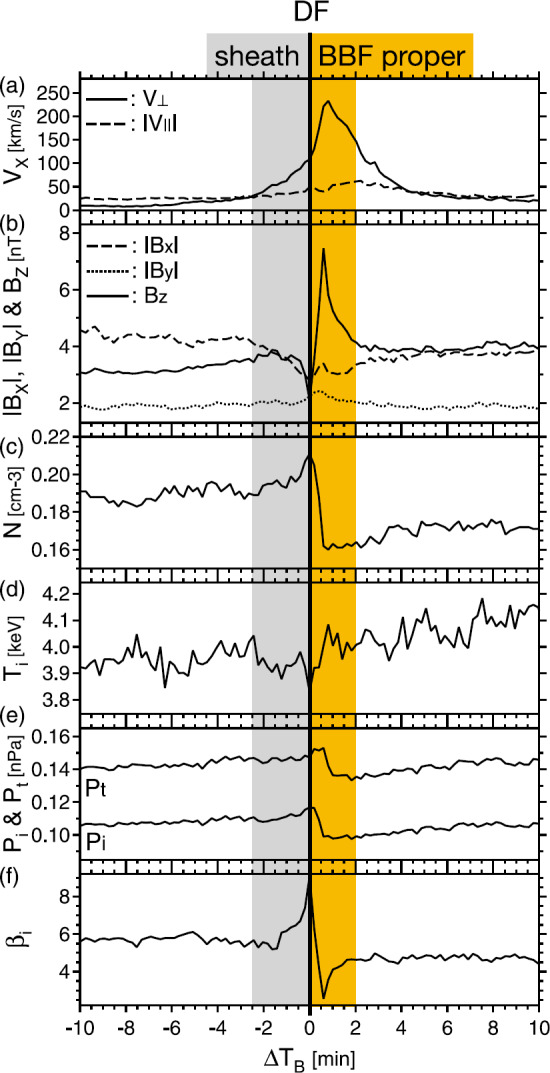
Superposed epoch analysis of BBFs, after Fig. 4 in Ohtani et al. (2004). Magnetic field and plasma observations are given in geocentric solar magnetospheric (GSM) coordinates. (**a**) $x$-component of the ion bulk velocity, (**b**) $x$, $y$ and $z$-component of the magnetic field, (**c**) ion density, (**d**) temperature, and (**e**) pressure. Panel (**f**) shows the ion plasma beta, which is given by the ratio between thermal and magnetic pressures

Figure 31, adapted from Ohtani et al. (2004), provides a superposed epoch analysis of flow bursts as they move earthward past the spacecraft. The figure shows a well-defined boundary called a “dipolarization front” (DF, Liu et al. 2013) which separates the BBF proper (shaded in orange) and the BBF sheath (grey). The DF is a narrow (${\sim} 800$ to $2000~\mbox{km}$) frontside boundary lasting a few seconds in spacecraft data during which typically the $B_{z}$ component increases sharply. The DF is a spectacular feature that occurs due to flow burst interaction with the ambient plasma (Sergeev et al. 2009). The region in front of the DF may be called the BBF sheath. This is the first feature related to BBFs that is observed by spacecraft. It lasts typically a few minutes in the spacecraft data during which the sheath plasma density gradually increases indicating a diamagnetic compression of ambient plasma in front of the earthward moving flux tube. The BBF proper behind the DF is a region of strongly increased magnetic field and depleted plasma density and pressure. The BBF proper exhibits high flow velocity and is often accompanied by elevated temperature and energetic particle flux.

**Fig. 32:**
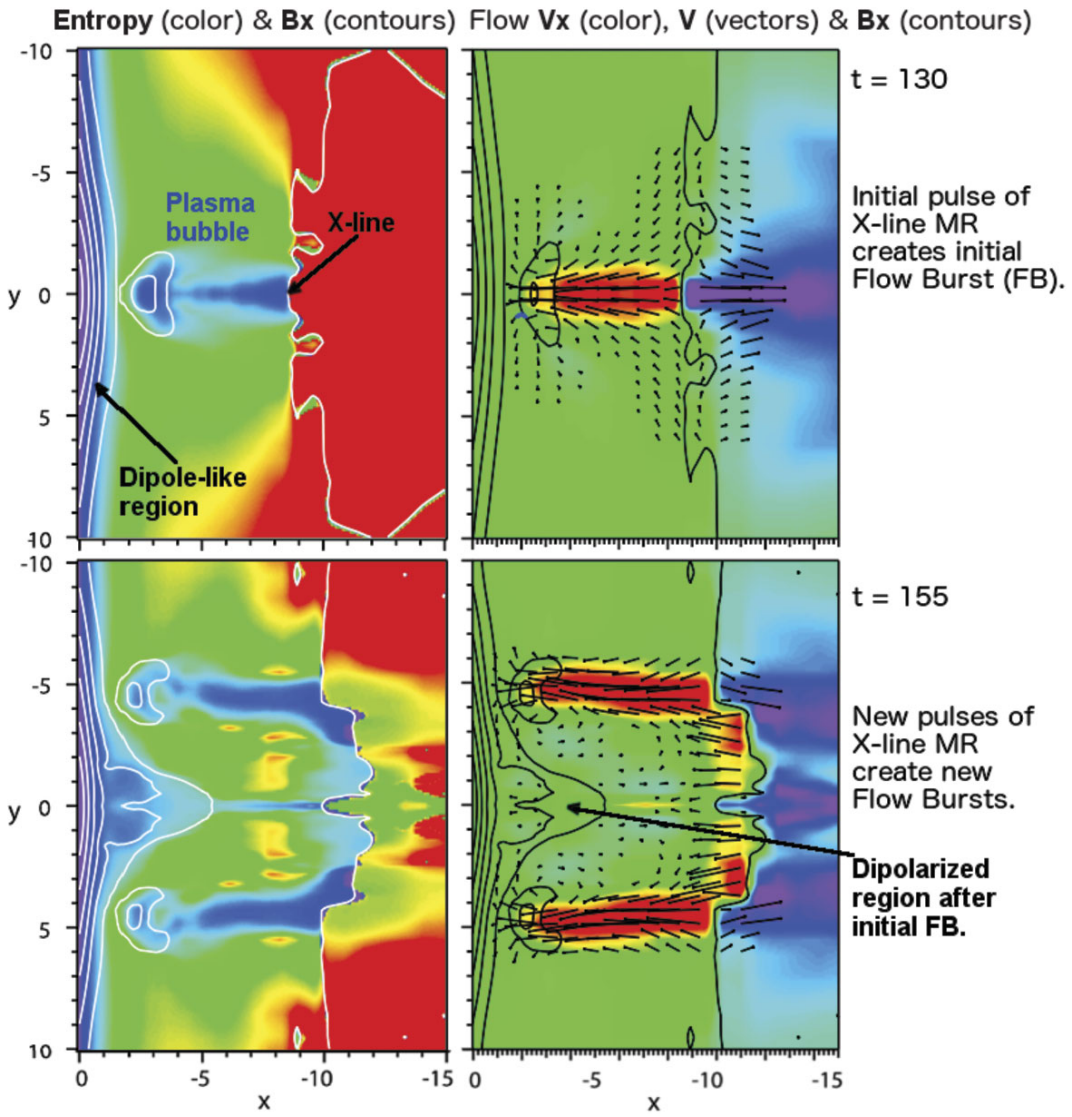
MHD simulation results, adapted from Birn et al. (2011). Note that a shifted distance is plotted as $x$ axis, with $x=0$ in the figure approximately corresponding to $x = -5\,R_{\mathrm{E}}$ in GSM in the real magnetotail

The BBF proper, the sheath as well as the DF are systematically reproduced in self-consistent MHD simulations (e.g., Birn et al. 2011) and Rice Convection Model simulations (e.g., Yang et al. 2011) of azimuthally localized flow channels, suggesting that the MHD approach captures well essential features of BBF interaction with the ambient plasma. In ideal MHD, the formation of a thin tangential discontinuity (DF boundary) can be understood as a general case of counter motion of two different (by origin) plasmas, which obey a frozen-in approximation. According to the simulations, the dipolarized but plasma depleted flux tube is generated by the reconnection process and represents the core of the BBFs (see Fig. 32).

**Fig. 33:**
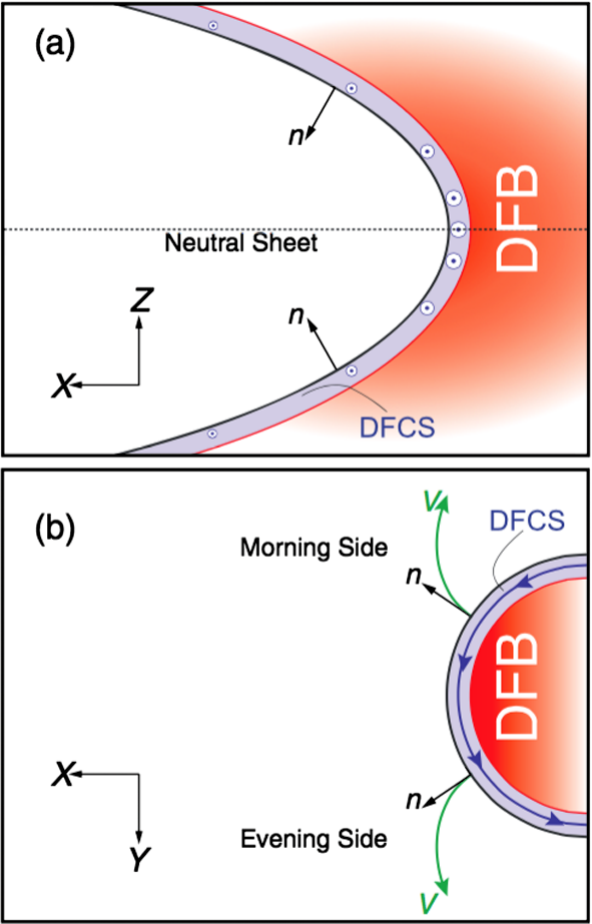
Illustration of the 3D shape of DFs, adapted from Liu et al. (2013). DFCS stands for dipolarization front current sheet, which demarcates the magnetic boundary of a dipolarizing flux rope or bundle (DFB, the strong magnetic field region led by a dipolarization front). The upper and lower panels show the meridional and equatorial cross-sections of a DF, respectively

Because 1-D models provide reasonable approximation for such thin DF current sheets, they are widely used in both, case- and statistical data studies. On most occasions 1-D models allow for evaluation of the local normal to the discontinuity by analyzing the $B$-time series (by applying the well-known Minimum Variance Analysis, see Sonnerup and Scheible (1998) for the method description and Liu et al. (2013) for the illustration of its massive testing and applications). Such analyses, applied to about a thousand observed BBFs, allowed to infer the average DF geometry, illustrated at the bottom of Fig. 33.

In a meridional cross section ($x$–$z$-plane, approximately corresponding to the magnetic field line plane) the DF nearly coincides with the magnetic field line having concave shape, and it can be understood as a tangential discontinuity. In the equatorial cross-section ($x$–$y$-plane, across the flux tube) the boundary is convex, as naturally expected for the finite size flow channel, so in total the DF surface has a saddle-like shape in 3D.

Knowing the front normal direction, one may infer many important properties of the flow burst. Particularly, using Cluster observations it was argued that the tangential electric field is small in the plasma frame moving approximately with the DF velocity, whereas a fairly large normal electric component $E_{n}$ (Hall electric field of tens of mV/m) is typically observed in the DF’s close vicinity, where the ion and electron motions are decoupled (Fu et al. 2012). Knowledge of the geometry is important in case studies of the force balance (Li et al. 2011; Runov et al. 2011; Hamrin et al. 2015; Karlsson et al. 2015a). Based on the known normal, it is also possible to organize the data to establish statistically the 3D picture of the sheath region, to understand how the ambient plasma tubes flow around the approaching concave DF, how big are the pressure gradients and how strong are the field-aligned currents in this sheath (see Liu et al. 2013).

### Tailward Flows

A few other elements of the BBF phenomenology have to be mentioned. The above description emphasized the standard earthward fast flows in closed flux tubes in the midtail plasma sheet region, ${<}20$ to $30\,R_{\mathrm{E}}$ downtail, which has been nicely covered by the past magnetotail missions. However, tailward flows are also observed, and they belong to two distinctly different types. One of them is the tailward flows carrying the southward magnetic flux (negative $B_{z}$), which are thought to be produced by the magnetic reconnection on the tailward side of the X-line. They may have either a closed magnetic topology (being a part of large scale plasmoid/magnetic flux rope), or are associated with open (recently reconnected) field lines. These BBFs are much less studied compared to the standard ones, although they may be a better analogy to the magnetosheath jets. The superposed epoch studies of such BBFs (Ohtani et al. 2004; Li et al. 2014) show the narrow DF, density depletion and many other features similar to earthward moving BBFs, except for their $B_{z}$ polarity and flow direction. Another subset of tailward flows are those exhibiting positive $B_{z}$ magnetic field component, which is frequently observed in the near Earth plasma sheet. Generally most of them are thought to be either the rebounds of previously earthward flow bursts (Ohtani et al. 2009), or a parts of flow vortex structures (Keiling et al. 2009), which is clearly seen at the concluding stage of the flow burst intrusion in the inner region, also in the MHD simulations (Birn et al. 2011).

### Flow Braking

**Fig. 34:**
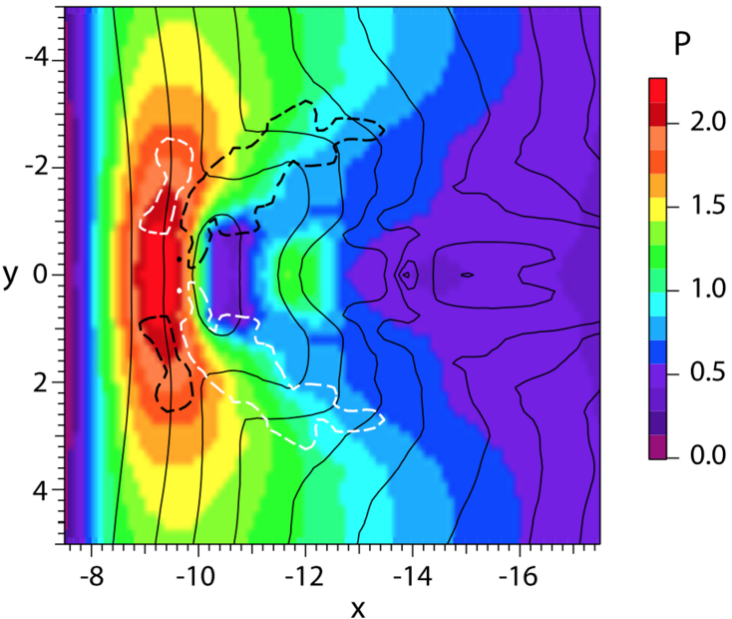
MHD simulation of pressure (color coded), $B_{z}$ (solid contours), and field aligned currents (dashed contours), adapted from Kepko et al. (2015)

The flow braking/diversion process is of great interest as a possible analogy to the magnetosheath jet penetration problem (see Sect. 6.2.2). Unfortunately, no coherent picture of this process yet emerges from observations, mostly because it requires a better (2D) spatial spacecraft coverage for which the modern spacecraft systems are still insufficient. Some elements have been occasionally studied in isolation, including the phenomenon of multiple rebounds: oscillating radial flows (see Panov et al. 2010) or flows turning from earthward to azimuthal (e.g., Kauristie et al. 2003; Lyons et al. 2015). The entire picture is tremendously complicated by the multiplicity of flow bursts, which come at different times in different places, and sometimes can interact with each other. Multiplicity of spontaneously generated flow bursts is also regularly reproduced in the MHD simulations, including both regional simulations (e.g., Fig. 32) and global simulations (e.g., Wiltberger et al. 2015). As discussed by Sergeev et al. (2014) and illustrated by Fig. 32, every flow burst brings additional magnetic flux and increases the plasma pressure in a local part of the inner region (see also Fig. 34), but due to the long relaxation time the effects of multiple flow bursts are integrated in the magnetosphere, providing a large-scale dipolarization attributed to the substorm current wedge effects during strong substorms. Another complication/difficulty is that the frozen-in behavior is violated in the drift-dominated inner region where different energetic component and different species move in different ways.

### Physics of BBFs in the Plasma Bubble Picture

As mentioned above, the flow bursts manifest as well defined plasma structures. Their origin as well as most of their properties are well captured in MHD simulations. There are two basic physics concepts which help to understand the BBF appearance and properties: transient magnetic reconnection and interchange motions (see, e.g., reviews by Wolf et al. 2009; Sharma et al. 2008; Birn and Hesse 2013).

Impulsive magnetic reconnection in the thinned portion of the tail current sheet generates fast outflows of recently-reconnected plasma tubes, which are density-depleted and dipolarized (enhanced $B_{z}$) plasma tubes, also known as plasma bubbles. The latter property comes from the fact that reconnection includes the lobe or outer plasma sheet magnetic flux tubes, which are relatively empty. Magnetic reconnection is currently considered as a major mechanism producing the bubbles. Some other possibilities have also been discussed, including the interchange instability in current sheet portions with local $B_{z}$ maximum (e.g. Pritchett and Coroniti 2010) or the plasma slippage effects in the regions of sharp changes of plasma tube entropy parameter (Yang et al. 2014). In reality these processes are hardly able to produce as strong depletions as reconnection can produce. The kinetic energy of fast flows ($1\,\mathrm{keV}$ energy equivalent for protons flowing at ${\sim} 400~\mbox{km}/\mbox{s}$) is not large compared to the thermal energy of the ambient plasma (having a proton temperature of several keV, and exceeding 10 to $20\,\mathrm{keV}$ during substorms) This motional resource is quickly exhausted during the flow burst interaction with the massive and high-pressure ambient plasma sheet. As a result, most of the BBFs propagation and, especially, its final stage are thought to be controlled by the bubble property of reconnection outflows.

**Fig. 35:**
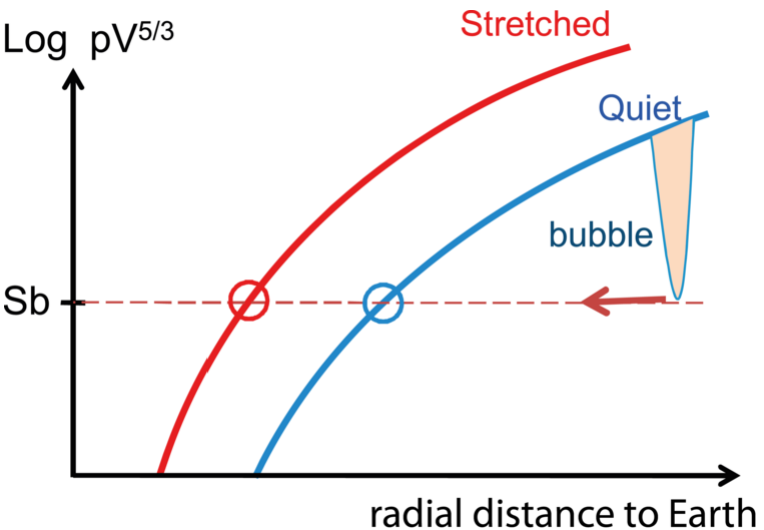
Illustrative sketch adapted from Sergeev et al. (2012). The color lines represent the entropy profile of the background plasma for different magnetotail configurations. Blue and red correspond to a quiet and stretched magnetotail, respectively. The plasma bubble continues to move earthward until its entropy ($S_{\mathrm{b}}$) equals the background plasma entropy. At this point the bubble will stop (marked by colored circles)

The plasma sheet contains closed magnetic field lines and has an approximately isotropic plasma pressure (Wang et al. 2012), which is nearly constant along the plasma tube. For a plasma nearly frozen into the magnetic flux tube the plasma tube entropy parameter ($S=PV^{5/3}$, where V is the volume of unit magnetic flux tube) is approximately conserved during the earthward contraction of a finite-volume tube. Its relationship to the entropy value of the surrounding plasma controls the tube polarization and its relative motion in the media. A bubble theory (in thin filament approximation, see Cheng and Lui 1998; Wolf et al. 2012) and MHD simulations of artificially produced plasma bubbles by Birn et al. (2004), further discussed in Birn and Hesse (2013), demonstrated that it is the bubble entropy value $S_{\mathrm{b}}$ relative to the background $S$ value (which is decreasing with decreasing distance in the standard magnetospheric models) and not the initial velocity of the bubble, which controls its final penetration distance. As illustrated in Fig. 35, the entropy model predicts that the bubble moves in the entropy-decreasing background until reaching the location where $S=S_{\mathrm{b}}$ (the penetration distance). This prediction was tested and confirmed using the radially separated THEMIS spacecraft (Dubyagin et al. 2011) and by analyzing the conditions controlling particle injections to the geostationary orbit (Sergeev et al. 2012).

### Magnetotail BBFs vs Magnetosheath Jets

**Fig. 36:**
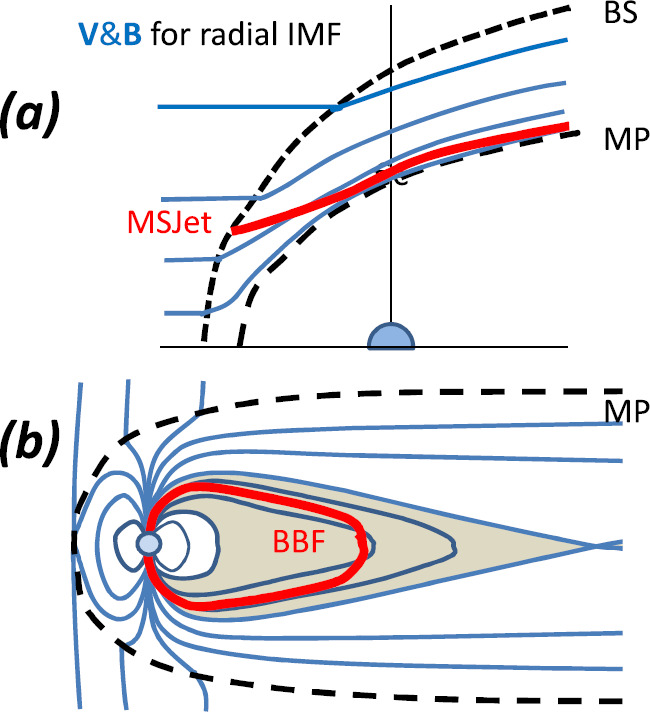
Illustrative sketch comparing BBFs with magnetosheath jets. The upper panel shows the velocity and magnetic field in the magnetosheath for radial IMF conditions. The lower panel shows the cross section of the Earth’s magnetosphere with a focus on the plasma sheet in the magnetotail (gray region). The red lines demonstrate the open and closed magnetic topology in the magnetosheath and plasma sheet respectively. While jets expand initially along the open magnetic tubes, BBFs are fast plasma movements in which the underpopulated magnetic tubes are embedded

As follows from this brief summary, the closed topology of the magnetic field lines in the plasma sheet essentially defines the evolution, compression, and inward penetration of the BBFs. In that respect the BBFs are drastically different from magnetosheath jets, whose development should be significantly less affected by the magnetic field. The major effects in the tail are the plasma tube convection (across $B$), and compression due to fast decrease of the finite volume of the plasma tube. These global constraints caused by closed plasma tube topology are highly important in the plasma sheet but not in the magnetosheath, where an open magnetic topology is expected (see Fig. 36).

The initial plasma states are very different: while the flow speed threshold for BBFs is ${\sim} 400~\mbox{km}/\mbox{s}$, the distribution of magnetosheath jet velocities peaks between ${\sim} 100~\mbox{km}/\mbox{s}$ and ${\sim} 200~\mbox{km}/\mbox{s}$ (see Fig. 13). The two tubes of flows act in different surroundings, as average ion temperature is $T_{\mathrm{i}} \approx 0.5~\mbox{keV}$ and ${\sim} 5~\mbox{keV}$ in the magnetosheath and plasma sheet, respectively. Hence, while kinetic energy and inertial forces are among the main players in the jets’ case, magnetic and pressure forces are the main factors in the plasma sheet BBFs.

A task for the future can be to test the criteria for jets based on electric field (magnetic flux transport) rather than on flow dynamic pressure, as is the case with BBFs. This way the jets could be detected even when they slow down closer to the magnetopause or when they are difficult to discern from surrounding plasma, for example in flanks of the magnetosheath, where the regular plasma flow is already fast.

Magnetic reconnection plays a major role in shaping the temporal sequence of flow bursts and the cross-tail jet structure of BBFs, and by forming the plasma bubbles (reconnection of empty flux tubes). The origin of magnetosheath jets is clearly different (see Sect. 5) and consequences of these differences still wait to be fully investigated.

## Other Plasma Environments

The magnetosheath jets should be a universally occurring phenomenon downstream of collisionless shocks. These may include planetary, heliophysical, and astrophysical shocks, as well as those in the laboratory, whenever shock ripples or suitable upstream discontinuities are present. In this section we discuss the jet-related investigations in these plasma environments, and our expectations based on the studies conducted in the Earth’s magnetosheath.

**Fig. 37:**
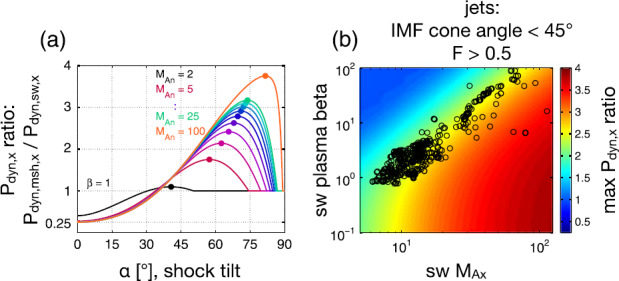
Parameter diagrams predicting ripple-generated jet dynamic pressure ratio as a function of upstream conditions; after Fig. 3 in Hietala and Plaschke (2013). (**a**) Dynamic pressure ratio as a function of shock tilt $\alpha $ for a range of Alfvén Mach numbers and $\beta = 1$. The maximum $P_{\mathrm{dyn},x}$ ratios are indicated with dots. (**b**) Modeled maximum dynamic pressure ratio and jet observations. The model calculations are illustrated in color. The black circles indicate the observed upstream conditions for the jets seen at the outer half of the magnetosheath ($F > 0.5$) during IMF cone angles ${<}45^{\circ }$ in the Plaschke et al. (2013) dataset. See also Figs. 7 and 9 in Sect. 3.2

Let us consider how the expected jet strength depends on the upstream conditions using the formula derived by Hietala and Plaschke (2013) for ripple-generated jets (Eq. (6), Sect. 5.2.2). By jet ‘strength’ we mean the dynamic pressure ratio between the downstream and upstream plasmas. Figure 37a shows the $P_{\mathrm{dyn,}x}$ ratio as a function of the shock tilt for $\beta = 1.0$ and a range of Mach numbers: $M_{\mathrm{A}n} = 2$ (low Mach number, e.g., interplanetary shocks), $M_{\mathrm{A}n}=5, 7.5, 10, \ldots, 25$ (Earth’s bow shock), and $M_{\mathrm{A}n} = 100$ (high Mach number, e.g., astrophysical shocks). Hietala and Plaschke (2013) found that there is a maximum attainable $P_{\mathrm{dyn},x}$ ratio for ripple-generated jets, marked with a dot. Hence, jets created by simple model ripples are expected to be weaker than this value. Figure 37b shows this maximum ratio from their model in color for a range of upstream $M_{\mathrm{A}n}$ and $\beta $ values: The maximum ratio increases with increasing Mach number and decreases with increasing beta.

The Plaschke et al. (2013) dataset of jet observations in the Earth’s magnetosheath contains also their upstream conditions. The black circles in Fig. 37b indicate these upstream values for jets recorded close to the bow shock during low IMF cone angle intervals (Sect. 3.1). We can see that the majority of the observed upstream conditions falls roughly on the contour of maximum $P_{\mathrm{dyn},x}$ ratio of 2.5; however, this behavior is simply due to general correlations in the properties of low cone angle solar wind at 1 AU. Therefore, to increase our understanding of jets in different parameter ranges, we need to investigate plasma environments other than the Earth’s magnetosheath.

### Solar System and Astrophysical Shocks

#### Example System: Mercury

Karlsson et al. (2016) have investigated whether structures analogous to magnetosheath jets or plasmoids exist in the Mercury magnetosheath. Due to limitations of the ion measurements on-board the MESSENGER spacecraft given the instrument placement and temporal resolution in the magnetosheath (Raines et al. 2011), determination of flow velocity and density on small enough scales was not possible. Instead Karlsson et al. (2016) looked for isolated magnetic field structures similar to those associated with both diamagnetic and paramagnetic jets/plasmoids at Earth (see Sects. 4.2 and 5.2.2).

**Fig. 38:**
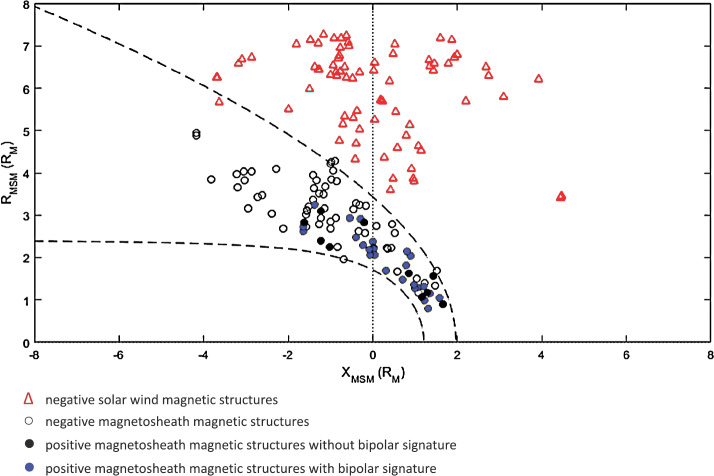
Location in $x$–$R$ MSM space (where $R=\sqrt{y^{2}+z^{2}}$, and MSM is the Mercury Solar Magnetospheric system) of the different types of isolated magnetic structures at Mercury’s magnetosheath (and close pristine solar wind). The dashed lines show the model positions of the magnetopause and bow shock (Winslow et al. 2013). Reprinted from Planetary and Space Science, 129, Karlsson et al., Isolated magnetic field structures in Mercury’s magnetosheath as possible analogues for terrestrial magnetosheath plasmoids and jets, 61–73, 2016, with permission from Elsevier

For isolated magnetic field structures with a clear decrease in field strength (‘negative structures’) Karlsson et al. (2016) concluded that these exist both in the pristine solar wind and in the magnetosheath (see Fig. 38), similar to Earth. Scale sizes and relative field decreases were also similar to the terrestrial diamagnetic plasmoids. Therefore, they are likely identical to ‘solar wind magnetic holes’ (e.g., Tsurutani et al. 2011) convected across the bow shock.

For structures with a magnetic field increase (‘positive structures’), the situation was different. Such structures were found at Mercury, but had some properties that were clearly different from the terrestrial paramagnetic plasmoids. A large fraction of the structures had a clear bipolar magnetic field signature, and were concentrated relatively close to the magnetopause (Fig. 38). Karlsson et al. (2016) concluded that these structures were likely FTEs. Since the magnetosheath end of an FTE flux rope should be associated with a lower plasma density than the surrounding magnetosheath, the positive magnetic field structures are not analogues to terrestrial paramagnetic plasmoids (but maybe the subset of terrestrial magnetosheath jets associated with FTEs, reported by Archer and Horbury (2013); see Sect. 4.2).

The fact that no paramagnetic plasmoids seem to exist in the Hermean magnetosheath is consistent with the idea that paramagnetic plasmoids are associated with SLAMS. It has been reported by Sundberg et al. (2013), that no SLAMS are generated at the quasi-parallel bow shock at Mercury. Rather, during steady solar wind conditions Mercury’s quasi-parallel shock is associated with cyclic reformation of large sections of the entire front. This is possibly due to the much larger ratio between the ion gyro radius and the size of Mercury’s magnetosphere, in comparison to the respective ratio (system size) at Earth.

#### Discussion and Conclusions on Planetary Bow Shocks

This leads us to the following conclusions regarding the upstream parameter dependence of (ripple-generated) jets at planetary bow shocks. The mechanism proposed by Hietala et al. (2009) to generate magnetosheath jets (Sect. 5.2), is valid for all rippled shocks regardless of magnetic field obliquity. Shock rippling requires that the Mach number is high and in the case of the quasi-parallel geometry, that the scales of upstream compressive structures are not too large compared with the system size. In the case of Mercury, strong waves similar to the ubiquitous “30 second” compressive waves in the Earth’s foreshock (Sect. 5.1) seem to occur more sporadically due to a weak bow shock that reflects few ions, and to the fact that there is not enough time for wave growth and steepening due to the small size of the foreshock (Le et al. 2013). When such waves do occur, due to the small system size they seem to lead to reformation of large sections of the shock (Sundberg et al. 2013). Therefore, the Hermean shock may be less rippled, leading to less structure in the magnetosheath, with few or no jets and/or paramagnetic plasmoids at all.

We know that ULF waves exist upstream of all planetary bow shocks in the heliosphere as well as in front of some comets (e.g., Wilson 2016). The characteristics of these waves, their evolution, and influence on the bow shocks are tailored according to the different system parameters, i.e., shock Mach number, IMF orientation, plasma beta, system size, etc.

Venus and Mars have small system sizes, and relatively high Mach numbers ($M_{\mathrm{A}} \sim 6$ and ${\sim} 10$, respectively). Thus it is expected that ion reflection will be stronger than in the case of Mercury, leading to wave generation, and possibly some steepening and development of upstream transients (shocklets, cavitons, and SLAMS; Sects. 5.2.1 and 5.2.3). Recently Halekas et al. (2017) have shown using MAVEN data that SLAMS can occur upstream of the Martian bow shock for near-radial interplanetary magnetic fields, reconfiguring the Martian bow shock. Similarly, Collinson et al. (2012) have observed SLAMS upstream of the bow shock of Venus. More work is needed to determine how often these waves can steepen, the possibility of shock rippling, and the existence of magnetosheath jets. The effect of mass loading should also be addressed.

At Saturn and Jupiter the system size is more than 100 times larger than for Earth, and their bow shocks are strong with $M_{\mathrm{A}} > 13$ in both cases. The observed waves in these foreshocks show strong compression (Bertucci et al. 2007), and their interaction with the bow shock might lead to a highly rippled shock structure. Yet the typical Parker spiral direction of the IMF at the giant planets is very oblique, and the dayside shock is most of the time quasi-perpendicular, with the quasi-parallel shock located at the flank regions. Therefore it is not clear how often steepened waves arrive at these shocks leading to rippling and reformation. Nevertheless, in the event described by Masters et al. (2013), Cassini spacecraft crossed Saturn’s bow shock during $M_{\mathrm{A}} \sim 100$, electron $\beta \sim 10$, and field-aligned flow. As we can see from Fig. 37b, these conditions would be ideal for the generation of strong jets at bow shock ripples.

#### Interplanetary Shocks

Interplanetary (IP) shocks in the solar system are driven by Interplanetary Coronal Mass Ejections (ICME, Sheeley et al. 1985) and Stream Interaction Regions (SIR, Gosling and Pizzo 1999). IP shocks differ from their planetary counterparts in that they have much larger curvature radii and usually they exhibit smaller Mach numbers than, for example, Earth’s bow shock (e.g., Gosling 1983). Statistical studies (e.g., Blanco-Cano et al. 2016; Kilpua et al. 2015) show that IP shocks tend to exhibit magnetosonic Mach numbers less than ${\sim}4$ but typically between 1 and 2. Kajdič et al. (2014) showed that about 50 % of IP shocks are supercritical, meaning that they reflect particles to some degree. It seems then that IP particle foreshocks should be fairly easy to observe, although the reflected particle fluxes are expected to be less intense due to shocks’ low Mach numbers.

Blanco-Cano et al. (2016) found suprathermal ions to be a common feature upstream of IP shocks. However most of the ICME driven foreshocks reported by the authors lasted of the order of half a day or more in the spacecraft data and were estimated to have typical extensions of 0.1 AU. This raises a question whether observations in such cases correspond to ion foreshocks similar to those observed at planetary bow shocks, these particles are part of Solar Energetic Particle (SEP, e.g., Schwenn 2006; Reames 1996) events in the suprathermal energy range (between ${\sim} 1~\mbox{keV}$ and several hundred $\mbox{keV}$), or a combination of both. Although Blanco-Cano et al. (2016) could not look at any ion distributions, in the past literature ion distributions upstream of IP shocks were reported almost exclusively to be isotropic (e.g., Gosling 1983). Since such distributions are typical of SEPs and of planetary foreshocks upstream of almost parallel bow-shocks, it is not possible to say what kind of ion foreshocks were actually observed by Blanco-Cano et al. (2016). There are only three papers reporting other kinds of ion distributions upstream of IP shocks, namely Tokar et al. (2000), Viñas et al. (1984) and Kajdič et al. (2017).

ULF waves upstream of IP shocks, similar to those at Earth, have been regularly observed (Viñas et al. 1984; Russell et al. 1983; Tsurutani et al. 1983; Kajdič et al. 2012). However the main characteristic of these waves is that they are only weakly compressive and almost never steepen into shocklets. In fact only Lucek and Balogh (1997) and Wilson et al. (2012) observed shocklets upstream of IP shocks, while SLAMS have not been reported at all. This is again probably due to relatively low Mach numbers of IP shocks and consequently low reflected particle fluxes.

What is needed for magnetosheath jets production is shock rippling or upstream discontinuities (see Sect. 5). Szabo et al. (2001) and Neugebauer and Giacalone (2005) showed evidence of strong IP shock rippling. Interestingly, Szabo et al. (2001) found that smaller and slower magnetic clouds (a subset of ICMEs) may drive more corrugated IP shocks. Furthermore, Krauss-Varban et al. (2008) used hybrid simulations to show that foreshock phenomena such as rippling can occur at IP shocks that are more oblique (i.e., less parallel) than the respective geometry at Earth due to their much larger spatial extent. Additionally, inhomogeneities in the solar wind may contribute to IP shock undulation.

Thus, although at IP shocks the favourable conditions for magnetosheath jet generation in terms of Mach numbers are rarely observed, there is certainly no reason to think that they do not occur at times. Investigating this is one of the tasks that should be carried out in the near future.

#### Termination Shock

As Voyager 1 and 2 crossed the heliospheric termination shock at the edge of the solar system (Jokipii 2008), their observations revealed a rippled, supercritical (${M_{\mathrm{ms}}} \sim 10$) quasi-perpendicular shock (Burlaga et al. 2008). Hence some structuring of the heliosheath due to the rippling would be expected.

Opher et al. (2003, 2004) have also investigated the interaction between the termination shock and the heliospheric current sheet. Their MHD simulations revealed formation of a high speed ‘jet-sheet’ in the heliosheath (see Fig. 2a in Opher et al. 2003). The authors concentrated on the behaviour of this jet deeper in the heliosheath and possible acceleration by the de Laval nozzle effect. However, the origin of the jet and its converging streamlines is a ripple on the shock, probably caused by the heliospheric current sheet.

#### High Mach Number Shocks

Some astrophysical shocks, such as the supernova remnant shocks thought to be the main source of cosmic rays, are expected to feature very high Mach numbers is the range of $M_{\mathrm{A}} \sim 20$ to 100 (see, e.g., Ghavamian et al. 2013, and the references therein). It has become evident that shocks are more structured than was previously recognized, so that a conventional plane wave description is not sufficient for studies of, e.g., particle acceleration. Extended, varying shock fronts can display large scale rippling implying locally quasi-parallel sections where jets might form due to foreshock effects. The mechanism proposed by Hietala et al. (2009) is also valid for a smoothly undulating high Mach number shock that stays perpendicular at each point. In astrophysical context, the high speed jets and non-thermal structuring of the downstream region can act as seeds for the magnetic field amplification and particle acceleration scheme proposed in Giacalone and Jokipii (2007), even for smooth upstream plasma. The bow shocks of Saturn (and Jupiter) can act as Solar System analogues to extended, high Mach number astrophysical shocks while still being accessible to in situ observations (e.g., Masters et al. 2011, 2013).

### Laboratory Plasmas

As was discussed in Sect. 6.2.2, one possible way the jets may interact with the magnetosphere is via impulsive penetration (e.g., Lemaire 1977; Lemaire and Roth 1991; Echim and Lemaire 2000). The penetration of plasma clouds or plasmoids across abrupt magnetic barriers has been studied in the laboratory since the 1950s, when Bostick (1956) performed the first such experimental study and coined the phrase ‘plasmoid’, defined as a ‘plasma-magnetic entity’.

A large number of experiments have shown that plasma clouds or beams (‘plasmoids’) will penetrate into the strong magnetic field region by a fast diffusion of the magnetic field into the plasmoid. This is accompanied by a self-polarization (by a charge separation within the plasmoids), resulting in an electric field in the $-\mathbf {v}\times \mathbf {B}$ direction (Bostick 1956; Baker and Hammel 1965; Lindberg 1978; Ishizuka and Robertson 1982), allowing the plasmoid particles to $\mathbf {E}\times \mathbf {B}$ drift across the magnetic field.

On the other hand, in several experiments no self-polarization was observed. Rather, the plasmoids penetrated the strong magnetic field region by magnetic expulsion, where the kinetic energy density of the plasmoid was enough to displace the magnetic field lines as it penetrates (Song et al. 1990; Hurtig et al. 2005). Finally, in some experiments the plasmoids were not able to penetrate into the strong magnetic field region (Song et al. 1990; Hurtig et al. 2005), referred to as rejection.

**Fig. 39:**
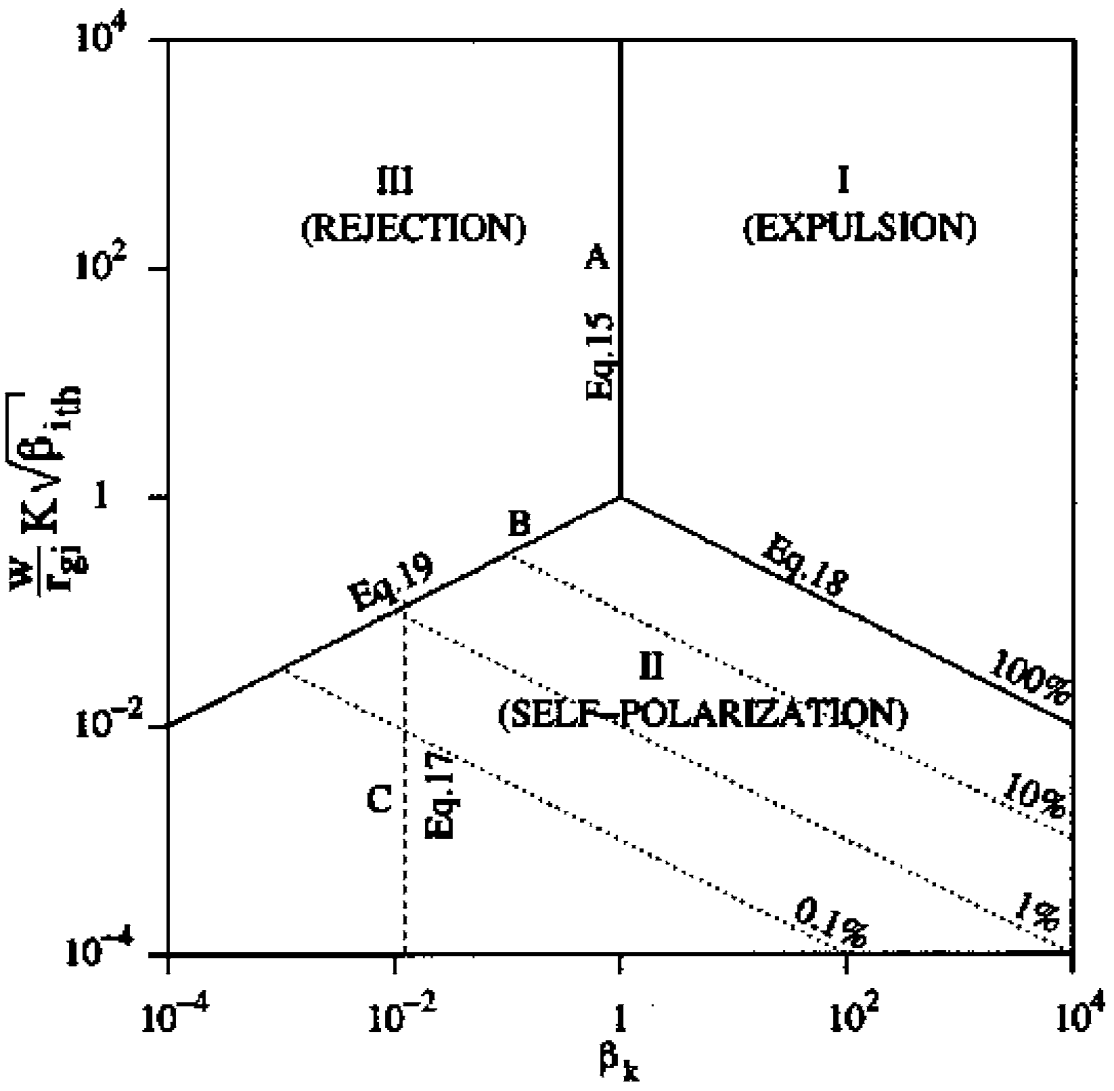
Parameter diagram predicting the interaction of plasmoids with a strong magnetic field region; see text for details. Reprinted from Brenning et al. (2005), with the permission of AIP Publishing

A systematic interpretation of all these experiments was suggested by Brenning et al. (2005), based on a scaling of the experimental parameters, energy considerations, and a model of how the diamagnetic currents on the surface of the plasmoids may trigger waves in the lower hybrid range. Such waves enable anomalously fast diffusion of the magnetic field into the plasmoids. Figure 39 shows their proposed interpretation. On the $x$-axis is the kinetic beta, defined as $\beta _{\mathrm{k}}=\frac{W_{\mathrm{k}}}{W_{B}}$, where $W_{\mathrm{k}}$ is the kinetic energy density of the plasmoid, and $W_{B}$ is the magnetic pressure in the strong magnetic field region. On the $y$-axis is a combination of the width $w$ of the plasmoid, normalized to the gyro radius $r_{\mathrm{gi}}$ (evaluated at the plasmoid velocity), $\beta _{\mathrm{thi}}$ (the thermal ion beta), and an empirical constant $K$ with a value of $2.3\pm 0.8$ (Brenning et al. 2005). The boundary between the ‘expulsion’ and ‘rejection’ regions of this parameter space is simply given by $\beta _{\mathrm{k}}$, indicating if the kinetic energy of the plasmoid is enough to displace the magnetic field lines. The smaller the plasmoid is, the larger the diamagnetic currents on its surface will be, and the boundary delineating the ‘self-polarization’ region marks the value at which the currents will excite the lower hybrid waves.

The scaling parameter map of Fig. 39 is in good agreement with the experimental results described above. Applying it to magnetosheath jets, we first note that the strong magnetic field region can be identified with the magnetopause. Second, we note that by definition they have $\beta _{\mathrm{k}} > 1$, since they have an excess kinetic energy density compared to the surrounding magnetosheath plasma which determines the pressure balance with the geomagnetic field at the magnetopause. Typical scale sizes of the order of $1\,R_{\mathrm{E}}$ of the jets gives values ${>}100$ for the $y$-axis (c.f., Karlsson et al. 2012). A prediction is therefore that one mode of interaction between magnetosheath jets and the magnetopause is that the jets may penetrate into the magnetosphere, displacing the magnetic field lines. This displacement may lead to magnetic field configurations with large magnetic field shear, which in turn may trigger local reconnection, similar to what happens in non-linear Kelvin–Helmholtz waves at the magnetopause.

## Outlook

In this section we collect and discuss some of the open questions that have arisen in the previous sections. Here we concentrate on jets in the Earth’s magnetosheath; for discussion on jets in other plasma environments we refer to Sect. 8. We have grouped the questions to five categories: origin, properties, evolution, microphysics, and magnetospheric effects. We start each category with a (non-exhaustive) list, and then expand on some of them in more detail.

### Open Questions on Jet Origin

- Investigating the different proposed generation mechanisms: do they produce jets and if so, what are the jet properties they predict?
- Do the jet properties imply different sources? (see Table 2 in Sect. 4).
- What is the role of solar wind turbulence, i.e., how steady has the IMF to be for jet occurrence?

#### Predictions of Different Proposed Jet Formation Mechanisms

A number of different mechanisms have been proposed for the formation of magnetosheath jets, and some of them are rather speculative (Sect. 5). To make progress, they would need to be investigated more systematically: which mechanisms actually produce magnetosheath jets? If so, what are the jet properties they predict? Are the predicted properties for different origins different or the same? For instance, is the first jet when the IMF changes to a low cone angle configuration different in origin and/or properties from the rest? Such investigations also require careful analysis of the bow shock structure in simulations, to relate the jets to plasma structures in the foreshock.

#### Solar Wind Conditions Leading to Jets: IMF Stability

The jet occurrence seems to depend predominantly on the combination of two factors: (1) IMF cone angle and (2) stability of IMF orientation (Sect. 3.2). The jets show a clear tendency to occur during low (${<} 45^{\circ }$) cone angles, and the IMF during the jet events is steadier than usual (see Fig. 8 in Sect. 3.2). These results point to the importance of a well-developed foreshock region.

Yet the real, pristine solar wind is never absolutely laminar, but features varying levels of fluctuations/turbulence. This leads us to ask how does the jet formation quantitatively depend on the level of ‘input’ turbulence? The mechanics of this have remained elusive, partly because it has not yet been investigated with modeling. Similarly, it is still unclear how the level of solar wind fluctuations quantitatively affects the jet occurrence and properties. This missing piece of information is an important gap in our knowledge and hinders our ability to predict jet occurrence under different solar wind conditions for space weather purposes.

### Open Questions on Jet Properties

- What are the 3D jet morphologies and aspect ratios, given that the (limited) studies so far seem to reach contradicting results (Sect. 4.3)?
- What are the signatures of jets in electron measurements? This would provide another means to identify jets in, e.g., measurements of planetary missions that tend to have limited ion data.
- What are the characteristics of particle distributions inside the jets?
- What is the magnetic topology and current structure related to the jets? How do these characteristics compare with those of BBFs (Sect. 7)?
- What is the flow pattern around the jets?
- How much mass, momentum, energy, and flux do jets transport?

#### Jet Contribution to the Magnetosheath Transport

What is the jet contribution to magnetosheath transport of mass, momentum, energy, and flux? Such a calculation requires the knowledge of the total and individual *volume* of jets. However, that information is quite hard to access with the observations, even with two to four spacecraft measurements available so far (Sect. 4.3). In the future, this could perhaps be improved upon with targeted cubesat studies. In contrast, it would be rather straightforward to extract this information from 3D simulations. Yet current computational capabilities do not allow 3D kinetic simulations of an Earth-sized system, so careful scaling analysis would be necessary.

#### Magnetic Topology

A crucial but under-investigated property affecting how jets evolve (Sect. 9.3) and eventually interact with the magnetopause (Sect. 9.5) is their magnetic topology. Karlsson et al. (2012) found that fast magnetosheath density enhancements are generally elongated along $\mathbf {B}$. Upon impact with the magnetopause, depending on the orientation of the magnetic field that is carried and pushed by the jet, magnetic reconnection may be triggered or ongoing reconnection may be suppressed. Unfortunately, neither of these phenomena can generally be accurately captured in 2D models, i.e., they require 3D analysis.

### Open Questions on Jet Evolution

- How do jet properties vary within the magnetosheath?
- Which jets make it to the magnetopause, and under what solar wind conditions is this possible?
- How do magnetic and pressure forces act on the jets: propulsion and braking?
- Are jets unstable to, e.g., Kelvin–Helmholtz, as the propagate in the magnetosheath?
- How different are results from (current) 2D simulations compared to 3D?

#### Variation of Jet Properties with Propagation Depth

Not all jets may manage to propagate from the bow shock to the magnetopause and have magnetospheric effects. The statistics of jet properties reported so far also show large variability, which could be partly due to variations and evolution within the magnetosheath. Recently, Dmitriev and Suvorova (2015) demonstrated that some variations could be seen in the properties (velocity, dynamic pressure) of one jet during its propagation using THEMIS multi-spacecraft observations.

**Fig. 40:**
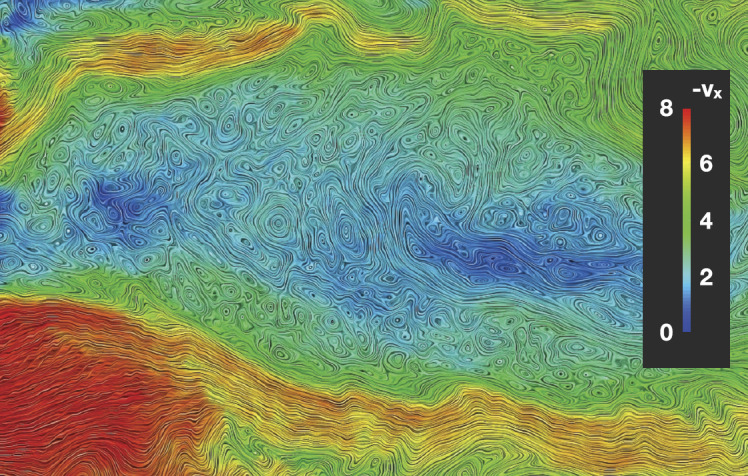
Turbulence in a region of 2D global hybrid simulation between two jets (Karimabadi et al. 2014). Magnetic field lines are visualized using the Line Integral Convolution (LIC) technique with the color corresponding to $-v_{x}$

Existing simulations (Sect. 4.5) suggest that propagation of the jets in the magnetosheath is a rather complex process determined by interaction with background flows, magnetic field, turbulence (see, e.g., Fig. 40), other jets, waves, and the stability of jets themselves. For instance, Omidi et al. (2016) recently reported that the length of the jets in the magnetosheath was larger for small Mach numbers, as the magnetosheath was less turbulent. Many of the jets in the simulations are seen to be unstable, e.g., against the Kelvin–Helmholtz instability as reported in Karimabadi et al. (2014). Other types of waves may also radiate away energy, adding to the braking of the flow (Sect. 4.4).

It is, thus, still largely unexplored how the jet properties vary with propagation depth in the magnetosheath. In particular, what are the distributions of jet properties near the magnetopause? These are the jets that will interact with the magnetopause and be responsible for the various magnetospheric effects. Furthermore, the extreme events (i.e., the events in the high velocity/density/dynamic pressure tails of the distributions) are likely to have the largest effects.

#### Determining the Solar Wind Conditions Responsible for Jet-Magnetopause Impacts

Currently, it is not known under which solar wind conditions it is possible for jets to penetrate all the way through the magnetosheath, yet that is what is needed to forecast jet-magnetopause impacts. Are conditions other than stable radial IMF required? Does, e.g., association with a solar wind discontinuity facilitate jet passage through the magnetosheath? Such a case would significantly increase the importance of this minority jet population, and also facilitate forecasting of magnetospherically relevant jet impacts. We also note that because the statistical studies (Archer and Horbury 2013; Plaschke et al. 2013) were done using observations from the deep solar activity minimum, it is not known whether jet occurrence and impact rates vary with the solar activity cycle.

#### 2D vs 3D Simulations

It is important to emphasize that 2D geometry of the simulation studies published so far (Karimabadi et al. 2014, Omidi et al. 2016, Hao et al. 2016a; Sect. 4.5) may play a significant role in restricting the magnetosheath flow geometry. First, the simulation results that typically show bent and deflected serpentine fast flows need to be reconciled with observations (Fig. 15; Sect. 4.3). Second, in the simulations the jets also lead to formation of anomalous flows in the magnetosheath, including sunward flows. While spacecraft have indeed detected such anomalous flow events when jets interact with the magnetopause (e.g., Shue et al. 2009; Amata et al. 2011), they might be exaggerated in the 2D models. Third, the 2D geometry may hinder the jet propagation and prevent it from reaching the magnetopause altogether. This is a severe limitation for investigating the magnetospheric effects of the jets.

### Open Questions on Energy Deposition in the Magnetosheath: Microphysics

- What is the role of the jets in the thermalization of the downstream region of quasi-parallel shocks?
- Are jets a significant driver of magnetosheath turbulence?
- What kind of waves do jets drive and emit?
- What is the nature of jet boundaries?
  - thin current sheets (conducive of reconnection) in the magnetosheath?
  - shock-like jet fronts resulting in particle acceleration?

#### The Role of Jets in Quasi-parallel Magnetosheath Turbulence and Thermalization

The quasi-parallel magnetosheath, penetrated by jets, is one of the most turbulent regions in near-Earth space. The enhanced level of turbulence leads to many important phenomena, such as generation of localized current sheets, magnetic reconnection that affects global and local magnetic field topology, and plasma heating and particle acceleration (e.g., Retinò et al. 2007; Karimabadi et al. 2014; Greco et al. 2008; Servidio et al. 2009; Chasapis et al. 2015; Wan et al. 2015; Vörös et al. 2016; Yordanova et al. 2016; Vörös et al. 2017). All of these processes precondition the magnetosheath before it meets the magnetopause. Jets could provide a significant source of free energy, for example through processes at the jet front and through the velocity shears associated with jets.

#### Nature of Jet Boundaries

**Fig. 41:**
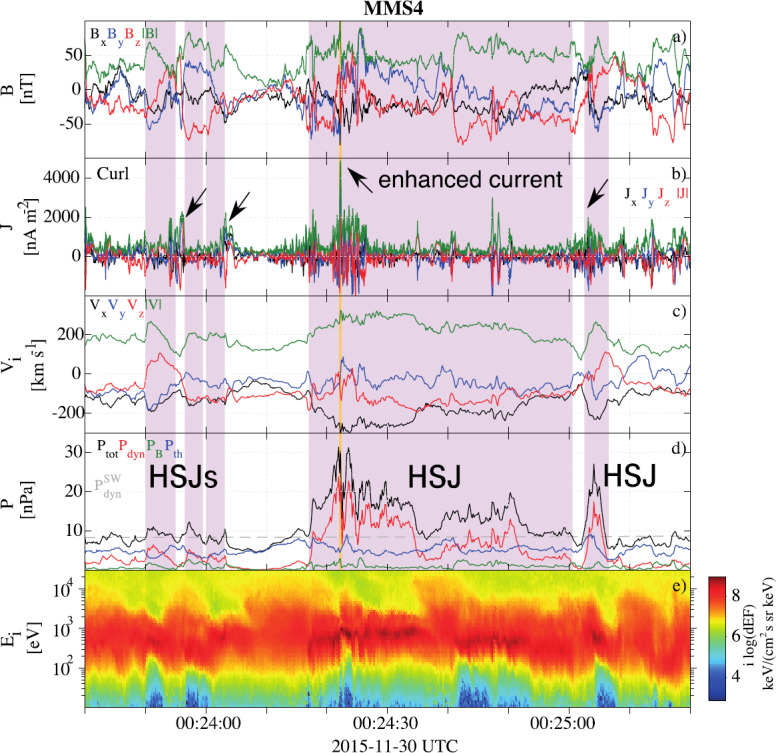
Magnetospheric Multiscale (MMS) observations of an intense current sheet associated with a jet. Adapted from Eriksson et al. (2016)

The micro-instabilities and particle dynamics at jet boundaries are largely unexplored. New Magnetospheric Multiscale (MMS) data (Eriksson et al. 2016; Plaschke et al. 2017) provide additional insight into the structure of jets and associated current sheets. Figure 41 shows an interval of quasi-parallel magnetosheath, embedded with jets (shaded regions). The jets, and their boundaries in particular, are associated with enhancements in current density. Eriksson et al. (2016) investigated the strongest ($j\sim 4900~\mbox{nA}/\mbox{m}^{2}$) current peak, and found it to be a thin, ${\sim} 3 d_{\mathrm{i}}$ sheet with sub-ion scale structure. The sheet was located between hot (magnetosheath-like) and less thermalized (solar wind-like) plasma. The current sheet featured a parallel electron beam, and electrostatic waves, but there was no evidence for on-going magnetic reconnection.

The jet fronts, somewhat similar to the well-studied magnetotail dipolarization fronts (Sect. 7.2), are also likely to be sites of interesting particle and turbulence dynamics. The jet bow wave seen in the simulations of Karimabadi et al. (2014) (Fig. 16) may steepen into a local shock, as seen in Cluster observations (Hietala et al. 2009, 2012). In fact, almost all of the jets identified by Plaschke et al. (2013) were super-Alfvénic in the GSE $x$-direction, and 14% featured supermagnetosonic flows in this direction. Hence, they should form a local shock upon interaction with the magnetosphere, or even in the outer magnetosheath. However, it has not been investigated how these local shocks affect magnetosheath heating and if they lead to, e.g., local particle acceleration.

### Open Questions on Role in Global Dynamics: Magnetospheric Effects

- Do jet impacts affect magnetopause reconnection and formation of FTEs? If so, is the effect only local or also global?
- Are the calculated impact rates (Sect. 3.2) consistent with inner-magnetospheric observations of, e.g., Pc5 wave power?
- Jets and IMF discontinuities: what is the dominant mechanism in the solar wind-magnetosphere interaction:
  - the solar wind discontinuity itself?
  - the jets formed due to the discontinuity?
  - the jets entirely unrelated to discontinuities?
- Could we use dayside auroral observations to support and complement in situ jet observations, similar to nightside magnetotail dynamics?

#### Possible Connection Between Jets and Local Magnetopause Reconnection

Jets frequently impact the magnetopause (Sect. 3.2) and are known to cause significant indentations to the magnetopause (Sect. 6.2.1). Could magnetosheath jets thus be geoeffective in terms of affecting local magnetopause reconnection? We find this likely because the jets may change the local conditions for reconnection in two ways: (i) Their high pressure impact may reduce the magnetopause current sheet thickness, hence triggering reconnection. (ii) They may change the shear angle between the magnetospheric and magnetosheath magnetic field lines, hence affecting the $\Delta \beta $-shear relation (Swisdak et al. 2010) which states whether or not asymmetric reconnection is suppressed by diamagnetic drift. The change in shear can be due to the magnetic field orientation within the jets (i.e., on the magnetosheath side). It can also be due to the magnetopause indentation caused by the jet’s dynamic pressure, as the orientation of the magnetospheric field lines is perturbed. Changes in the magnetic shear angle may then turn reconnection on or off.

Magnetopause reconnection in association with magnetosheath jets has not been systematically addressed to date, and thus it is not known how often it occurs. Since reconnection could be both turned on or off (or stay unaffected), a statistical approach should be applied to determine the overall effects. Neither is it known, what is the key property of the jet (dynamic pressure, magnetic field orientation) that would be required to affect reconnection? Are there specific solar wind conditions that lead to jets that trigger reconnection, so that it could be forecasted?

#### Possible Connection Between Jets and Dayside ‘Throat Aurora’

**Fig. 42:**
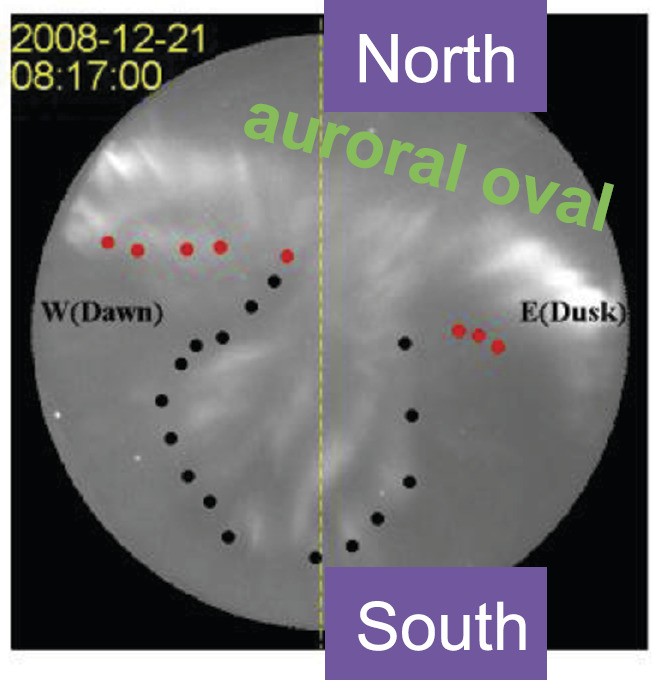
All-sky-imager observations of ‘throat aurora’. The equatorward boundary of the discrete aurora oval and the throat aurora are outlined by the red and black dots. Adapted from Han et al. (2017)

Recently, Han et al. (2015, 2016, 2017) found that dayside diffuse auroras observed near magnetic local noon often show discrete north-south aligned arcs extending from low latitudes towards the equatoward edge of the main auroral oval (Fig. 42). Note that north-south aligned auroral structures are considered atypical compared to the common east-west aligned forms. Because the arcs occurred near the throat region of the ionospheric convection, Han et al. (2016) called them ‘throat aurora’. The throat aurora are associated with magnetosheath particle precipitation and are on open field lines, i.e., they are thought to be associated with magnetopause reconnection. On the other hand, they occur against a background of diffuse aurora, which requires the presence of cold magnetospheric plasma.

The throat aurora occurrence shows only weak dependence on the IMF clock angle (small preference for northward IMF, in fact), which speaks against regular magnetopause reconnection. For the reader now familiar with characteristics of the magnetosheath jets presented in this review, the following properties of the throat aurora discovered so far have an immediate association with the jets: Their occurrence is enhanced for small (${<}45^{\circ }$) IMF cone angles compared to large cone angles—the occurrence pattern is remarkably similar to that of the jets (see Fig. 7). Based on magnetic mapping of the auroral observations, their north-south alignment is related to local Earthward protrusions of the magnetopause (indentations), which are inferred to be up to $3\,R_{\mathrm{E}}$ deep, comparable to jet induced indentations (Fig. 27; Sect. 6.2.1).

In the future, provided that the jet-throat aurora connection is verified, this kind of dayside auroral observations may prove to be a very important tool in studying jet-magnetopause interactions as they provide the means to remotely monitor the spatial and temporal evolution of the resulting magnetopause indentation in 2D.

## Concluding Words

As initially stated in the introduction section, the phenomenon of magnetosheath jets, discussed in this review, appeared in the literature approximately 20 years ago. During these years, to date, significant progress has been made. Basic properties and occurrence rates and locations have been determined. Upstream solar wind conditions favorable for the occurrence of jets have been identified, leading to the suggestion of some possible generation mechanisms. The relations between jets and a number of downstream consequences have already be shown, while a number of other consequences have been suggested to occur whose links to jets have not yet been rigorously proven. However, as evidenced by the long (and by far non-exhaustive) list of questions posed in the previous outlook section, many—even basic—characteristics and processes involving magnetosheath jets remain unknown. This represents a great and exciting challenge to the community, all the more as one of the established facts concerning jets is their importance in linking processes in the foreshock and at the bow shock to the magnetopause; their downstream impact and high rate of occurrence make them a key elements in the overall solar wind–magnetosphere–ionosphere interaction. Hence, we hope, that this review triggers future studies in this field, as it constitutes a useful starting point for those researchers not entirely familiar with the subject. We conclude this paper with an invitation to the reader to join our efforts in exploring the origins, nature, and consequences of jets downstream of collisionless shocks.
